# Exploring Guolin Qigong (Mind-Body Exercise) for Improving Cancer Related Fatigue in Cancer Survivors: A Mixed Method Randomized Controlled Trial Protocol

**DOI:** 10.1177/15347354241252698

**Published:** 2024-05-17

**Authors:** Sara L. K. Low, Gwo Fuang Ho, Bingkai Liu, Eng-Siew Koh, Yutong Fei, Chiah Shean Teo, Xiaoshu Zhu

**Affiliations:** 1 Western Sydney University, Penrith South, NSW, Australia; 2 University Malaya Medical Centre, Kuala Lumpur, Wilayah Persekutuan, Malaysia; 3 Pitié-Salpêtrière Hospital, Paris, France; 4 University of New South Wales, Liverpool, NSW, Australia; 5 Beijing University of Chinese Medicine, Beijing, China; 6 UCSI University, Malaysia

**Keywords:** Qigong, cancer, cancer survivors, fatigue, sleep, insomnia, depression, cortisol, mind body exercise

## Abstract

**Background:**

Cancer-related fatigue and its associated symptoms of sleep disorder and depression are prevalent in cancer survivors especially among breast, lung, and colorectal cancer survivors. While there is no gold standard for treating cancer-related fatigue currently, studies of mind-body exercises such as Qigong have reported promise in reducing symptoms. This study was designed to evaluate the feasibility and effect of Guolin Qigong on cancer-related fatigue and other symptoms in breast, lung and colorectal cancer survivors while exploring their perceptions and experiences of Guolin Qigong intervention.

**Methods:**

This is an open-label randomized controlled trial with 60 participants divided into 2 study groups in a 1:1 ratio. The intervention group will receive 12 weeks of Guolin Qigong intervention with a 4-week follow-up while control will receive usual care under waitlist. The primary outcome will be feasibility measured based on recruitment and retention rates, class attendance, home practice adherence, nature, and quantum of missing data as well as safety. The secondary subjective outcomes of fatigue, sleep quality and depression will be measured at Week-1 (baseline), Week-6 (mid-intervention), Week-12 (post-intervention), and Week-16 (4 weeks post-intervention) while an objective 24-hour urine cortisol will be measured at Week-1 (baseline) and Week-12 (post-intervention). We will conduct a semi-structured interview individually with participants within 3 months after Week-16 (4 weeks post-intervention) to obtain a more comprehensive view of practice adherence.

**Discussion:**

This is the first mixed-method study to investigate the feasibility and effect of Guolin Qigong on breast, lung, and colorectal cancer survivors to provide a comprehensive understanding of Guolin Qigong’s intervention impact and participants’ perspectives. The interdisciplinary collaboration between Western Medicine and Chinese Medicine expertise of this study ensures robust study design, enhanced participant care, rigorous data analysis, and meaningful interpretation of results. This innovative research contributes to the field of oncology and may guide future evidence-based mind-body interventions to improve cancer survivorship.

**Trial registration:**

This study has been registered with ANZCTR (ACTRN12622000688785p), was approved by Medical Research Ethic Committee of University Malaya Medical Centre (MREC ID NO: 2022323-11092) and recognized by Western Sydney University Human Research Ethics Committee (RH15124).

## Background

Cancer-related fatigue (CRF) has been increasingly recognized as an important symptom during and after treatment of cancer, impacting physical, mental, and emotional functions.^1^ CRF is a prevalent symptom among survivors of the most common types of cancer, including breast, lung, and colon cancer.^2,3^ It is a distressing symptom^3^ and one of the main reasons for the interruption of treatment or exacerbation of the disease.^2^ Between 74% and 90% of cancer survivors undergoing chemotherapy or radiotherapy^4-6^ experienced fatigue with adverse effect on other co-morbidity, quality of life, independent living, and work productivity.^3,7^ While fatigue is more prevalent within 6 months of receiving cancer treatment, cancer survivors including those who did not receive treatment continue to experience fatigue many years after treatment.^5^

Cancer diagnosis and its treatment also bring with it a host of physiological changes affecting various functions of the body including cortisol dysregulation associated with fatigue,^8-12^ sleep disorders,^8,13-15^ depression,^12,16^ anxiety, stress,^17,18^ functional disability,^12^ survival outcomes, and prognosis^13,18-21^ in cancer survivors.

The pathogenesis of CRF is still unclear, but might include proinflammatory cytokines, growth factors, circadian rhythm modulation, adrenal axis interruption, serotonin imbalance, afferent activation of the vagus nerve, and the production or use of abnormal adenosine triphosphate.^22^ Host factors not related to cancer and its treatment such as genetic, biological, psychosocial, and behavioral factor also play an important role in the development of CRF.^23^

CRF is also associated with various risk factors such as low performance status, undergoing chemoradiotherapy, insomnia, pain, neuroticism, depression and being female.^24^

It is further suggested that cancer-related fatigue is a composite of interacting responses to stress related to diagnosis and treatment, with the relationship of sleep disorders, depression, anemia, and inflammation featuring centrally for those under treatment as well as into the survivorship phase.^25^ CRF and a number of these factors and symptoms, including sleep disruption and emotional distress often co-occur as a symptom cluster,^26-28^ with some of these associations potentially linked to the underlying inflammatory biomarker changes such as cortisol level^29^ most associated with fatigue.^3,24,30^

Exploring symptom clusters is an important gap in symptom research to shed light on the common biological mechanisms^31^ and is gaining increased attention in the field of oncology in an attempt to improve the quality of life of patients diagnosed with cancer.^32^

Hence, there is a need to pay attention to the co-occurrences of fatigue, sleep disorder and depression as a symptom cluster in the evaluation and treatment of cancer survivors experiencing CRF.^3,26-28^

In spite of fatigue being the most common symptom reported by patients, they were least likely to report getting wanted help for it, perhaps due to the complexity of treating this common condition.^5^ Given the multifactorial nature of CRF which is still poorly understood, there is currently no “gold standard” treatment for CRF despite some approaches such as exercise, psychosocial interventions, and mind-body interventions being reported to yield beneficial effects in improving CRF.^23^

Emerging studies have shown that mind-body exercise such as Qigong can reduce fatigue, improve sleep, depression, anxiety, quality of life and cognitive changes associated with cancer as well as biomarkers of stress, cortisol level, inflammation and immune function in cancer survivors during and after treatment.^29,33-44^

The term “Qigong” is made up of 2 characters: Qi and Gong translated as the skill or practice to cultivate Qi. The concept of Qi, an inherent function and energizing essence of human being, is found in Traditional Chinese Medicine.^45^ Qi also refers to life force, vitality, or intelligence within a bio-electric body^46,47^ as well as the universe.^48^ There are a few thousand different forms of Qigong such as Tai Chi, BaDuanJin, Chan Chuang, Zhineng Qigong, and Guolin Qigong, all of which can be broadly classified into Chinese Medical Qigong, Daoist Qigong, Buddhist Qigong, Confucian Qigong, and martial arts Qigong. All forms of Qigong involve the recognition and cultivation of Qi to promote a balanced Qi and smooth flow of Qi in the body^49^ to enhance physical, psychological, and spiritual health through mind-body integration of specific movements, breathing techniques, and meditation.^50^ Mind-body integration of Qigong is also believed to promote health through the activation of neurohormonal pathways and other physiological mechanisms.^51^ Qigong offers several potential advantages when compared to modern medical therapy as it emphasizes a holistic approach with minimal adverse effects^40,52^ when practiced correctly after proper guidance. It is also known to improve neuropsychiatric conditions^53^ without the side effects of pharmaceutical drugs. More importantly, it promotes self-empowerment as it empowers individuals to take an active role in their health. Lastly, Qigong is more cost- effective compared to certain medical treatments as once learned, it can be practiced regularly to promote a healthy lifestyle without the need to incur additional expenses.

Among the many styles of Qigong is Guolin Qigong (GQ). This form of Qigong has been specifically adapted for cancer survivors by offering a moderate and adaptable exercise routine. It is a moderate walking exercise that integrates coordinated arm movements and trunk rotations, coupled with controlled breathing techniques. What sets it apart is its gentle nature, designed to be accommodating and adjustable. This adaptability is crucial, as it allows for modifications in speed, duration, and intensity, ensuring that it caters to the unique health conditions and needs of individual cancer survivors. The flexible approach of GQ prioritizes the well-being and comfort of each participant, making it a safe and beneficial practice for those navigating the complexities of cancer recovery. Therefore GQ is suitable for cancer survivors—especially advanced cancer patients who may be unable to engage in other forms of physical activity.^54^ A moderate-intensity walking exercise program was reported to be a feasible and effective intervention method for managing fatigue,^55,56^ anxiety, depression,^57^ and sleep in cancer survivors at different stages of cancer.^58-60^ GQ exercise training also facilitates the inhaling of oxygen which is important for the homeostasis of oxygen-carbon dioxide in controlling cancer and ultimately survival, as supported by a 10-year longitudinal cohort study of 122 lung and nasopharyngeal cancer patients which reported that improvements in End Tidal Breath Holding Time (ETBHT), alveolar O_2_ (aO_2_) pressure, and alveolar CO_2_ (aCO_2_) pressure capacity with GQ was associated with higher survival years, 5 years survival rate and survival probability.^61^ In the above-mentioned cohort study,^61^ GQ also demonstrated improvement in depression among cancer participants. Additionally, in breast cancer patients undergoing 5 to 6 weeks of radiotherapy, women with elevated depressive symptoms at the start of treatment showed significant enhancements in fatigue and QOL.^50^

However, the impact of GQ on CRF, often associated with sleep and depression^3,26-28^ and its correlation with cortisol levels has not been explored.

In addition to that, a qualitative study to evaluate the perception and acceptance of GQ is needed to explore the feasibility, adherence, and experience related to this complex intervention. It aims to understand the more subjective domains and identify potential factors for improving healthcare^62^ to address the multi-faceted needs of this population. Such research, however, has not been conducted yet.

Therefore, the purpose of this paper is to describe the protocol of a mixed-method study comprising,

1. A randomized-controlled trial to evaluate the feasibility of GQ training and its effect on CRF, sleep, depression, and cortisol levels in breast, lung and colon cancer survivors and,
2. Semi-structured interview with study participants from the randomized- controlled trial to understand their perceptions and experiences of GQ to identify:
  (a) other physiological and psychological benefits gained from the GQ intervention,
  (b) the barriers to GQ participation, adherence, and recommendations for improvement.

We hypothesized that

1. It is feasible for cancer survivor participants to engage in the GQ training and those who participated in the GQ training will experience positive impacts on CRF, sleep, depression, and cortisol level. Feasibility of this study is established at about 80% to 85% retention rate and 75% to 85% class attendance rate based on prior similar studies.^63-65^
2. The semi-structured interview will identify a broader spectrum of benefits and barriers to participation and adherence to GQ training to provide valuable insights for program enhancement.

## Methods

### Study Design

#### Intervention/clinical trial

**Figure 1.**
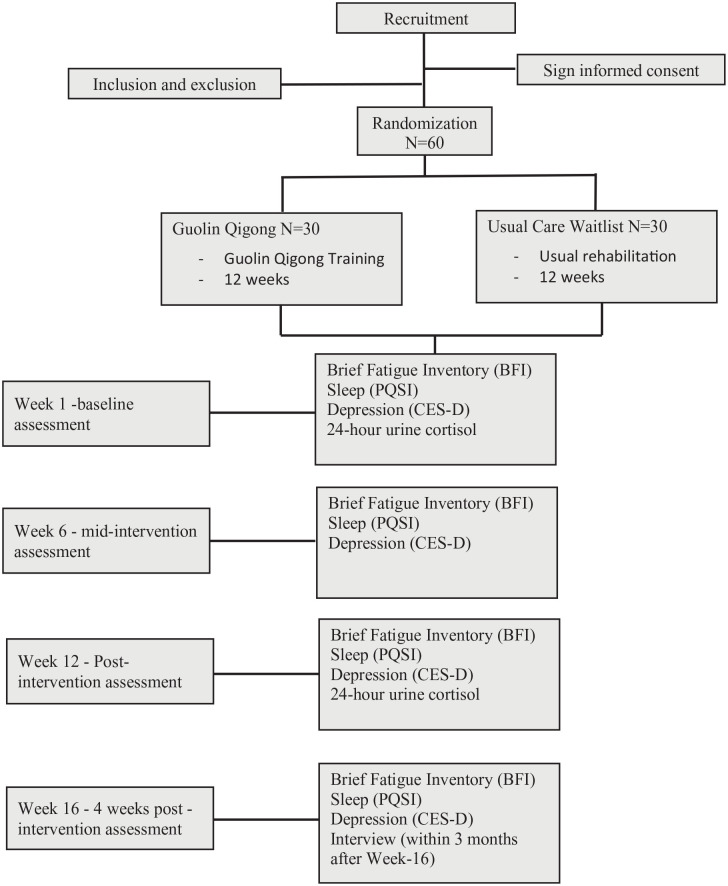
Participant flow diagram.

The primary objective of this study is to explore the feasibility of a GQ intervention in patients with various cancer types who experience CRF. This study includes the 3 most prevalent cancer types that is, breast, lung and colon cancers. This is a randomized-controlled trial that compares the GQ intervention group to a usual care waitlist control group receiving standard medical care allocated in a 1:1 ratio to evaluate the feasibility and effects of a 12-week GQ intervention on 60 eligible breast, lung, and colorectal cancer survivors’ fatigue, sleep quality, depression, and 24-hour urine cortisol change. Refer to Figure 1 for an overview of the study. This study protocol was developed based on the SPIRIT 2013 Statement (Standard Protocol Items: Recommendations for Interventional Trials) (Supplemental Material 1).^66^

#### Interview

We will use a qualitative descriptive design, with a semi-structured interview to collect in-depth information to enhance our understanding of the perceptions and experiences of GQ participation of cancer survivors based on purposive sampling until data saturation is reached.

The qualitative interview will be reported within the primary randomized feasibility trial according to the guidelines recommended in the CONSORT 2010 statement extension.^67^

### Study Setting

The trial is conducted at University Malaya Medical Centre (UMMC) in Kuala Lumpur, Malaysia. Participants are recruited by referrals from UMMC or online and print advertisements such as posting recruitment in UMMC, cancer support groups, GQ Association and GQ practice stations around Klang Valley, and social media platforms including X Corp (formerly known as Twitter) and Facebook.

### Recruitment

Recruitment started in October 2022. Upon first contact, the Researcher will screen potential breast, lung, or colon cancer survivors and provide information about the study to those who meet the eligibility criteria and indicate interest to participate. Thereafter a Physician/Oncologist Screening Form, Participant Information Sheet, and 2 copies of Participant Consent Form will be given, explained, and a separate date agreed for in-person enrollment.^67^

### Inclusion Criteria

(1) Age ≥ 18 years old;
(2) *Cancer survivor diagnosed with breast, lung and colorectal cancer;
(3) Able to read and answer questionnaires in English and/or Bahasa Malaysia language by oneself;
(4) Suffering from moderate to severe fatigue as assessed by the simple fatigue scale; with severity score of 3 and above^68^;
(5) Able to use smart phones and the WeChat or WhatsApp application;
(6) Life expectancy of more than 3 months;
(7) Able to give informed consent;
(8) Available and willing to comply with the study requirements.

*A cancer survivor is defined as an individual diagnosed with cancer through the balance of life according to National Cancer Institute.^69^

Including cancer survivors throughout the continuum of survivorship in our sample inclusion criteria allows us to capture a comprehensive spectrum of experiences. From those newly diagnosed to long-term survivors, each stage brings unique perspectives on GQ participation and its impact. This approach ensures a thorough exploration of how GQ intervention intersects with varying phases of the cancer journey, providing a rich understanding of the diverse narratives and challenges encountered across this continuum.

### Exclusion Criteria

(1) Cardiopulmonary disease, nerve, muscle, or joint disease, or other malignant tumors affecting movement;
(2) Mental illness or serious cognitive impairment and defects in language that significantly impairs communication;
(3) Post-operative heart, cerebral vessel, or other serious complications;
(4) Neurological degenerative disease (e.g. dementia), reduced cognitive; capacity in any way that would affect ability to understand trial procedures and give informed consent;
(5) Patients who are not able to walk;
(6) Other medical conditions which would preclude study intervention or make study participation unsafe such as severe chronic heart failure.

### Randomization and Blinding

After informed consent, participants will be randomly assigned to either the GQ or usual care waitlist control group at a 1:1 ratio with the random sequence generated by an online computerized randomization system using minimization method^70-72^ by an independent research assistant.

### Intervention

After being advised of group allocation, the study participant will be given a few days to decide on whether to proceed with enrollment. Incorporating a brief period for participants to reflect and opt-out after randomization supports ethical research practices, ensures ethical informed consent, reduces pressure, enhances retention, and respects participant autonomy in clinical trials or studies.

#### Guolin Qigong

Participants in the intervention group will attend a 2-hour supervised face-to-face (F2F) GQ training class for 12 weeks with home-practice on non-class days. There will be 2 training classes per week for the first 2 weeks and once per week for the next 10 weeks.

The GQ training involves a modified version of a Chinese medical qigong therapy often referred to as “walking qigong” developed by Guo Lin.^38^ GQ was developed specifically for cancer and consisted of several breathing and moving exercises. The GQ training in the current study includes the following 3 main exercises:

1. Feng Hu Xi Zhi Ran Xing Gong (Natural Wind Breathing Walking)

Participants walk in a circle, synchronizing their breathing, arm movements, and steps focusing on the movement of their body with the goal of calming one’s mind, relaxing various parts of the body and the mind, and revitalizing the “life-force,” that is, *qi*.

2. Dian Bu Gong (Step Tap Method)

A modified natural Wind Breathing Walking Exercise with additional step tap.

3. Sheng Jiang Kai He Fa (Ascending, Descending, Opening, and Closing Method)

A calming and relaxation method of synchronized arms and legs movements on the spot which can be practiced within limited space.

Each main exercise has to be practiced separately as individual sets comprising 3 parts:

a. Preparatory relaxation exercise (gentle breathing and meditation; 4 minutes):

In relaxed standing posture, synchronize the breath with gentle arm movements in front of the abdomen (opening and closing of the Dantian). Dantian is a Chinese medicine concept that refers to the “elixir field” or “energy center.” It has different locations but often referred to the lower abdomen between the navel and pubic bone of the human body.^73,74^

b. Main exercise
i. Feng Hu Xi Zhi Ran Xing Gong (Natural Wind Breathing Walking) or
ii. Dian Bu Gong (Step Tap Method) or
iii. Sheng Jiang Kai He Fa (Ascending, Descending, Opening, and Closing Method).
c. Concluding exercise: breathing exercises, opening, and closing of the Dantian, and self-massage (3-6 minutes).^50^

Please refer to Supplemental Material 2 for detailed steps. All movements will include modifications to enhance safety. Participants will be advised to rest as and when necessary, according to their health status.

The F2F GQ class will be taught by qualified GQ instructors who are both long-term cancer survivors with more than 20 years of GQ training experience to ensure the full potential of the GQ intervention. The Researcher, who is a registered Chinese Medicine practitioner in Malaysia and Australia with more than 20 years’ experience in Qigong, will be in attendance during the F2F GQ class to ensure the safe and seamless facilitation of the training session.

Home practice is recommended for non-class days according to individual health status and available time.^54^ Participants will be provided with class notes and video links (Supplemental Material 3) to promote and support home practice. Home practice after the intervention period is encouraged for incorporation into daily life to foster a healthy lifestyle.

### Usual Care Waitlist Control

Participants in the usual care waitlist control group will be advised to continue with their usual care routines and exercises and will be asked not to attend any other Qigong exercises. They will be offered to attend the same GQ training class after Week-16 of the GQ training of their cohort.

However, to maintain integrity of the study, instructors will not be informed about the allocation of participants. Consequently, when instructors commence training of subsequent cohorts, they will be unaware of which participants belong to the intervention group (commencing from Week 1), and which are part of the control group (joining the GQ group in Week 1), given the GQ training will be offered continuously. This ensures the instructors are effectively blinded to the allocation to maintain impartiality in the delivery of the training and minimizes any potential biases that may arise from instructor knowledge of group allocation.

### Study Assessments

The variables under consideration will encompass basic demographic data, primary outcomes, and secondary outcomes. Basic characteristics will be collected at baseline (Week-1).

**Figure 2.**
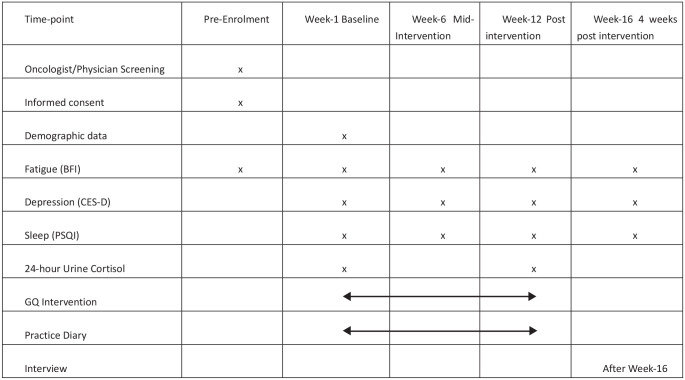
Schedule of enrollment, intervention and data collection Abbreviations: BFI, Brief Fatigue Inventory, CES-D, Centre for Epidemiologic Studies Depression; PSQI, Pittsburgh Sleep Quality Index.

Primary feasibility outcomes on adherence, attendance, and any adverse events will be recorded throughout the intervention period from Week-1 to Week-12 in the practice diary by the participants. Secondary patient-reported outcomes on fatigue, depression, and sleep will be measured at baseline (Week-1), mid-intervention (Week-6), post-intervention (Week-12), and 4 weeks after completion of intervention (Week-16). Urine samples (for urine cortisol test) to assess 24-hour urinary cortisol will be collected a day prior to enrollment date and post-intervention (Week-12). Primary feasibility outcomes and secondary patient-reported outcomes will be assessed by an independent research assistant to minimize bias. A summary of the planned data collection is shown in Figure 2.

#### Basic demographic data

Participants’ basic characteristics (sex, marital status, age) and cancer history will be collected using a self-designed questionnaire.

#### Feasibility outcomes

Feasibility will be assessed based on recruitment rate, retention rate, class attendance, home practice adherence, the nature and extent of missing data, and safety. Safety will be monitored and assessed based on total number of Adverse Event and the presence of any Serious Adverse Event.^75^

#### Patient-reported outcomes and cortisol biomarker

i. Patient-reported outcomes

Participants will complete several patient-reported outcome measures to evaluate fatigue—Brief Fatigue Inventory (BFI),^68^ sleep quality—Pittsburgh Sleep Quality Index (PSQI)^76^ and depression—Centre for Epidemiologic Studies Depression (CES-D)^77,78^ at Week-1, Week-6, Week-12, and Week-16.

a. Fatigue—The Brief Fatigue Inventory (BFI)

The BFI was developed for the rapid assessment of fatigue severity in cancer patients for use in both clinical screening and clinical trials. It is easy to administer, and patients can complete them easily as it has only 9 items with simple wordings that are easy to understand and translate.^68^ Items 1 to 3 rate participants’ worst, usual, and current fatigue during the past 24 hours using a visual analog scale (VAS) ranging from 0 (no fatigue) to 10 (very severely fatigued). A composite fatigue severity score was obtained by averaging the 3 severity scores. The remaining 6 items rate the level at which patients` fatigue interferes with certain functions such as daily activity, work, walking ability, normal work, relations with others, and enjoyment in life. Each item is rated on a scale of 0 (does not interfere) to 10 (completely interferes). A composite fatigue interference score will be obtained by averaging the scores of the 6 interference items. Severity scores of 1 to 3 suggest mild fatigue, 4 to 6 moderate, and 7 to 10 represent severe fatigue. The internal consistency Cronbach’s α at .96 supports the reliability of BFI which is correlated with measures of performance status (patients who are more ill reported higher levels of fatigue).^68^

b. Sleep—Pittsburgh Sleep Quality Index (PSQI)

The PSQI, proposed by Buysse et al^76^ in 1989, is currently the most commonly used scale for assessing sleep quality. The PSQI is a 19-item scale measuring 7 aspects of the sleep quality and disturbances, that is, subjective sleep quality, sleep latency, duration, efficiency, disturbances, use of sleep medication, and daytime sleep dysfunction, each weighted equally on a 0 to 3 scale. The total score ranges between 0 and 21, with increasing scores indicating worse sleep quality.^76^ It has a sensitivity of 89.6% and a specificity of 86.5% to detect insomnia cases and is a useful first-line, easy-to-handle, and time-efficient questionnaire to evaluate sleep quality.^79^ The PSQI was reported to be a stable measure of sleep quality with high test-retest reliability and construct validity.^79,80^

c. Depression—Centre for Epidemiologic Studies Depression (CES-D)

The CES-D is a short self-reported 20-item scale^81^ to be administered to measure depression symptoms at baseline, mid-intervention, post-intervention, and follow-up. The CES-D shows very high internal consistency and adequate test-retest repeatability.^81^ The score is the sum of all items, and the maximum score is 60. Scores ≥ 16 on the CES-D is considered as having “depressed symptoms.” The cut off score of 12 has been validated with DSM-III criteria for clinical depression.^81,82^

ii. Cortisol biomarker: 24-hour urine cortisol

Urine sample will be collected at baseline (Week-1) and post-intervention (Week- 12).^83^ Participants are briefed on the urine collection procedure during assessment and advised to obtain the 2.5 L urine collection container from any nearby BP Laboratories before baseline (Week-1) and post-intervention (Week-12). We use 24-hour urinary cortisol as a non-invasive, integrated measure of cortisol stress hormone activity that could be collected in participants’ home environments. Participants are instructed to collect all urine for 24 hours from 8:00am a day prior to baseline (Week-1) and post-intervention (Week-12) to 8:00am the next mornings and to send the urine sample collected to the nearest BP Laboratories the next morning itself. If the 2.5L collection container is completely full, subjects are advised to use a clean mineral water bottle to collect any specimen in excess of the 2.5 L collection. If subjects are unable to collect all urine for any reason, their samples will be deemed inadequate and no urinary cortisol data will be recorded for these subjects.

The 24-hour urine cortisol test provides a more accurate estimate of overall cortisol secretion^84^ to assess urinary-free cortisol (UFC) over a 24-hour period and provides an index of (unbound serum cortisol) the total amount of cortisol released by the adrenals. The 24-hour collection time reflects the amount of cortisol that is released over a complete circadian cycle, which is a physiologically meaningful unit of time. An integrated mean of cortisol levels over a 24-hour period is thought to provide a moderately stable measure of adrenocortical output.^85^ Urinary cortisol output will be analyzed by chemiluminescent microparticle immunoassay (CMIA) at BP Laboratories, headquartered in Shah Alam, Selangor Darul Ehsan, Malaysia.

#### Qualitative interview

Semi-structured interviews will be conducted to inform interpretation of quantitative results and participants’ acceptance, experience, and subjective perception of the study as well as to find out areas for further improvement^86^ after Week-16.

A prepared interview guide (Supplemental Material 4) is designed to delve into participants’ experiences and will be used in the interview to cover 3 primary themes: the experiences of practicing GQ, changes in the physical and emotional state, and suitability of GQ for cancer survivors. Examples of questions to be asked are “How do you feel about the 12 weeks of Guolin Qigong study?,” “Have you had other benefits from this Guolin Qigong study?,” and “Would you recommend Guolin Qigong to other cancer survivors? Why? Why not?.”

Each interview will take approximately 45 minutes and will be audio-recorded and transcribed verbatim by the Researcher.

## Protocol Amendments

The Researcher is responsible for submitting materials to the human ethics committee.

### Trial Monitoring

The Researcher will report progress of the trial to the panel of supervisors on a quarterly basis. Annual reports will be submitted to the human ethics committee to monitor the trial progress and outcomes. Participants in the GQ group will be advised to record any perceived adverse events in the practice diary and this will be recorded in participants’ Adverse Event Form by the Researcher. The severity, expectedness and causal relationship with GQ intervention will be assessed for every adverse event according to guidelines for safety reporting.^75,87^

The schedule of the enrollment, intervention, and data collection is shown in Figure 2.

### Sample Size

Using a small effect size (0.2)^65^ rather than a medium effect size (0.5)^64^ to achieve a more conservative estimation, the sample size is 25 cases in each group with 90% power and two-sided 5% significance trial design.^88^ Allowing for a drop-out of about 20% based on previous studies,^89-92^ a total sample of 60 (30 in each group) would be required.

### Data Collection

De-identified information will be collected and managed by assigning an identification code to each participant. Demographic information, medical history, any occurrence of adverse events, practice adherence and intervention outcomes during the study will be collected and recorded on paper or electronic file.

Demographic information and medical history will first be collected by the Researcher. De-identified Practice Diary, Brief Fatigue Index, Pittsburgh Sleep Quality Index, and Centre for Epidemiologic Studies Depression will be collected at Week-1, Week-6, Week-12, and Week-16, while the 24-hour urine cortisol results will be collected by the research assistant at Week-1 and Week-12. Interviews will be conducted by the Researcher after Week-16, audio-recorded, de-identified, and transcribed verbatim.

During the study, all data will be recorded in participant source document files in paper format (sociodemographic data, feasibility and clinical data), administered by independent research assistants and will be transferred to an electronic format for storage and analysis. The 2 independent research assistants will verify and check the data collected by each other. After all data are confirmed and checked the research assistants will extract the data into an Excel sheet for analysis.

After participants have been randomized, dropout and premature termination or withdrawal from the 2 groups at any point will be recorded along with relevant reasons. Participants can choose to withdraw and will not be affected at any time while declining to participate or withdrawing from the trial. In some cases, participants will discontinue the intervention, such as illness, progression of their disease, or inability to engage despite adjustment of the program. Participants who withdraw from the intervention during the trial will not be replaced.

Interviews will be conducted after Week-16 in person or online via Zoom by the Researcher at an appointed time. Interview sessions will be audio recorded and transcribed verbatim using NVivo or the recording feature available in Zoom, ensuring accurate documentation, and stored in electronic files for analysis.

### Data Management

De-identified electronic data will be stored on a password-protected computer. Files will be accessible for review only to the research team. Specific requests will be needed from the supervisory panel for any access by any other person. All completed forms and study information will be de-identified and stored in a locked filing cabinet together with consent forms at UMMC.

The information collected will be securely locked and only accessible to the research personnel. The data will be de-identified for analysis and once date is coded, all identifiable information will be destroyed. All data collected in the study will be destroyed 7 years after publication.

## Data Analysis

### Quantitative Data

Data analysis will be conducted by an independent statistician. Data analysis will be by intention to treat using last observation carried forward (LOCF).^93^ The normality of the study data will be checked by examining the mean, median, skewness, and kurtosis including graphical representation of the box plot, normal Q-Q Plot or histogram. Continuous data will be summarized using descriptive statistics including the number of observations used in the calculation (*n*), mean, standard deviation (SD), minimum, median and maximum. Categorical data will be summarized as counts and percentages of each category. Between group comparison will be conducted using the t test, chi-square test, or Fisher exact test, as appropriate. We will report the results for two-sided 5% tests for the primary trial outcome. We will use generalized linear mixed model (GLMM). The outcomes of the model will be BFI, PSQI, CES-D scores, and 24-hour urine cortisol test results. Models will include the treatment group as a fixed effect, with time as a random effect to account for the repeated measurements. Analyses will be conducted to identify the baseline characteristics of the participants who may benefit most from the intervention. Model assumptions will be checked and appropriate adjustments to the analysis will be made where necessary. STATA^®^ software version 17.1 (2021; Stata Corporation, College Station, TX, USA) will be used for all analyses. Cohen’s D will be calculated as the absolute differences between the mean change in the variable divided by pooled variance of the change.

### Qualitative Data

The researcher will conduct an interview consisting of 6 pre-determined open-ended questions (Refer Supplemental Material 4) that were identified from literature review and based on research questions outlined in the study. Probing questions will only be asked where clarification or expansion of expressed ideas is required. Interviews will be conducted until data saturation is reached. Interview transcripts will be analyzed using the inductive qualitative content analysis approach to provide a comprehensive descriptive summary of the interviewee’s experience and perception of GQ in everyday terms.^94^ All audio recordings of the interview will be transcribed verbatim with NVivo or Zoom. First, to obtain an overall impression, the transcribed materials will be read several times thoroughly. Then, sentences or phrases will be condensed and divided into ideas/meaning units. The ideas/meaning units are further condensed, abstracted, and conceived into the categories. All categories will be put together and compared, based on differences and similarities and group together to create themes. This iterative approach to analysis will be conducted until no new ideas/codes emerge and saturation is achieved. A second reviewer will review each phase of the process. A summary of each theme and supporting quotes from participants will be generated and shared with the study team to review independently. Data analysis will be repeated in this manner until a final consensus is reached on final themes to ensure validity.^95,96^ The Consolidated Criteria for Reporting Qualitative Research (COREQ)^62^ will be used for reporting the findings.

### Trial Registration and Ethical Issues

This study has been registered with ANZCTR (ACTRN12622000688785p), was approved by Medical Research Ethic Committee of University Malaya Medical Centre (MREC ID NO: 2022323-11092) and recognized by Western Sydney University Human Research Ethics Committee (RH15124).

### Dissemination

The results of this study will be reported in a PhD thesis by the first author and submitted for publication following the rules of CONSORT in a peer-reviewed journal or presentation using de-identified or non-identifiable information. Sensitive information including participants’ details will not be disclosed and will be treated strictly under the privacy and confidentiality act of Malaysia and Australia.

## Discussion

CRF is endemic in cancer survivors with disabling effects on quality of life, co-morbidities, independent living, and work productivity. It includes physiological changes associated with cancer diagnosis and treatment such as cortisol dysregulation, sleep disorders, depression, and functional disability, which further contribute to the burden of CRF. Despite the high incidence of CRF and its symptom clusters involving sleep quality and depression, there is no effective pharmacological intervention to prevent or alleviate these symptoms,^68^ highlighting the need to study CRF and its related symptom clusters to understand the common biological mechanisms to improve quality of life of cancer survivors. Numerous studies have reported certain psychological and physiological benefits of Qigong for cancer survivors, promising potential in the realm of cancer medicine. While its definitive impact requires further extensive research, some studies suggest benefits such as enhanced physical functioning, stress reduction, improved mental health, immune enhancement, and improved quality of life, and that it may complement conventional medicine as adjunct therapy by providing a holistic approach to cancer care.^29,33-44^ As research progresses, understanding its potential role in cancer medicine could open doors to more comprehensive and patient-centric approaches to healthcare. In this regard, GQ emerges as a unique mind-body exercise that combines moderate and adaptive walking exercise with a pronounced focus on a specialized breathing technique.^97^ While walking has been reported to improve cancer-related symptoms,^57-60^ the incorporation of breathing technique of GQ serves to foster oxygen-carbon dioxide homeostasis—an essential factor in cancer control and ultimately survival.^60,61^ As such GQ stands as a promising avenue for addressing the cluster of symptoms associated with CRF, encompassing sleep disorder, depression, and alterations in cortisol. However, despite this promising potential, the comprehensive exploration of GQ’s benefits remains a yet untapped opportunity.

Therefore, our hypothesis posits that the current study of GQ is a feasible intervention that holds potential to ameliorate CRF, enhance sleep quality, alleviate depression, and regulate cortisol levels among breast, lung, and colon cancer survivors, while concurrently unveiling a comprehensive spectrum of benefits and barriers related to participation and adherence to GQ training, offering invaluable insights for optimizing and enriching the program.

This is the first mixed-method study comprising a randomized-controlled trial and semi structured interview to evaluate the feasibility and benefits of GQ on CRF symptom cluster among cancer survivors. The quantitative data from the randomized-controlled trial complements the qualitative insights to be obtained through interviews, allowing for a comprehensive exploration of the subjective domains of feasibility, adherence, barriers and participants’ perceptions, experiences of GQ to help improve healthcare and identify factors for enhancing GQ intervention effectiveness.

Moreover, the composition of this multidisciplinary study team comprising experts from Western Medicine and Chinese Medicine integrating their expertise from different perspectives will lead to robust study design, enhanced participant care, rigorous data analysis and comprehensive interpretation of results.

## Limitations

The nature of the intervention precludes blinding of both participants and researcher. While the researcher remains unblinded as this trial is open-label, the instructors will not be aware of intervention allocation to minimize bias. Although including cancer survivors across all stages enables a diverse assessment of feasibility, it potentially limits the generalizability of the results though sub-group analysis will be conducted. Furthermore, the reliance on self-reported data for secondary outcomes emphasizes the necessity for incorporating objective measures like actigraphy, polysomnography, and established scales such as the Hamilton Depression Rating Scale to ensure a comprehensive evaluation of GQ’s impact on CRF, sleep quality, and depression. Additionally, the predominant participation of Asian Chinese individuals highlights the need for expanded research to comprehend GQ’s feasibility and effects among cancer survivors from various ethnic and racial backgrounds. These limitations underscore the challenges inherent in the study’s design and demographics, emphasizing the importance of integrating objective measures and broadening participant diversity for more conclusive and inclusive findings.

Despite the limitations, exploring GQ as a potential intervention to address existing gaps in effective treatment for CRF and its related symptom cluster of sleep quality and depression in cancer survivors demonstrates a forward-thinking approach in the field of oncology. It expands on the knowledge base and promotes innovative thinking, fostering the development of novel interventions with potential contribution to the advancement of oncology care.

## Conclusion

The insights gained from this study may guide future design and implementation of evidence based mind-body interventions to improve cancer survivorship.

## Supplemental Material

sj-docx-2-ict-10.1177_15347354241252698 – Supplemental material for Exploring Guolin Qigong (Mind-Body Exercise) for Improving Cancer Related Fatigue in Cancer Survivors: A Mixed Method Randomized Controlled Trial Protocol Supplemental material, sj-docx-2-ict-10.1177_15347354241252698 for Exploring Guolin Qigong (Mind-Body Exercise) for Improving Cancer Related Fatigue in Cancer Survivors: A Mixed Method Randomized Controlled Trial Protocol by Sara L. K. Low, Gwo Fuang Ho, Bingkai Liu, Eng-Siew Koh, Yutong Fei, Chiah Shean Teo and Xiaoshu Zhu in Integrative Cancer Therapies

sj-docx-3-ict-10.1177_15347354241252698 – Supplemental material for Exploring Guolin Qigong (Mind-Body Exercise) for Improving Cancer Related Fatigue in Cancer Survivors: A Mixed Method Randomized Controlled Trial Protocol Supplemental material, sj-docx-3-ict-10.1177_15347354241252698 for Exploring Guolin Qigong (Mind-Body Exercise) for Improving Cancer Related Fatigue in Cancer Survivors: A Mixed Method Randomized Controlled Trial Protocol by Sara L. K. Low, Gwo Fuang Ho, Bingkai Liu, Eng-Siew Koh, Yutong Fei, Chiah Shean Teo and Xiaoshu Zhu in Integrative Cancer Therapies

sj-docx-4-ict-10.1177_15347354241252698 – Supplemental material for Exploring Guolin Qigong (Mind-Body Exercise) for Improving Cancer Related Fatigue in Cancer Survivors: A Mixed Method Randomized Controlled Trial Protocol Supplemental material, sj-docx-4-ict-10.1177_15347354241252698 for Exploring Guolin Qigong (Mind-Body Exercise) for Improving Cancer Related Fatigue in Cancer Survivors: A Mixed Method Randomized Controlled Trial Protocol by Sara L. K. Low, Gwo Fuang Ho, Bingkai Liu, Eng-Siew Koh, Yutong Fei, Chiah Shean Teo and Xiaoshu Zhu in Integrative Cancer Therapies

sj-pdf-1-ict-10.1177_15347354241252698 – Supplemental material for Exploring Guolin Qigong (Mind-Body Exercise) for Improving Cancer Related Fatigue in Cancer Survivors: A Mixed Method Randomized Controlled Trial Protocol Supplemental material, sj-pdf-1-ict-10.1177_15347354241252698 for Exploring Guolin Qigong (Mind-Body Exercise) for Improving Cancer Related Fatigue in Cancer Survivors: A Mixed Method Randomized Controlled Trial Protocol by Sara L. K. Low, Gwo Fuang Ho, Bingkai Liu, Eng-Siew Koh, Yutong Fei, Chiah Shean Teo and Xiaoshu Zhu in Integrative Cancer Therapies
