# Supplementary material for: Exploring Guolin Qigong (Mind-Body Exercise) for Improving Cancer Related Fatigue in Cancer Survivors: A Mixed Method Randomized Controlled Trial Protocol
Source: Integr Cancer Ther. 2024 May 17;23:15347354241252698. doi: 10.1177/15347354241252698 (PMC11102686; doi:10.1177/15347354241252698)
Supplement: sj-docx-2-ict-10.1177_15347354241252698 – Supplemental material for Exploring Guolin Qigong (Mind-Body Exercise) for Improving Cancer Related Fatigue in Cancer Survivors: A Mixed Method Randomized Controlled Trial Protocol [file sj-docx-2-ict-10.1177_15347354241252698.docx]

**Supplementary Material 2 Detailed Guolin Qigong Training Steps**

This section contains the instruction for the application of the three Guolin Qigong methods i.e. Feng Fu Xi Zhi Ran Xing Gong, Dian Bu Gong and Sheng Jiang Kai He Fa used in this study. The three methods used are some of the methods of Guolin Qigong and they are based on the Guolin New Qigong Practice Manual.^1^

1. **Feng Hu Xi Zhi Ran Xing Gong (Natural Wind Breathing Walking Exercise)**

This is the primary walking exercise of Guolin Qigong. It can be practised at a speed you use for normal walking. The breathing method used while practising this exercise is «moderate (medium strength) Wind breathing" (中度风呼吸), a cycle of two inhalations and one exhalation is completed for every two steps. This exercise can increase your intake of oxygen, invigorate the inner qi, free the blockages in the meridians, regulate qi and blood, improve circulation, balance the yin and yang in the body and improve immunity. So, it can be an effective treatment of various diseases including influenza, inflammatory diseases, mild fever and cancer, and improve our immunity against these diseases.

- 1. **Essentials**

1. **Raise your toes, point your feet straight ahead, keep your knees slightly bent**

When stepping forward, raise your toes naturally. But do not overdo which can cause spasm. Lower your heel to the ground first and keep your knees slightly bent. Walk in the Relaxed Standing posture straight ahead like along two straight lines.

Raising your toes and lowering the heel first stimulate the kidney meridian, strengthen your kidneys and solidify the foundation for your health.

Walking with your feet pointing straight ahead (not pointing outward or inward) is to invigorate your Yin Heel and Yang Heel Vessels. When these two vessels are active, the yin and yang in the body are kept in balance. The Yin Heel Vessels originate on the inside of the heel and the Yang Heel Vessels on the outside of the heel, so if you walk incorrectly with your feet pointing outward, only the Yang Heel Vessels are stimulated and a balance cannot be achieved.

When you keep the knees slightly bent, your rounded posture allows gi to flow freely along the meridians on your legs. You need to do this when you practise all the exercises of Guolin Qigong, not only Natural Walking exercise. However, you should bend your knees naturally and not too much because this causes tension.

1. **Lower your hips a little and relax your waist**

When you practise Guolin QiGong, you should not think about Middle DanTian and should not use your mind to lead the flow of qi. To activate your inner qi, you must be relaxed and be natural. Master Guo Lin emphasized the importance of relaxing your waist in order to enable the qi to gather in the Middle Dan Tian. Lowering your hips a little is to relax your waist. The best way is to exhale a long breath, your height will then be a little lower than your normal height and keep this posture while you practise. However, you must not lower too much like squatting because you cannot walk naturally by doing so.

1. **Guide the qi into Middle DanTian**

When you move your hands toward Middle DanTian, qi in your hands can be guided into Middle DanTian. When there is an abundant amount of qi in Middle DanTian, qi can be distributed to nourish the whole body. We can then realize the benefits of practising qi gong.

When practising this exercise, you move your hand between HuanTiao (环跳), a point at the side of your hip on the Gallbladder Meridian (a yang meridian), and Middle DanTian (QiHai 气海, literally sea of qi), a point about 1.5 cun (2-fingers width) below the navel on the Conception Vessel (a yin channel). Moving the hands between these two points can invigorate the qi flow in your three Yin and three Yang Meridians of Hand, and help balance the yin and yang in your body.

1. **Turn your head naturally.**

When you practise Natural Walking exercise, you need to turn your waist and head naturally. This can stimulate the six yang meridians which run along both sides of the back of your neck. Turning your waist turns the spine which stimulates the acupressure points on both sides of the spine associated with your internal organs. Thus, the qi of your organs is invigorated and regulated, blockages in the meridians are removed and circulation is improved. The functions of your organs are then enhanced.

If you do the above four essentials properly, you can then realize the benefits of practising the exercise. Other than the above, the following points are also important:

1. ﻿﻿﻿ Motion and Stillness

The theory of Guolin Qigong emphasizes that other than motion, stillness is also an important element of the exercises. When you practise, you are not moving constantly, there must be a short pause ("moment of stillness") between movements.

1. ﻿﻿﻿ Regulate Yin and Yang

When you move your hands between Middle Dan Tian and the side of your hips, the hand at Middle Dan Tian must be at a higher position than the other hand. Higher is yang, lower is yin, inside is yin and outside is yang. So, moving your hands from an inside and higher position (at front of Middle DanTian) to an outside and lower position (at the side of your hips), yin and yang in your body can be balanced.

3. Sway your spine

When you practise the exercise, keep your spine loose and not with a stiff or straight back, have a little forward and backward as well as a left and right swaying motion.

**1.2 Method**

**1.2.1 Which Leg to Start**

For people with liver, gallbladder or eye disease, start the exercise by stepping forward with their right foot first.

For people with heart, small intestine and brain disease, start the exercise by stepping forward with their left foot first.

Otherwise, men start the exercise by stepping forward left foot first, and women start the exercise by stepping forward right foot first.

**1.2.2 Palm direction**

Practise with your palm facing the direction appropriate to your health conditions:

- 'Reducing' method - this method is suitable for most cancer patients. Move your hands between the front of your Middle DanTian and the side of your hip with the palms facing down and fingers pointing forward.
- ﻿﻿'Tonifying method - this method is suitable for most patients with chronic disease and healthy people. Move your hands between the front of your Middle DanTian and the side of your hip with the palms facing DanTian and your body (Figure 1-1a).
- ﻿﻿'Adjusting' method - Move your hands from the front of your Middle DanTian to the side of your hip with the palm facing down, and from the side of your hip to the front of your Middle Dan Tian with the palm facing DanTian (Figure 1-1b). It is suitable for cancer patients who are feeling weak after practising the reducing method for some time. However, you cannot keep using this method, you should switch back to the reducing method after some time.
- 'Lowering' method - Move your hands between the front of your Middle DanTian and the side of your hip with the palms facing DanTian and your body, and fingers pointing down (Figure 1-1c). This is suitable for patients with higher than normal test reading.
- "Raising' method - Move your hands with the palms facing up. This is suitable for patients with lower than normal test reading (Figure 1-1d).


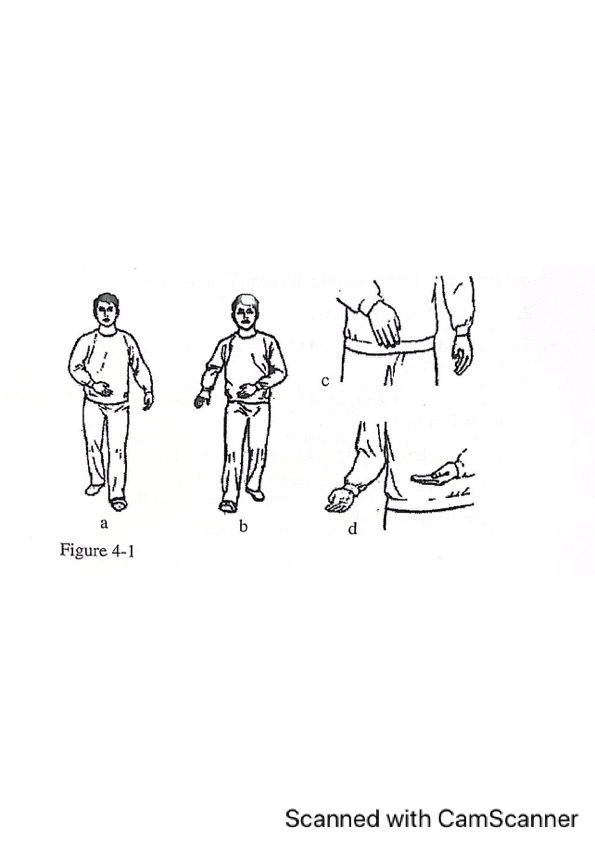


Figure 1-1

**1.2.3 Wind Breathing**

The breathing method used while practising this exercise is moderate (medium strength) Wind breathing (中度风呼吸) in which you use your nose to do two short inhalations (xi xi) followed by one slightly longer exhalation (hu). Medium strength is a strength when the sound of the breathing is just loud enough for you to hear yourself. The volume of air breathed out and duration of the exhalation should equal that of the two inhalations.

People with high blood pressure or heart disease can use natural breathing. After your condition becomes stable, you can switch back to Wind breathing, but the strength should be lighter.

**1.2.4 Exercise sequence**

A practice session of this exercise is divided into two halves. You start the first half of the exercise by stepping forward with your right or left foot first according to your health conditions or gender as described in ‘1.2.1 Which Leg to Start'. After practising for about 20 minutes, stop and do three Opening and Closing at Middle DanTian, then continue to practise the second half by stepping forward with your other foot first. After practising for another 20 minutes, do the Concluding exercise.

The exercise sequence can be summarized as:

Preparatory exercise

- ﻿﻿Start the first half of the exercise by stepping forward with your right or left foot first according to your health conditions or gender.

Practise for about 20 minutes

- ﻿﻿Three rounds of Opening and Closing at Middle DanTian
- ﻿﻿Switch leg to start the second half of the exercise by stepping forward with the other foot first.

Practise for about 20 minutes

- ﻿﻿Concluding exercise

Rest for 10 to 15 minutes

**1.2.5 Ready position**

Get into the ready position as described below for the main exercise:


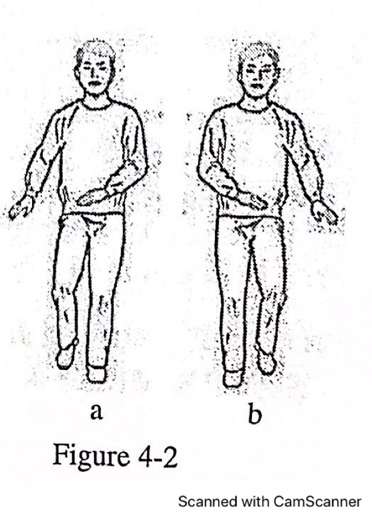
**Start by stepping forward with your right foot**

After the three rounds of Opening and Closing at Middle DanTian (of the Preparatory exercise or after the first half of the exercise), shift your weight to the left foot and position your right foot near the midpoint of your left foot, about 10 cm away, with your right heel up and toes touching the ground. Move your left hand to the front of Middle DanTian and your right hand beside your right hip (Figure 1-2a). Turn your palms to face the appropriate direction as described in 1.2.2 Palm direction'

**Figure1-2a**

**Start by stepping forward with your left foot**

After the three rounds of Opening and Closing at Middle DanTian (of the Preparatory exercise or after the first half of the exercise), shift your weight to the right foot and position your left foot near the midpoint of your right foot, about 10 cm away, with your left heel up and toes touching the ground. Move your right hand to the front of Middle DanTian and your left hand beside your left hip (Figure 1-2b). Turn your palms to face the appropriate direction as described in ‘1.2.2 Palm direction'

**1.2.6 Movements**

**Start by stepping forward with your right foot**

If you are to start the exercise with your right foot according to your health conditions or gender, this is the first half of your exercise.

If you are to start the exercise with your left foot according to your health conditions or gender, this is the second half of your practice. Do your first half of the exercise by following the instructions described in the 'Start by stepping forward with your left foot' section below.

Get into the ready position as described in section "1.2.5 Ready position".

1. Bring your right foot forward a small step with toes raised and pointing straight ahead.

If this is your first step, do not move your hands. Otherwise move both of your hands towards the right while you shift your weight and step forward.

Lower your right heel gently. When the heel touches the ground, your left hand is in front of your Middle DanTian and your right hand by the side of your right hip. At this time, breathe in twice (xi xi) (Figure 1-3a, b).

Lower your right sole gently onto the ground and shift your weight to the front (right leg).


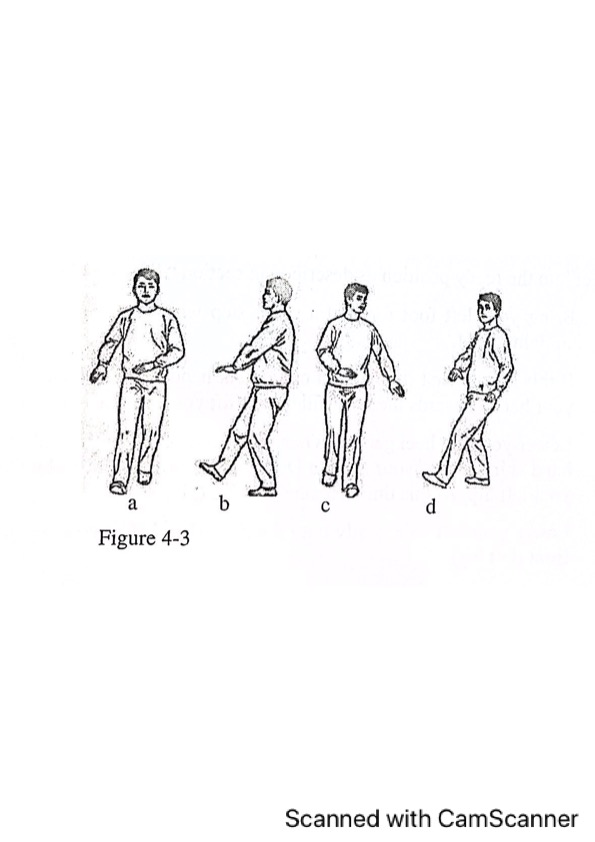


Figure 1-3

2. While shifting your weight forward, move your hands towards the left, step forward your left foot with toes raised and pointing straight ahead. If this is your fourth step or every fourth step thereafter, simultaneously turn your head and your waist towards the left. Lower your left heel gently. When the heel touches the ground, your right hand is in front of your Middle DanTian and your left hand by the side of your left hip. If you have turned your waist and head, your head is at about 60° and your waist at about 45° towards the left (Figure 1-3c, d). At this time, exhale once (hu-).

Lower your left sole gently onto the ground and shift your weight to the front (left leg). If you have turned your waist and head, turn back to front-facing.

You can turn your waist and head every 6 steps if you prefer.

Continue to walk by repeating the above. After walking for about 20 minutes, move up your foot from behind to the Relaxed Standing posture.

If you start the Natural Walking exercise with your right foot, you have completed the first half of the exercise. Do three rounds of Opening and Closing at Middle DanTian. Then do your second half of the exercise by following the movements described in the Start by stepping forward with your left foot' section below.

If you start the Natural Walking exercise with your left foot, you have completed the exercise. Do the Concluding exercise facing the direction appropriate to your health conditions and rest for about 10 to 15 minutes

**Start by stepping forward with your left foot**

If you are to start the exercise with your left foot according to the health conditions or gender, this is the first half of the exercise.

If you are to start the exercise with your right foot, this is the second half of the exercise.

Get into the ready position as described in section "1.2.5 Ready position".

1. Bring your left foot forward a small step with toes raised and pointing straight ahead.

If this is your first step, do not move your hands. Otherwise move both of your hands towards the left while you shift your weight and step forward.

Lower your left heel gently. When the heel touches the ground, your right hand is in front of your Middle DanTian and your left hand by the side of your left hip. At this time, breathe in twice (xi xi).

Lower your left sole gently onto the ground and shift your weight to the front (left leg).

1. While shifting your weight forward, move your hands towards the right, step forward your right foot with toes raised and pointing straight ahead. If this is your fourth step or every fourth step thereafter, simultaneously turn your head and your waist towards the right. Lower your right heel gently. When the heel touches the ground, your left hand is in front of your Middle DanTian and your right hand by the side of your right hip. If you have turned your waist and head, your head is at about 60° and your waist at about 45° towards the right. At this time, exhale once (hu-).

Lower your right sole gently onto the ground and shift your weight to the front (right leg). If you have turned your waist and head, turn back to front-facing.

You can turn your waist and head every 6 steps if you prefer.

Continue to walk by repeating the above. After walking for 20 minutes, move up your foot from behind to the Relaxed Standing posture.

If you start the Natural Walking exercise with your right foot, you have completed the exercise. Do the Concluding exercise facing the direction appropriate to your health conditions and rest for about 10 to 15 minutes.

If you start the Natural Walking exercise with your left foot, you have completed the first half of the exercise. Do three rounds of Opening and Closing at Middle DanTian. Then continue to do the second half by following the movements described in the Start by stepping forward with your right foot section above.

The co-ordination of various movements can be summarized in the following

tables:

**Start by stepping forward with right foot:**

| Heel | Right heel touches the ground. | Left heel  touches the  ground. | Right heel  touches the  ground. | Left heel  touches the  ground. |
| --- | --- | --- | --- | --- |
| Hand | Left hand in front of Middle DanTian.  Right hand  beside the  right hip. | Right hand in  front of Middle  DanTian.  Left hand  beside the left  hip. | Left hand in  front of Middle  DanTian.  Right hand  beside the  right hip. | Right hand in  front of Middle  DanTian.  Left hand  beside the left  hip. |
| Breathing | Inhale twice. | Exhale once. | Inhale twice. | Exhale once. |
| Turn | Facing front. | Facing front. | Facing front. | Turn head  and waist to  left (if turn  every 4 steps). |

**Start by stepping forward with left foot:**

| Heel | Left heel touches the ground. | Right heel  touches the  ground. | Left heel  touches the  ground. | Right heel  touches the  ground. |
| --- | --- | --- | --- | --- |
| Hand | Right hand in front of Middle DanTian.  Left hand  beside the  right hip. | Left hand in  front of Middle  DanTian.  Right hand  beside the left  hip. | Right  front of Middle  DanTian.  Left hand  beside the  left hip. | Left hand in  front of Middle  DanTian.  Right hand  beside the right  hip. |
| Breathing | Inhale twice. | Exhale once. | Inhale twice. | Exhale once. |
| Turn | Facing front. | Facing front. | Facing front. | Turn head  and waist to  right (if turn  every 4 steps). |

To help with your coordination, you can think in your mind: in in out, in in turn, in in out , in in turn, ........ or

xi xi hu-, xi xi turn, xi xi hu-, xi xi turn ........

**1.3 Things to pay attention to**

1. ﻿﻿﻿You need to master the essentials. Practise like leisure walking, pay attention to raising the toes high, not walking with the feet pointing outward, keeping the knees slightly bent, lowering your hips and relaxing your waist, guiding the qi to Middle DanTian, and turning your head and waist naturally.
2. ﻿﻿﻿Touch the palate behind the upper teeth lightly with the tip of your tongue. The best is when you can just barely feel the touch. This enables the linking of Du Mai (督脉Governing Vessel) and Ren Mai (任脉 Conception Vessel). If there is an excessive amount of saliva when you practise the exercise, stop and stand in a Relaxed Standing posture. Swallow the saliva in three portions and feel the saliva reaching your Middle DanTian. Then do a set of Three Opening and Closing at Middle DanTian and continue walking. If you are familiar with the movements, you may swallow the saliva while walking.
3. ﻿﻿﻿Keep the Relaxed Standing posture always while practising the exercise. Walk naturally in small steps, lower your heel onto the ground lightly and gently, relax your shoulder, elbow and wrist when moving your arms. Keep a small gap between your upper arms and your body, relax your waist and hips. It is most important to relax, be tranquil and natural.
4. ﻿﻿﻿Walk at a moderate pace that is suitable for you without a stuffy or suffocating feeling. People with cardiovascular, cerebrovascular or liver disease, or people who are weak can practise at a slower speed. Cancer patients should practise at a faster speed. However, you must find a speed suitable for you according to your health and physical conditions.
5. Though your eyes are not closed while practising, you should "not look at anything you see" and "not listen to anything you hear". Keep your eyesight level while turning your head.

**2 Dian Bu Gong (Step-and-Tap Method) 点步功**

There are four types of Step-and-Tap exercises: one-step-one-tap, one-step-three-taps, two-steps-one-tap, and three-steps-one-tap. They all use moderate (medium strength) Feng breathing (breathe in twice, breathe out once [the breathe-out equals 2 breathe-ins]).

The Step-and-Tap exercises can prevent and treat cancer; reduce low-grade fever; reduce inflammation and relieve pain; and regulate the internal organs, so it is effective for chronic patients. Patients with nephritis, hepatitis, pneumonia, bronchitis, and emphysema can use these exercises as their main practice exercises.

The Step-and-Tap exercises are effective in strengthening our five internal organs. By touching the ground with the heel, it can stimulate the Kidney Meridian to strengthen the kidney. Through Feng breathing, the Lung Meridian can strengthen the lung. By tapping the ground with the underside of the big toe, this can stimulate the Spleen Meridian's YinBai point (approximate 0.3 cm next to the nail angle on the inner side of the big toe) and the Liver Meridian's DaDun point (approximate 0.3 cm beside the nail angle on the outer side of the big toe), to invigorate the Spleen Meridian and Liver Meridian respectively. In addition, using the middle finger and ring finger of the two hands to touch the middle of the palm can stimulate the Pericardial Meridian. Therefore, these exercises can stimulate and regulate the heart, liver, spleen, lungs and kidneys.

The Step-and-Tap exercises can be practised separately with Preparatory and Concluding exercises before and after each Step-and-Tap exercise, or practise them continuously with the Preparatory exercise before the first exercise and the Concluding exercise after the last exercise, and three rounds of Opening and Closing at Middle DanTian in between each Step-and-Tap exercise. Again, you should practise according to your health condition and physical ability.

﻿﻿﻿

**Exercise sequence:**

Preparatory 🡪 any Step-and-Tap exercise 🡪 Concluding

OR

Preparatory

- ﻿﻿One-Step-One-Tap Exercise or One-Step-Three-Taps Exercise
- Three Opening and Closing at Middle DanTian
- ﻿﻿Two-Steps-One-Tap Exercise (start by stepping forward with left or right foot)
- Three Opening and Closing at Middle DanTian
- ﻿﻿Second half of Two-Steps-One-Tap (start by stepping forward with the other foot)
- ﻿﻿Three Opening and Closing at Middle DanTian
- ﻿﻿Three-Steps-One-Tap Exercise
- ﻿﻿Concluding

**2.1 One-Step-One-Tap Exercise 一步点**

When practising this exercise, you tap the underside of your big toe once after each step, and complete a cycle of Feng breathing with each step and tap. Rhythmically complete in two beats: breathe-in (inhaling) twice is one beat, and breathe-out (exhaling) is one beat.

**Method**

Slowly open your eyes after the Preparatory exercise (same as Natural

Walking exercise) and get into the ready position (see Figure 2-1a).

**Start by stepping forward with your right foot first as** an example

Step forward with your right foot with toes raised. When your right heel touches the ground, simultaneously inhale twice with your nose (Figure 2-1b). Then lay your right sole flat on the ground and shift your weight forward, turn your waist approximately 45° and your head approximately 60° towards the left, swing along your arms with the movement of your right hand to the front of your Middle DanTian and your left hand to the side of your left hip. Slowly raise your left foot and tap the underside of your big toe gently onto the ground about 10 cm beside the middle (arch) of your right foot, and simultaneously breathe out (equals 2 breathe-ins) (Figure 2-1c, d). Then continue to start your next step, the left foot.

**The next step with your left foot:** Step forward with your left foot with toes raised. When your left heel touches the ground, simultaneously inhale twice with your nose. Then lay your left sole flat on the ground and shift your weight forward, turn your waist approximately 45° and your head approximately 60° towards the right, swing along your arms with the movement of your left hand to the front of your Middle DanTian and your right hand to the side of your right hip. Slowly raise your right foot and tap the underside of your big toe gently onto the ground about 10 cm beside the middle (arch) of your left foot, and simultaneously breathe out (equals 2 breathe-ins). Then continue to start with your next step, the right foot.

To follow the above procedures repeatedly until you practise for about 20 minutes, move up your back foot to the Relaxed Standing posture. Do three rounds of Opening and Closing at Middle DanTian, then continue with the Two-Steps-One-Tap exercise.


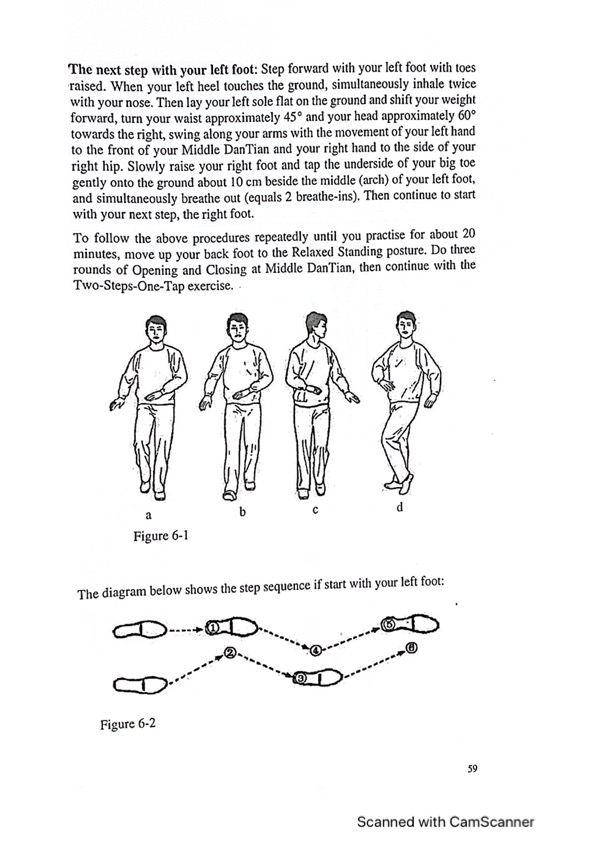


Figure 2-1

The diagram below shows the step sequence if start with your left foot:


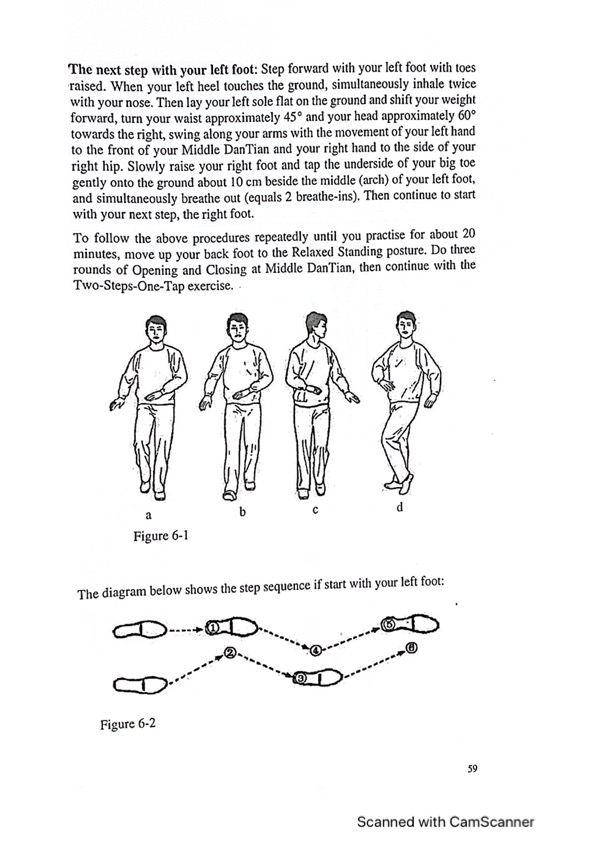


Figure 2-2

The co-ordination of various movements can be summarized in the following tables:

**Start by stepping forward with right foot:**

| Foot | Right heel touches the ground. | Weight on right foot. | Weight on right foot.  Tap left big toe. | Weight on right foot.  Left big toe on ground. |
| --- | --- | --- | --- | --- |
| Hand | Left hand in front of Middle DanTian.  Right hand beside right hip. | Right hand in front of Middle DanTian.  Left hand beside left hip. | Right hand in front of Middle DanTian.  Left hand beside left hip. | Right hand in front of Middle DanTian.  Left hand beside left hip. |
| Breathing | Breathe in twice. |  | Breathe out once. |  |
| Turn | Body and head facing front. | Turn waist (approx. 45° and head (approx. 60°) to left. |  | Turn head and waist to front facing. |

**Start by stepping forward with left foot:**

| Foot | Left heel touches the ground | Weight on left foot. | Weight on left foot.  Tap right big toe. | Weight on left foot.  Right big toe on ground. |
| --- | --- | --- | --- | --- |
| Hand | Right hand in front of Middle DanTian.  Left hand beside left hip. | Left hand in front of Middle DanTian.  Right hand beside right hip | Left hand in front of Middle DanTian.  Right hand beside right hip. | Left hand in front of  Middle DanTian.  Right hand beside right hip. |
| Breathing | Breathe in twice. |  | Breathe out once. |  |
| Turn | Body and head facing front. | Turn waist (approx. 45° and head (approx. 60°) to right. |  | Turn head and waist to front facing. |

**2.2 One-Step-Three-Taps Exercise 一步三点**

Basically the same as One-Step-One-Tap except that instead of one tap, you tap 3 times with one cycle of Feng breathing and one natural breathing. Rhythmically the One-Step-Three-Taps exercise is complete in four beats.

Feng breathing: breathe-in twice is one beat, and breathe-out is one beat; natural breathing: breathe-in is one beat, breathe-out is one beat. This exercise is suitable for patients with liver, spleen disease.

**Method**

**Start by stepping forward with your left foot first** as an example

Take a step forward with your left foot, raise your toes high, when the heel touches gently on the ground, breathe in twice (one beat). At this time, the right hand should be in front of your Middle DanTian and the left hand is beside your left hip. Shift your weight forward, swing your left hand to the Middle DanTian and right hand to the right hip, lift your right foot and, use your right big toe (the underside) lightly tapping the ground once approximately 10 cm from the middle (arch) of your left foot. Simultaneously, turn your waist approximately 45° to the right and your head approximately 60°, breathe out (equals 2 breathe-ins), close your hands gently such that the middle and ring fingers of both hands touch the middle of your palms once. Raise your right foot, use your right big toe (the underside) lightly tapping the ground again approximately 10 cm from the middle (arch) of your left foot, breathe in (natural breathing). Again, raise your right foot, use your right big toe (the underside) lightly tapping the ground the third time approximately 10 cm from the middle (arch) of your left foot, breathe out (natural breathing). Open your hands slowly, and turn your head and waist back to front facing. Then continue to start your next step, the right foot.

**The next step with your right foot:** Take a step forward with your right foot, raise your toes high, when the heel touches gently on the ground, breathe in twice. At this time, the left hand should be in front of your Middle DanTian and the right hand is beside your right hip. Shift your weight forward, swing your right hand to the Middle Dan Tian and left hand to the left hip, lift your left foot and use your left big toe (the underside) lightly tapping the ground once approximately 10 cm from the middle (arch) of your right foot. Simultaneously, turn your waist approximately 45° to the left and your head approximately 60°, breathe out (equals 2 breathe-ins), close your hands gently such that the middle and ring fingers of both hands touch the middle of your palms once. Raise your left foot, use your left big toe (the underside) lightly tapping the ground again approximately 10 cm from the middle (arch) of your left foot, breathe in (natural breathing). Again, raise your left foot, use your left big toe (the underside) lightly tapping the ground the third time approximately 10 cm from the middle (arch) of your right foot, breathe out (natural breathing). Open your hands slowly and turn our head and waist back to front facing. Then continue to start your next step, the left foot.

To follow the above procedures repeatedly until you practise for about 20 minutes, move up your back foot to the Relaxed Standing posture. Do three rounds of Opening and Closing at Middle DanTian, then continue with the Two-Steps-One-Tap exercise, or do the Concluding exercise, rest for a while and continue with another exercise.

You can do the One-Step-Three-Taps as an independent exercise. This can also be practised instead of the One-Step-One-Tap exercise.

**2.3 Two-Steps-One-Tap Exercise 二步点**

When practising this exercise, you tap the big toe (the underside) once after two steps, and complete a cycle of Feng breathing in each round of two steps and one tap. Rhythmically the Two-Steps-One-Tap exercise is complete in four beats: breathe-in twice is two beats, and breathe-out is two beats. It can be done as an independent exercise or done after the One-Step-One-Tap exercise. With this exercise, you have to switch legs: practise 10 minutes starting (and tapping) with one leg (see section 4.2.1 Which Leg to Start), then another 10 minutes with the other leg. That total 20 minutes to complete the practice.

**Method**

**Start by stepping forward with your left foot first** as an example

Take a step forward with your left foot, raise your toes high, when the heel touches gently on the ground, simultaneously breathe in once (one beat). At this time, the right hand should be in front of your Middle DanTian and the left hand is beside your left hip. Shift your weight forward with both arms swinging and step forward with your right foot, the second step, left hand to the Middle DanTian and right hand to the right hip. When the heel touches gently on the ground, breathe in (one beat) again, and the left hand is now in front of your Middle DanTian and the right hand also is by your right hip. Lower your right sole gently on the ground to bring your weight forward, simultaneously bring your right hand to the Middle DanTian and the left hand to your left hip. Also, turn your waist approximately 45° to the left and your head approximately 60°. Simultaneously use your left big toe (the underside) to lightly tap the ground approximately 10 cm from the middle (arch) of your right foot, and breathe out (two beats). Continue with the above procedures for 10 minutes, bring your back foot up to the Relaxed Standing posture, do three rounds of Opening and Closing at Middle Dan Tian. Change to start (and tap) with your right foot.

**The right foot:** Take a step forward with your right foot, raise your toes high, when the heel touches gently on the ground, simultaneously breathe in once (one beat). At this time, the left hand should be in front of your Middle DanTian and the right hand is beside your right hip. Shift your weight forward with both arms swinging and step forward with your left foot, the second step, right hand to the Middle DanTian and left hand to the left hip. When the heel touches gently on the ground, breathe in (one beat) again, and the right hand is now in front of your Middle DanTian and the left hand also is by your left hip. Lower your left sole gently on the ground to bring your weight forward, simultaneously bring your left hand to the Middle Dan Tian and the right hand to your right hip. Also, turn your waist approximately 45° to the right and your head approximately 60°. Simultaneously use your right big toe (the underside) to lightly tap the ground approximately 10 cm from the middle (arch) of your left foot, and breathe out (two beats). Continue with the above procedures for 10 minutes, bring your back foot up to the Relaxed Standing posture, do three rounds of Opening and Closing at Middle Dan Tian.

After you have completed the full set total 20 minutes (left foot first then right foot, or right foot first then left foot), bring forward your back foot to the Relaxed Standing posture and do three rounds of Opening and Closing at Middle DanTian. You can either continue with the Three-Steps-One-Tap exercise, or do the Concluding exercise and rest.

The diagram below shows the step sequence if start with your left foot:


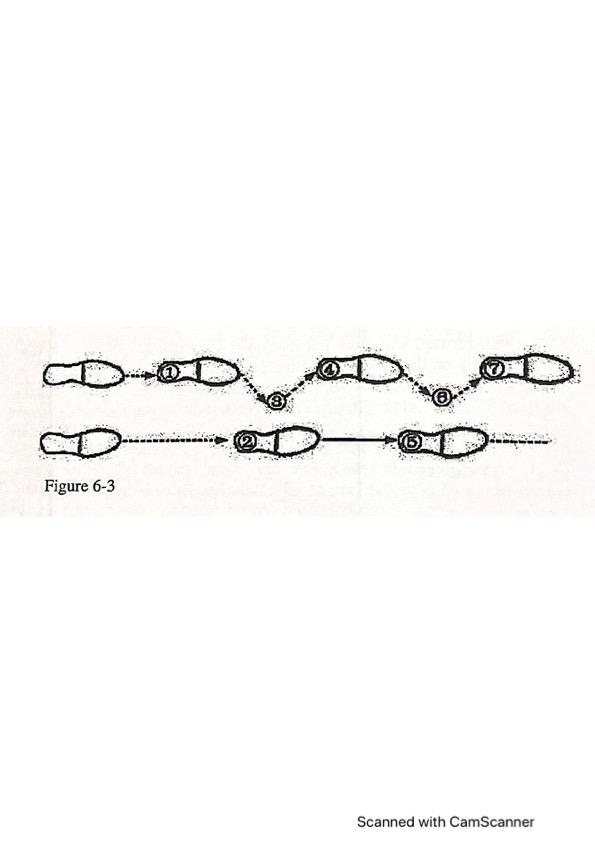


Figure 2-3

The movements can be summarized in the following tables:

**Start by stepping forward with right foot:**

|  | 1 | 2 | 3 | | 4 |
| --- | --- | --- | --- | --- | --- |
| Foot | Right heel touches the ground. | Left heel touches the ground. | Weight on left foot. | Weight on left foot.  Tap right big toe. | Weight on left foot.  Right big toe on ground |
| Hand | Left hand in front of Middle DanTian.  Right hand beside right hip. | Right hand in front of Middle DanTian.  Left hand beside left hip. | Left hand in front of Middle DanTian.  Right hand beside right hip. | Left hand in front of Middle DanTian.  Right hand beside right hip. | Left hand in front of Middle Dan Tian.  Right hand beside right hip. |
| Breathing | Breathe in once. | Breathe in once. |  | Breathe out once. |  |
| Turn | Body and head facing front. | Body and head facing front. | Turn waist (approx.  45° and head (approx. 60° to right. |  | Turn head and waist to front facing. |

**Start by stepping forward with left foot:**

|  | 1 | 2 | 3 | | 4 |
| --- | --- | --- | --- | --- | --- |
| Foot | Left heel touches the ground. | Right heel touches the ground. | Weight on right foot. | Weight on right foot.  Tap left big toe. | Weight on right foot.  Left big toe On ground. |
| Hand | Right hand in front of Middle DanTian.  Left hand beside left hip. | Left hand in front of Middle DanTian.  Right hand beside left hip. | Right hand in front of Middle DanTian.  Left hand beside right hip. | Right hand in front of Middle DanTian.  Left hand beside right hip. | Right hand in front of Middle Dan Tian.  Left hand beside right hip. |
| Breathing | Breathe in once. | Breathe in once. |  | Breathe out once. |  |
| Turn | Body and head facing front. | Body and head facing front. | Turn waist (approx.  45° and head (approx. 60° to left. |  | Turn head and waist to front facing. |

**2.4 Three-Steps-One-Tap Exercise 三步点**

When practising this exercise, tap your big toe (the underside) on the ground with your back foot after three steps coordinating with one cycle of Feng breathing and one natural breathing. Rhythmically the Three-Steps-One-Tap exercise is complete in six beats: breathe-in is one beat; breathe-in again is one beat; and breathe-out is two beats, your back foot tapping uses natural breathing with breathe-in as one beat, breathe-out as one beat.

**Method**

**Start by stepping forward with your right foot first** as an example

Take a step forward with your right foot, raise your toes high, the left hand be in front of your Middle DanTian and the right hand is beside your right hip, the heel touches gently on the ground and at the same time breathe in once. Step forward with your left foot (shift weight forward) with both arms swinging, right hand to the Middle DanTian and left hand to the left hip, when the heel touches gently on the ground, you simultaneously breathe in again. Move forward with your right foot (shift weight forward), coordinate with your hands again, left to the Middle DanTian and right to the right hip, breathe-out (two beats). While exhaling, shift your weight forward, turn your upper body, the waist to the left approximately 60° and your head also to approximately 60°. Your right hand should now be in front of your Middle DanTian and your left hand beside the left hip. Raise your left (back) foot and tap lightly on the ground where your back foot was located (Figure 6-4a), and simultaneously breathe in and out (natural breathing). You have now completed the Three-Steps-One-Tap. Continue with your left foot.


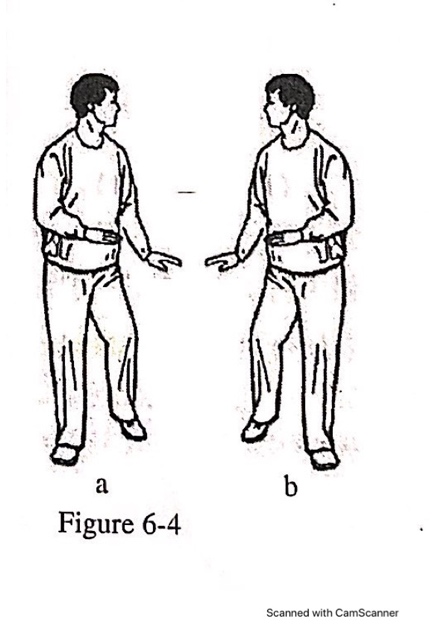


**The left foot**: Take a step forward with your left foot, raise your toes high, the right hand be in front of your Middle DanTian and the left hand is beside your left hip, the heel touches gently on the ground and at the same time breathe in once. Step forward with your right foot (shift weight forward) with both arms swinging, left hand to the Middle DanTian and right hand to the right hip, when the heel touches gently on the ground, you simultaneously breathe in again. Move forward with your left foot (shift weight forward), coordinate with your hands again, right to the Middle DanTian and left to the left hip, breathe-out (two beats). While exhaling, shift your weight forward, turn

your upper body, the waist to the right approximately 60° and your head also to approximately 60°. Your left hand should now be in front of your Middle Dan Tian and right hand beside the right hip. Raise your right (back) foot and tap lightly on the ground where your back foot was located, and simultaneously breathe in and out (natural breathing) (Figure 6-4b).

Repeat the above procedures for about 20 minutes, then do the Concluding exercise. Rest for about 15 minutes and you can practise another exercise or end the day practice.

The diagram below shows the step sequence if start with your left foot:


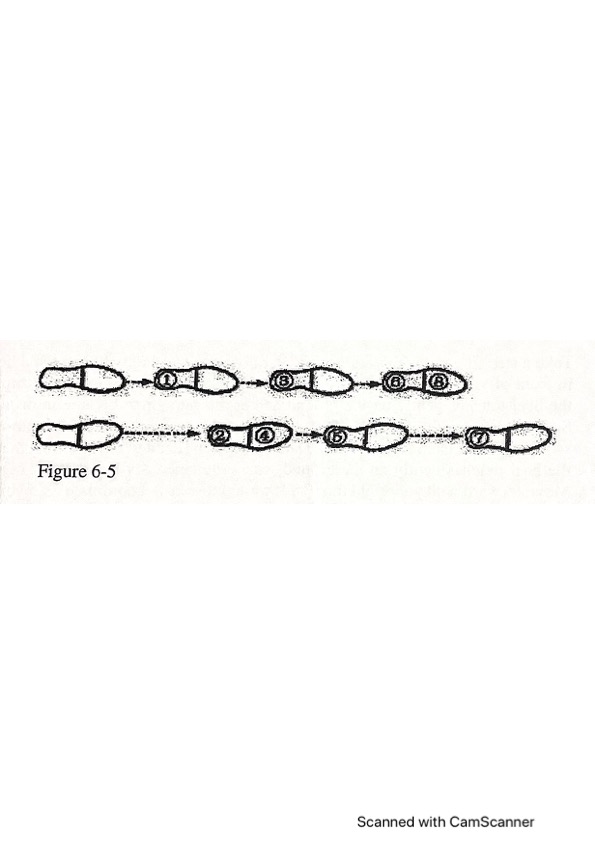


Figure 2-5

The movements can be summarized in the following tables:

**Right foot cycle (start by stepping forward with right foot):**

| Foot | Right heel touches the ground. | Left heel touches the ground. | Right heel touches the ground. | Weight on right. | Weight on right. Tap left big toe. | Weight on right. Left big toe on ground. |
| --- | --- | --- | --- | --- | --- | --- |
| Hand | Left hand in front of Middle DanTian.  Right hand beside right hip. | Right hand in front of Middle DanTian.  Left hand beside left hip. | Left hand in front of Middle DanTian.  Right hand beside right hip. | Right hand in front of Middle DanTian.  Left hand beside left hip. | Keep the hands in the same position. | Keep the hands in the same position. |
| Breathing | Breathe in once. | Breathe once. | Breathe out once. | | Natural  breathing. | Natural breathing. |
| Turn |  |  |  | Turn waist (approx. 60°) and head (approx. 60°) to left. |  | Turn waist and head back to front facing. |

**Left foot cycle (start by stepping forward with left foot):**

| Foot | Left heel touches the ground. | Right heel touches the ground. | Left heel touches the ground. | Weight on left. | Weight on left. Tap right big toe. | Weight on left.  Right big toe on ground. |
| --- | --- | --- | --- | --- | --- | --- |
| Hand | Right hand in front of Middle DanTian.  Left hand beside right hip. | Left hand in front of Middle DanTian.  Right hand beside left hip. | Right hand in front of Middle DanTian.  Left hand beside right hip. | Left hand in front of Middle DanTian.  Right hand beside left hip. | Keep the hands in the same position. | Keep the hands in the same position. |
| Breathing | Breathe in once. | Breathe once. | Breathe out once. | | Natural  breathing. | Natural breathing. |
| Turn |  |  |  | Turn waist (approx. 60°) and head (approx. 60°) to right. |  | Turn waist and head back to front facing. |

**2.5 Things to pay attention to**

1. ﻿﻿﻿The Step-and-Tap exercises can be practised continuously. Practise each exercise for about 20 minutes with three rounds of Opening and Closing at Middle DanTian in between each and complete with the Concluding exercise. Those who are physically weak can practise the Step-and-Tap exercises separately or just practise any one of them, also the timing can be reduced to less than 20 minutes. Again, you should practise according to your health condition and physical ability and gradually build up practising each exercise to about 20 minutes.
2. Patients with heart disease breathe lighter while practising this/these exercises. While doing the toe tapping, use the middle finger and ring finger to gently touch the inside of the palm. Patients with severe heart disease do not practise these exercises; if practise, use natural breathing instead of Feng breathing.
3. The breathing of these exercises is to breathe in (inhale) on the front, and breathe out (exhale) on the side, the side where you turn your waist and head and tap.
4. Walk with small steps.
5. When turning, keep your head upright, do not tilt your head.
6. Relax your leg/foot, tap lightly and pause/hold for a short moment.

**3. Sheng Jiang Kai He Fa (Ascending, Descending, Opening and Closing**

**Method)**

This is a stationary exercise to be practised with eyes slightly closed while breathing naturally. This can be done independently or combined with Preparatory and Concluding exercises. You should be in a relaxed and natural state when practising this exercise so that you can perform calmly and naturally to stimulate the inner qi flow. When the qi flow is strong and not obstructed, it will have a good effect on the prevention and treatment of cancer, and it will also have a satisfactory effect on heart and other chronic diseases. Try to practise this exercise slowly, do not rush. Generally, it takes about 15-20 minutes to complete the set of 8 cycles. You start with your right or left foot in front first according to your health conditions or gender as described in ‘4.2.1 Which Leg to Start', and practise the exercise facing the 4 directions in sequence. Then switch with the other foot in front and practise the exercise facing the 4 directions in reverse order as described in 8.2 Complete set sequence. Each direction takes about 2 minutes to practise.

There are two main benefits by practising this exercise:

1. Vitalize and invigorate the movement of qi inside the body:

The qi inside your body is constantly moving up and down and circulates in a continuous loop around your body, 24 hours a day. The yin and yang alterations in the up, down, open and close movements in this exercise invigorate and strengthen your inner qi, and trigger the exchange of inner and outer qi's.

1. ﻿﻿﻿Activate and strengthen your essence, qi, spirit (精, 气, 神) - the three treasures of a human being:

This benefit can be achieved by the opening and closing movements in front of:

- ﻿﻿ Upper DanTian - YinTang (印堂), the acupressure point in the forehead between the eyebrows, where the spirit inhabits.
- ﻿﻿Middle DanTian - QiHai (气海), about 1.5 cun (2-fingers width) below the navel, where vital inner qi is stored.
- Lower DanTian - HuiYin (会阴), the acupressure point between the reproductive organ and anus, where the essence resides.

**3.1 Essentials**

Other than up, down, open and close movements of the hands, the body weight also shifts back and forth between the legs. You can use the following to help you remember and coordinate the movements:

- ﻿﻿There are three up, three down, three open and three close movements.
- ﻿﻿When the hands are moving **up, shift** your weight to the **front** leg.
- ﻿﻿When the hands are moving **down, shift** your weight to the **back** leg.
- ﻿﻿When the hands are **opening** (moving to both sides), your weight is on the **back** leg.
- ﻿﻿When the hands are **closing** (moving towards the center), **shift** the weight to the **front** leg.
- ﻿﻿Change your finger gestures when your hands moving up or down reaching your ShanZhong (膻中, at chest level).

**3.2 Complete set sequence**

If you are to **start the exercise with your right foot:**

Preparatory

- ﻿﻿Step forward with your right foot, do four cycles

Turn 90° to the right after the first, second and third cycles

- ﻿﻿Three Opening & Closing at Middle DanTian
- ﻿Step forward with your left foot, do four cycles

Turn 90° to the left after the fifth, sixth and seventh cycles

- Concluding


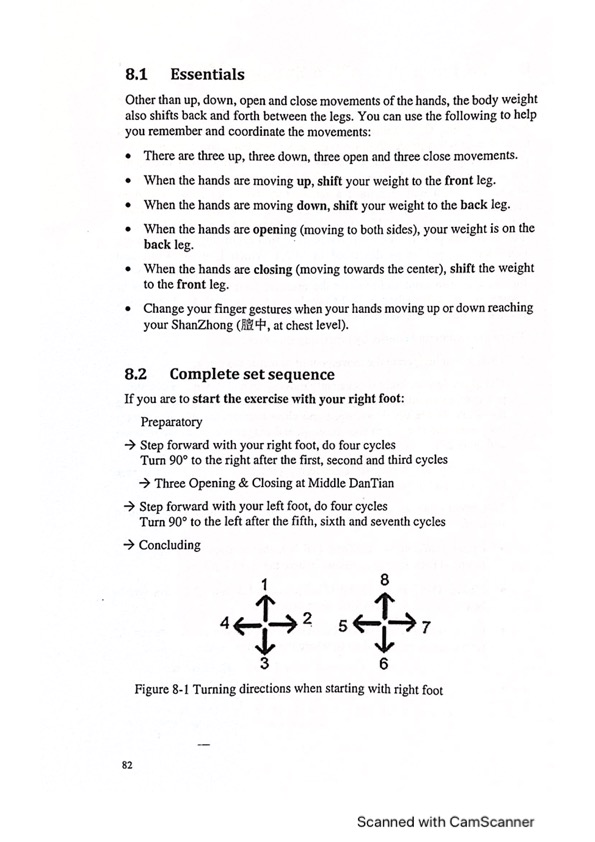


Figure 3-1 Turning directions when starting with right foot

If you are to **start the exercise with your left foot**:

Preparatory

- ﻿﻿Step forward with your left foot, do four cycles

Turn 90° to the left after the first, second and third cycles.

- ﻿﻿Three Opening & Closing at Middle DanTian
- Step forward with your right foot, do four cycles.

Turn 90° to the right after the fifth, sixth and seventh cycles

- Concluding


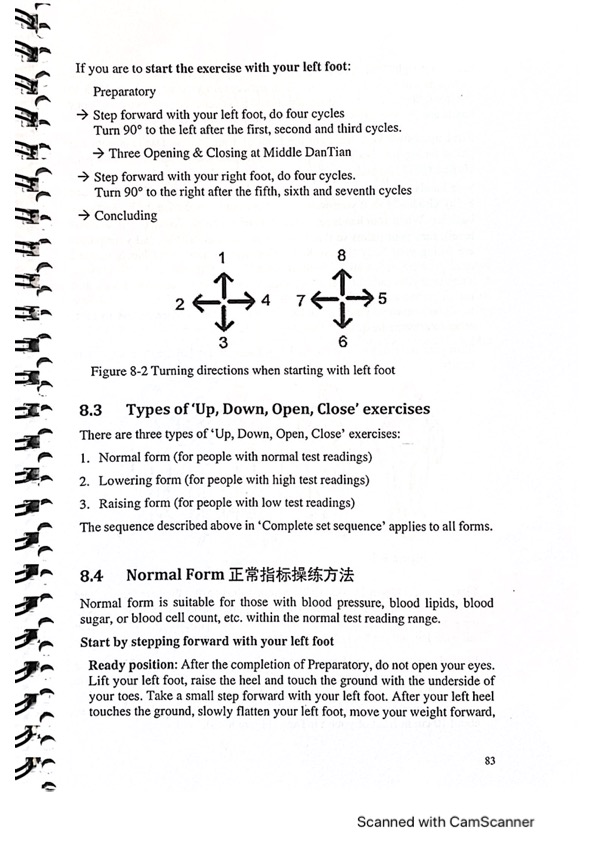


Figure 3-2 Turning directions when starting with left foot

**3.3 Types of 'Up, Down, Open, Close' exercises**

There are three types of Up, Down, Open, Close' exercises:

1. ﻿﻿﻿Normal form (for people with normal test readings)
2. ﻿﻿﻿Lowering form (for people with high test readings)
3. ﻿﻿﻿Raising form (for people with low test readings)

The sequence described above in 'Complete set sequence applies to all forms.

**3.4 Normal Form 正常指标操练方法**

Normal form is suitable for those with blood pressure, blood lipids, blood sugar, or blood cell count, etc. within the normal test reading range.

**Start by stepping forward with your left foot**

**Ready position**: After the completion of Preparatory, do not open your eyes. Lift your left foot, raise the heel and touch the ground with the underside of your toes. Take a small step forward with your left foot. After your left heel touches the ground, slowly flatten your left foot, move your weight forward, raise your right heel, turn to the right, adjust your back foot into a diagonal T-step as shown in Figure 3-3a. Then slowly turn your upper body back to the front. Stand steady, your weight should be in between your two legs. Your hands are by your sides. You are now ready to start (Figure 3-3a).

**First up**: Move vour hands to the front of your Middle DanTian with the palms facing your body, until the middle fingers touch each other with your HuKou (虎口, the arch between the thumb and index finger) facing up. Move your hands up along the route of Ren Mai (任脉 Conception Vessel) (Figure 3-3b). Gradually shift your weight to your front leg and raise the heel of your back leg. When your hands reach the front of your ShanZhong (膻中, at chest level), turn your palms so that your fingers are pointing up and your palms are facing your body (Figure 3-3c). Continue to move your hands upward until your fingertips are in front of your YinTang (between the eyebrows). Then turn vour palms to face each other. Keep your hands curved such that the fingertips and the wrists of both hands touch each other like holding a small ball between your palms, and keep each hand's fingers close to each other to prevent the qi from escaping (Figure 3-3d).

Shift your weight slowly to the back leg. Raise the heel of the front leg. Stand steady.


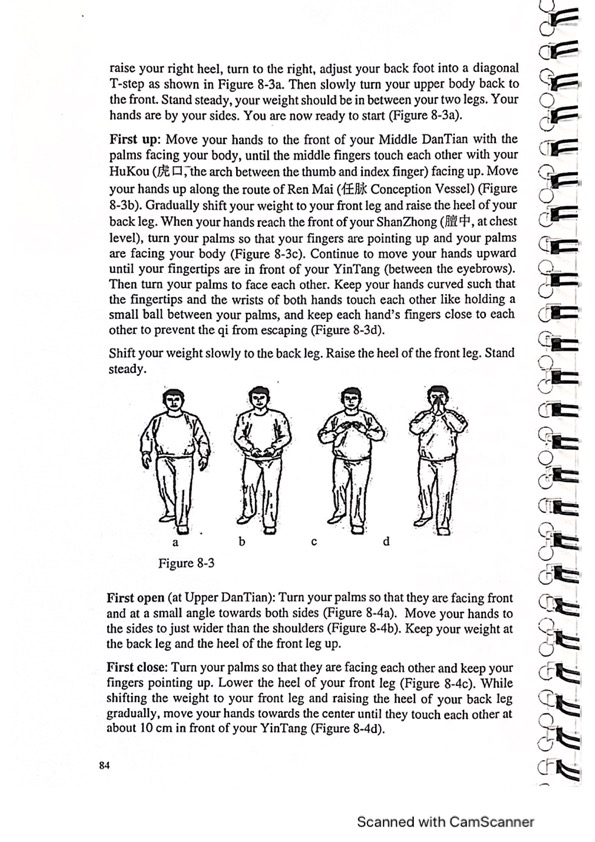


Figure 3-3

**First open** (at Upper DanTian): Turn your palms so that they are facing front and at a small angle towards both sides (Figure 3-4a). Move your hands to the sides to just wider than the shoulders (Figure 3-4b). Keep your weight at the back leg and the heel of the front leg up.

**First close**: Turn your palms so that they are facing each other and keep your fingers pointing up. Lower the heel of your front leg (Figure 3-4c). While shifting the weight to your front leg and raising the heel of your back leg gradually, move your hands towards the center until they touch each other at about 10 cm in front of your YinTang (Figure 3-4d).


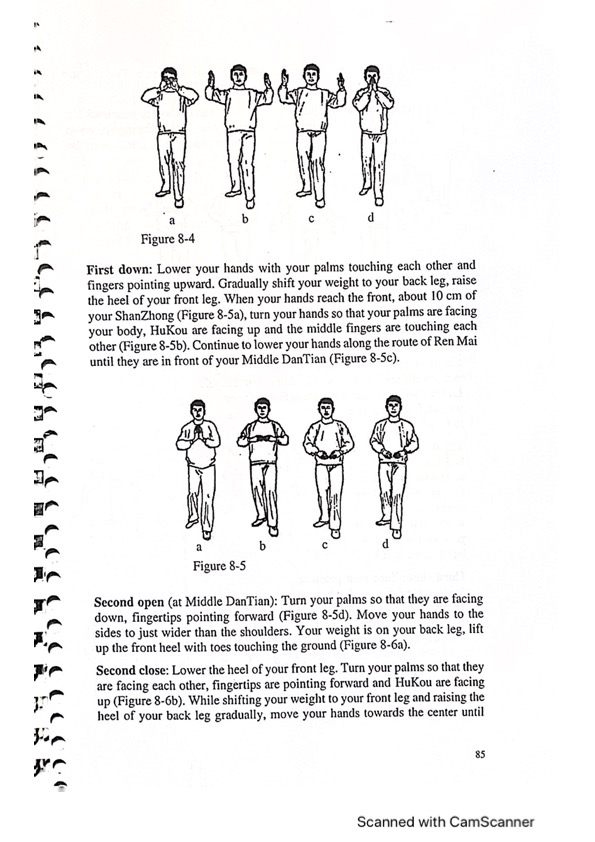


Figure 3-4

**First down**: Lower your hands with your palms touching each other and fingers pointing upward. Gradually shift your weight to your back leg, raise the heel of your front leg. When your hands reach the front, about 10 cm of your ShanZhong (Figure 3-5a), turn your hands so that your palms are facing your body, HuKou are facing up and the middle fingers are touching each other (Figure 3-5b). Continue to lower your hands along the route of Ren Mai until they are in front of your Middle DanTian (Figure 3-5c).


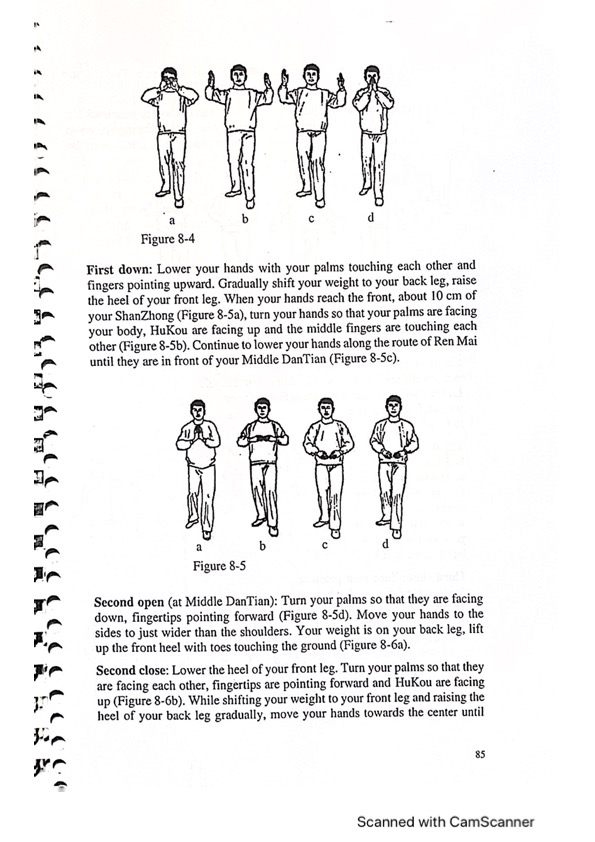


Figure 3-5

**Second open** (at Middle DanTian): Turn your palms so that they are facing down, fingertips pointing forward (Figure 3-5d). Move your hands to the sides to just wider than the shoulders. Your weight is on your back leg, lift up the front heel with toes touching the ground (Figure 3-6a).

**Second close**: Lower the heel of your front leg. Turn your palms so that they are facing each other, fingertips are pointing forward and HuKou are facing up (Figure 3-6b). While shifting your weight to your front leg and raising the heel of your back leg gradually, move your hands towards the center until your middle fingers touch each other in front of your Middle Dan Tian (Figure 3-6c).

**Second up:** Keep your weight on your front leg and the heel of your back leg raised. With your palms facing your body and your HuKou facing up, move your hands up along the route of Ren Mai from your Middle DanTian to the front of your ShanZhong (Figure 3-6d).


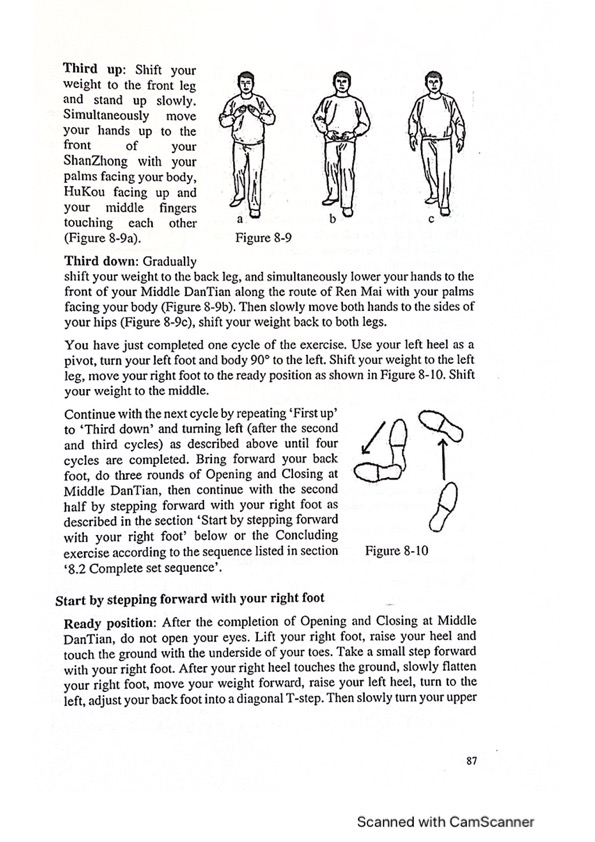

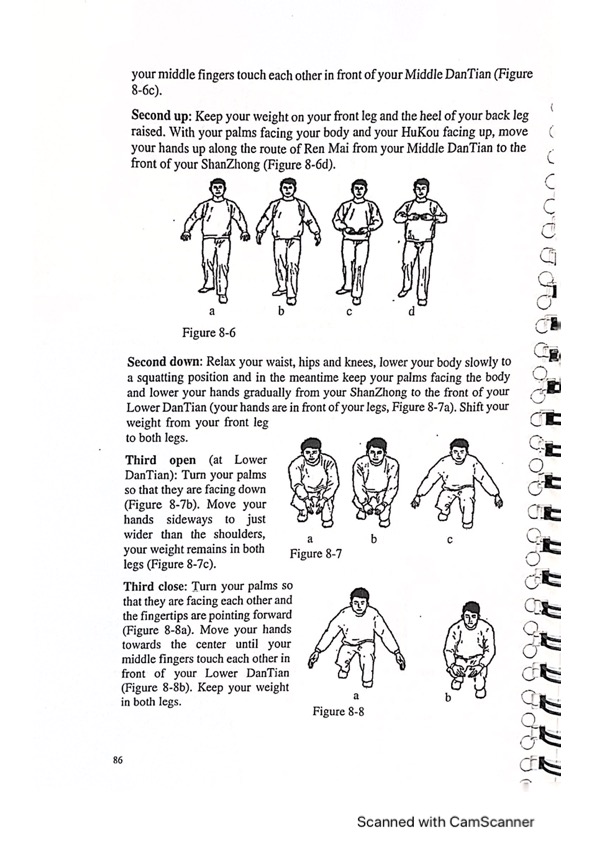


Figure 3-6

Figure 3-10

**Second down**: Relax your waist, hips and knees, lower your body slowly to a squatting position and in the meantime keep your palms facing the body and lower your hands gradually from your ShanZhong to the front of your Lower DanTian (your hands are in front of your legs, Figure 3-7a). Shift your weight from your front leg to both legs.


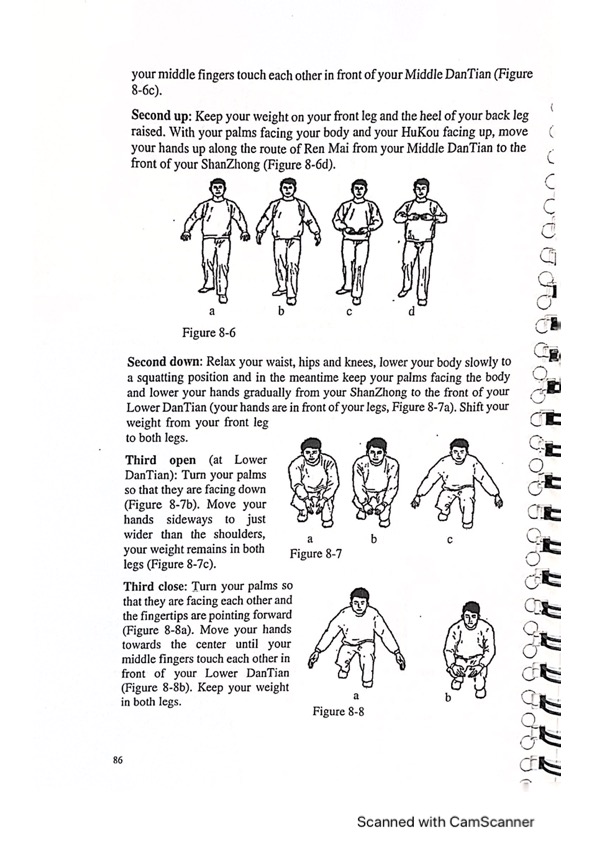


**Third open** (at Lower DanTian): Turn your palms so that they are facing down (Figure 3-7b). Move your hands sideways to just wider than the shoulders, your weight remains in both legs (Figure 3-7c).

Figure 3-7


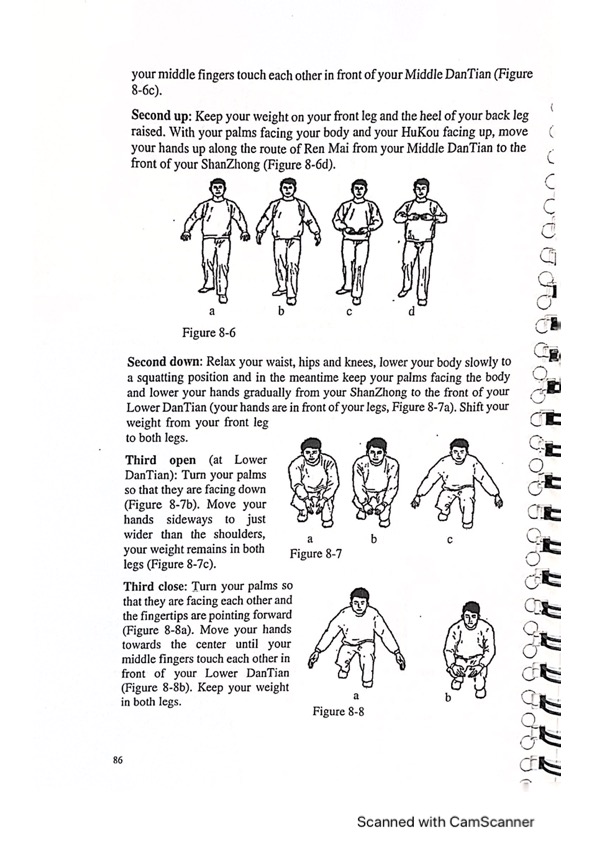


**Third close**: Turn your palms so that they are facing each other and the fingertips are pointing forward (Figure 8-8a). Move your hands towards the center until your middle fingers touch each other in front of your Lower DanTian (Figure 3-8b). Keep your weight in both legs.

Figure 3-8


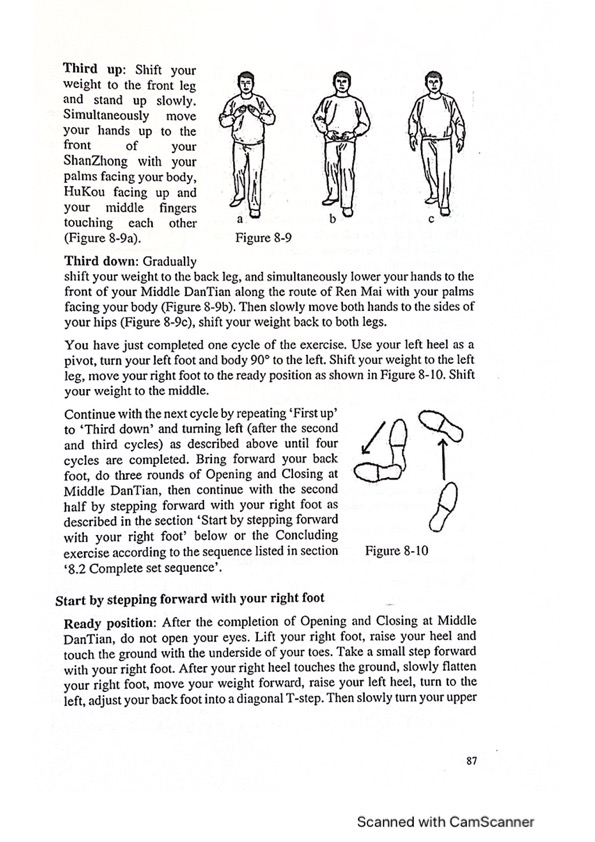


**Third up**: Shift your weight to the front leg and stand up slowly. Simultaneously move your hands up to the front of Your ShanZhong with your palms facing your body, HuKou facing up and your middle fingers touching each other (Figure 3-9a).

Figure 3-9

**Third down**: Gradually shift your weight to the back leg, and simultaneously lower your hands to the front of your Middle DanTian along the route of Ren Mai with your palms facing your body (Figure 3-9b). Then slowly move both hands to the sides of your hips (Figure 3-9c), shift your weight back to both legs.

You have just completed one cycle of the exercise. Use your left heel as a pivot, turn your left foot and body 90° to the left. Shift your weight to the left leg, move your right foot to the ready position as shown in Figure 3-10. Shift your weight to the middle.

Continue with the next cycle by repeating 'First up' to 'Third down' and turning left (after the second and third cycles) as described above until four cycles are completed. Bring forward your back foot, do three rounds of Opening and Closing at Middle DanTian, then continue with the second half by stepping forward with your right foot as described in the section Start by stepping forward with your right foot' below or the Concluding exercise according to the sequence listed in section ‘3.2 Complete set sequence’.

**Start by stepping forward with your right foot**

**Ready position**: After the completion of Opening and Closing at Middle DanTian, do not open your eyes. Lift your right foot, raise your heel and touch the ground with the underside of your toes. Take a small step forward with your right foot. After your right heel touches the ground, slowly flatten your right foot, move your weight forward, raise your left heel, turn to the left, adjust your back foot into a diagonal T-step. Then slowly turn your upper body back to the front. Stand steady, your weight should be in between your two legs. Your hands are by your sides. You are now ready to start.


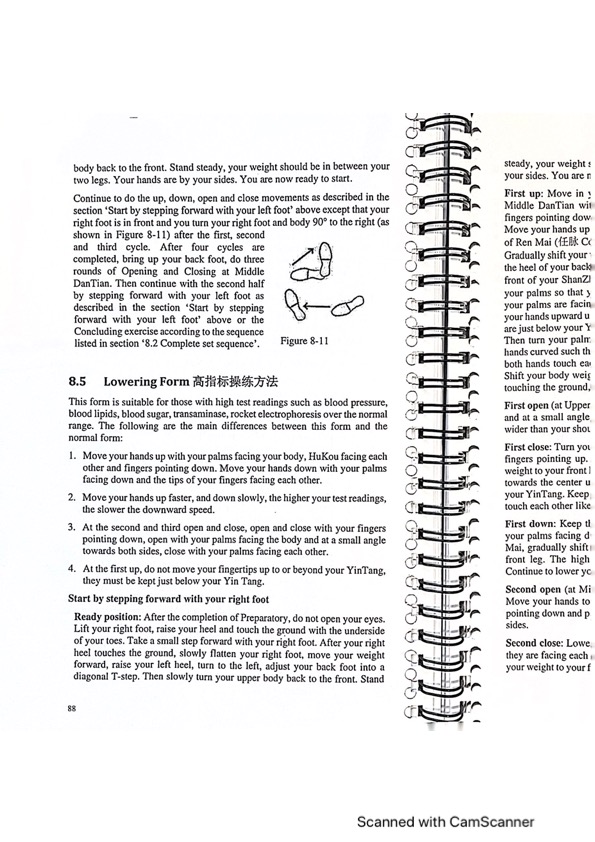
Continue to do the up, down, open and close movements as described in the section "Start by stepping forward with your left foot' above except that your right foot is in front and you turn your right foot and body 90° to the right (as shown in Figure 3-11) after the first, second and third cycle. After four cycles are completed, bring up your back foot, do three rounds of Opening and Closing at Middle DanTian. Then continue with the second half by stepping forward with your left foot as described in the section Start by stepping forward with your left foot' above or the Concluding exercise according to the sequence listed in section '3.2 Complete set sequence’.

Figure 3-11

**3.5 Things to pay attention to**

1. ﻿﻿﻿Patients with liver, gallbladder and eye diseases, regardless of their gender, start with the right leg first. Also, you step forward with the toes touching the ground first (instead of the heel) to invigorate your Liver Meridian, then flatten your sole. The toes touching the ground first are for all the directions.
2. ﻿﻿﻿During women's menstruation or patients with uterine prolapse, or people with gastroptosis, do half squatting or do not squat.
3. ﻿﻿﻿The 'Up, Down, Open, Close' exercise is a relaxation exercise. It's most beneficial if you can relax your whole body and calm your mind while practising this exercise. Do it slowly, it takes about 2 minutes for each direction.

Reference

Yu DY. *Guolin New Qigong Practice Manual.* Canada 2021.
