# Supplementary material for: Exploring Guolin Qigong (Mind-Body Exercise) for Improving Cancer Related Fatigue in Cancer Survivors: A Mixed Method Randomized Controlled Trial Protocol
Source: Integr Cancer Ther. 2024 May 17;23:15347354241252698. doi: 10.1177/15347354241252698 (PMC11102686; doi:10.1177/15347354241252698)
Supplement: sj-docx-3-ict-10.1177_15347354241252698 – Supplemental material for Exploring Guolin Qigong (Mind-Body Exercise) for Improving Cancer Related Fatigue in Cancer Survivors: A Mixed Method Randomized Controlled Trial Protocol [file sj-docx-3-ict-10.1177_15347354241252698.docx]

**Supplementary Material 3 Video links of Guolin Qigong training**

# **Feng Hu Xi Zhi Ran Xing Gong** (**Natural Wind Breathing Walking Exercise )**

1. Preparatory exercise预备功

<https://www.youtube.com/watch?v=Gk28UoO9gzo&list=PL6e3KTUbluTaDTLxxyLi1mDUBVqWJamID&index=7>

2.Natural Walking Exercise

<https://www.youtube.com/watch?v=z6AQjF4GyK4&list=PL6e3KTUbluTaDTLxxyLi1mDUBVqWJamID&index=6>

3. Concluding Exercise 收功

<https://www.youtube.com/watch?v=kt-QSno0-dI&list=PL6e3KTUbluTaDTLxxyLi1mDUBVqWJamID&index=5>

1. **Dian Bu Gong (Step Touch Method)**
2. Preparatory exercise预备功

<https://www.youtube.com/watch?v=Gk28UoO9gzo&list=PL6e3KTUbluTaDTLxxyLi1mDUBVqWJamID&index=7>

1. Step Touch Exercise

<https://www.youtube.com/watch?v=IOX5eG8HW0k&list=PL6e3KTUbluTaDTLxxyLi1mDUBVqWJamID&index=1>

1. Concluding Exercise 收功

<https://www.youtube.com/watch?v=kt-QSno0-dI&list=PL6e3KTUbluTaDTLxxyLi1mDUBVqWJamID&index=5>

**Supplementary Material 2 Video links of Guolin Qigong training**

1. **Ascending, descending, opening and closing Method (Sheng Jiang Kai He Fa)**
2. Preparatory exercise预备功

<https://www.youtube.com/watch?v=Gk28UoO9gzo&list=PL6e3KTUbluTaDTLxxyLi1mDUBVqWJamID&index=7>

1. Sheng Jiang kai He Exercise

<https://www.youtube.com/watch?v=GSzPoMe7nCk&list=PLKGDHIdJWjmhLjJCJQfbqkrTCCojNZglK&index=14>

1. Concluding Exercise 收功

<https://www.youtube.com/watch?v=kt-QSno0-dI&list=PL6e3KTUbluTaDTLxxyLi1mDUBVqWJamID&index=5>
