# Supplementary material for: Exploring Guolin Qigong (Mind-Body Exercise) for Improving Cancer Related Fatigue in Cancer Survivors: A Mixed Method Randomized Controlled Trial Protocol
Source: Integr Cancer Ther. 2024 May 17;23:15347354241252698. doi: 10.1177/15347354241252698 (PMC11102686; doi:10.1177/15347354241252698)
Supplement: sj-docx-4-ict-10.1177_15347354241252698 – Supplemental material for Exploring Guolin Qigong (Mind-Body Exercise) for Improving Cancer Related Fatigue in Cancer Survivors: A Mixed Method Randomized Controlled Trial Protocol [file sj-docx-4-ict-10.1177_15347354241252698.docx]

**Supplementary Material 4 Semi-Structured Interview Questions**

1) How do you feel about the 12 weeks of Guolin Qigong study? E.g. Physical, psychological or mental changes

1. Have you had other benefits from this Guolin Qigong study? E.g. Energy level, attention, emotional state, relationship, work productivity

For every item: Changes, frequency

1. What did you like about this Qigong program? E.g. Shared worry and support, meeting others with the same worries, instructor
2. What are the difficulties you face in adhering to this Qigong program during the 12 week period?
3. What could be improved? E.g. Logistic, duration and frequency of face to face and home practice, cost of travelling etc
4. Would you recommend Guolin Qigong to other cancer survivors? Why? Why not?
