# Supplementary material for: Exploring Guolin Qigong (Mind-Body Exercise) for Improving Cancer Related Fatigue in Cancer Survivors: A Mixed Method Randomized Controlled Trial Protocol
Source: Integr Cancer Ther. 2024 May 17;23:15347354241252698. doi: 10.1177/15347354241252698 (PMC11102686; doi:10.1177/15347354241252698)
Supplement: sj-pdf-1-ict-10.1177_15347354241252698 – Supplemental material for Exploring Guolin Qigong (Mind-Body Exercise) for Improving Cancer Related Fatigue in Cancer Survivors: A Mixed Method Randomized Controlled Trial Protocol [file sj-pdf-1-ict-10.1177_15347354241252698.pdf]

SPIRIT 2013 explanation and elaboration: guidance for protocols of clinical trials

An-Wen Chan,[1] Jennifer M Tetzlaff,[2] Peter C Gøtzsche,[3] Douglas G Altman,[4] Howard Mann,[5] Jesse A Berlin,[6] Kay Dickersin,[7] Asbjørn Hróbjartsson,[3] Kenneth F Schulz,[8] Wendy R Parulekar,[9] Karmela Krleža-Jeric,[10] Andreas Laupacis,[11] David Moher[2 10]

[1]Women's College Research Institute at Women's College Hospital, Department of Medicine, University of Toronto, Toronto, Canada, M5G 1N8
[2]Ottawa Methods Centre, Clinical Epidemiology Program, Ottawa Hospital Research Institute, Ottawa, Canada
[3]Nordic Cochrane Centre, Rigshospitalet, Copenhagen, Denmark
[4]Centre for Statistics in Medicine, University of Oxford, Oxford, UK
[5]Division of Medical Ethics and Humanities, University of Utah School of Medicine, Salt Lake City, USA
[6]Janssen Research and Development, Titusville, USA
[7]Center for Clinical Trials, Johns Hopkins Bloomberg School of Public Health, Baltimore, USA
[8]Quantitative Sciences, FHI 360, Research Triangle Park, USA
[9]NCIC Clinical Trials Group, Cancer Research Institute, Queen's University, Kingston, Canada
[10]Department of Epidemiology and Community Medicine, University of Ottawa, Ottawa, Canada
[11]Keenan Research Centre at the Li Ka Shing Knowledge Institute of St Michael's Hospital, Faculty of Medicine, University of Toronto, Toronto, Canada
Correspondence to: A-W Chan anwen.chan@utoronto.ca
Accepted: 04 October 2012
Cite this as: *BMJ* 2013;346:e7586
doi: 10.1136/bmj.e7586

High quality protocols facilitate proper conduct, reporting, and external review of clinical trials. However, the completeness of trial protocols is often inadequate. To help improve the content and quality of protocols, an international group of stakeholders developed the SPIRIT 2013 Statement (Standard Protocol Items: Recommendations for Interventional Trials). The SPIRIT Statement provides guidance in the form of a checklist of recommended items to include in a clinical trial protocol.

This SPIRIT 2013 Explanation and Elaboration paper provides important information to promote full understanding of the checklist recommendations. For each checklist item, we provide a rationale and detailed description; a model example from an actual protocol; and relevant references supporting its importance. We strongly recommend that this explanatory paper be used in conjunction with the SPIRIT Statement. A website of resources is also available (www.spirit-statement.org).

The SPIRIT 2013 Explanation and Elaboration paper, together with the Statement, should help with the drafting of trial protocols. Complete documentation of key trial elements can facilitate transparency and protocol review for the benefit of all stakeholders.

Every clinical trial should be based on a protocol—a document that details the study rationale, proposed methods, organisation, and ethical considerations.[1] Trial investigators and staff use protocols to document plans for study conduct at all stages from participant recruitment to results dissemination. Funding agencies, research ethics committees/institutional review boards, regulatory agencies, medical journals, systematic reviewers, and other groups rely on protocols to appraise the conduct and reporting of clinical trials.

To meet the needs of these diverse stakeholders, protocols should adequately address key trial elements. However, protocols often lack information on important concepts relating to study design and dissemination plans.[2-12] Guidelines for writing protocols can help improve their completeness, but existing guidelines vary extensively in their content and have limitations, including non-systematic methods of development, limited stakeholder involvement, and lack of citation of empirical evidence to support their recommendations.[13] As a result, there is also variation in the precise definition and scope of a trial protocol, particularly in terms of its relation to other documents such as procedure manuals.[14]

Given the importance of trial protocols, an international group of stakeholders launched the SPIRIT (Standard Protocol Items: Recommendations for Interventional Trials) Initiative in 2007 with the primary aim of improving the content of trial protocols. The main outputs are the SPIRIT 2013 Statement,[14] consisting of a 33 item checklist of minimum recommended protocol items (table 1) plus a diagram (fig1); and this accompanying Explanation and Elaboration (E&E) paper. Additional information and resources are also available on the SPIRIT website (www.spirit-statement.org).

The SPIRIT 2013 Statement and E&E paper reflect the collaboration and input of 115 contributors, including trial investigators, healthcare professionals, methodologists, statisticians, trial coordinators, journal editors, as well as representatives from research ethics committees, industry and non-industry funders, and regulatory agencies. Details of the scope and methods have been published elsewhere.[13-15] Briefly, three complementary methods were specified beforehand, in line with current recommendations for development of reporting guidelines[16]: 1) a Delphi consensus survey[15]; 2) two systematic reviews to identify existing protocol guidelines and empirical evidence supporting the importance of specific checklist items; and 3) two face-to-face consensus meetings to finalise the SPIRIT 2013 checklist. Furthermore, the checklist was pilot tested by graduate course students, and an implementation strategy was developed at a stakeholder meeting.

The SPIRIT recommendations are intended as a guide for those preparing the full protocol for a clinical trial. A clinical trial is a prospective study in which one or more

chaa006386.indd 1 31/01/2013 10:33:35

	STUDY PERIOD							
	Enrolment	Allocation	Post-allocation					Close-out
TIMEPOINT*	$-t_1$	0	t_1	t_2	t_3	t_4	*etc*	t_x
ENROLMENT:								
Eligibility screen	X							
Informed consent	X							
(List other procedures)	X							
Allocation		X						
INTERVENTIONS:								
(Intervention A)			◆——	——	——◆			
(Intervention B)			X		X			
(List other study groups)			◆——	——	——	——◆		
ASSESSMENTS:								
(List baseline variables)	X	X						
(List outcome variables)				X		X	etc	X
(List other data variables)			X	X	X	X	etc	X

* List specific timepoints in this row

Fig 1 | Example template for the schedule of enrolment, interventions, and assessments (recommended content can be displayed using other schematic formats). This template is copyrighted by the SPIRIT Group and is reproduced by BMJ with their permission.

interventions are assigned to human participants in order to assess the effects on health related outcomes. The recommendations are not intended to prescribe how a trial should be designed or conducted. Rather, we call for a transparent and complete description of what is intended, regardless of the characteristics or quality of the plans. The SPIRIT 2013 Statement addresses the minimum content for interventional trials; additional concepts may be important to describe in protocols for trials of specific designs (eg, crossover trials) or in protocols intended for submission to specific groups (eg, funders, research ethics committees/institutional review boards). If information for a recommended item is not yet available when the protocol is being finalised (eg, funding sources), this should be explicitly stated and the protocol updated as new information is obtained. Formatting conventions such as a table of contents, glossary of non-standard or ambiguous terms (eg, randomisation phase or off-protocol), and list of abbreviations and references will facilitate understanding of the protocol.

Purpose and development of explanation and elaboration paper

Modelled after other reporting guidelines,[17 18] this E&E paper presents each checklist item with at least one model example from an actual protocol, followed by a full explanation of the rationale and main issues to address. This E&E paper provides important information to facilitate full understanding of each checklist item, and is intended to be used in conjunction with the SPIRIT 2013 Statement.[14] These complementary tools serve to inform trial investigators about important issues to consider in the protocol as they relate to trial design, conduct, reporting, and organisation.

To identify examples for each checklist item, we obtained protocols from public websites, journals, trial investigators, and industry sponsors. Model examples were selected to reflect how key elements could be appropriately described in a trial protocol. Some examples illustrate a specific component of a checklist item, while others encompass all key recommendations for an item. Additional examples are also available on the SPIRIT website (www.spirit-statement.org). The availability of examples for all checklist items indicates the feasibility of addressing each recommended item in the main protocol rather than in separate documents.

Examples are quoted verbatim from the trial protocol. Proper names of trial personnel have been abbreviated with italicised initials, and any reference numbers cited in the original quoted text are denoted by [*Reference*] to distinguish them from references cited in this E&E paper.

For each checklist item we also strived to provide references to empirical data supporting its relevance, which we identified through a systematic review conducted to inform the content of the SPIRIT checklist. We searched MEDLINE, the Cochrane Methodology Register, and the Cochrane Database of Systematic Reviews (limited to methodology reviews) up to September 2009, and EMBASE up to August 2007. We searched reference lists, PubMed "related articles," and citation searches using SCOPUS to identify additional relevant studies. We used piloted forms to screen and extract data relevant to specific checklist items.

Studies were included if they provided empirical data to support or refute the importance of a given protocol concept. A summary of the relevant methodological articles was provided to each E&E author for use in preparing the initial draft text for up to six checklist items; each draft was also reviewed and revised by a second author. When citing empirical evidence in the E&E, we aimed to reference a systematic review when available. When no review was identified, we either cited all relevant individual studies, or if too numerous, a representative sample of the literature. Some items had little or no identified empirical evidence (eg, title) but their inclusion in the checklist is supported by a strong pragmatic or ethical rationale. Where relevant, we also provide references to non-empirical publications for further reading.

Two lead authors (AWC, JMT) collated and refined the content and format for all items, and then circulated three iterations of an overall draft to the coauthors for editing and final approval.

SPIRIT 2013 Explanation and Elaboration

Section 1: Administrative information

Item 1: Descriptive title identifying the study design, population, interventions, and, if applicable, trial acronym

Example

"A multi-center, investigator-blinded, randomized, 12-month, parallel-group, non-inferiority study to compare the efficacy of 1.6 to 2.4 g Asacol® Therapy QD [once daily] versus divided dose (BID [twice daily]) in the maintenance of remission of ulcerative colitis."[19]

Explanation

The title provides an important means of trial identification. A succinct description that conveys the topic (study population, interventions), acronym (if any), and basic study design—including the method of intervention allocation (eg, parallel group randomised trial; single-group trial)—will facilitate retrieval from literature or internet

chaa006386.indd 2 31/01/2013 10:33:36

Table 1 | SPIRIT 2013 checklist: recommended items to address in a clinical trial protocol and related documents*

Section/item	ItemNo	Description
**Administrative information**		
Title	1	Descriptive title identifying the study design, population, interventions, and, if applicable, trial acronym
Trial registration	2a	Trial identifier and registry name. If not yet registered, name of intended registry
	2b	All items from the World Health Organization Trial Registration Data Set
Protocol version	3	Date and version identifier
Funding	4	Sources and types of financial, material, and other support
Roles and responsibilities	5a	Names, affiliations, and roles of protocol contributors
	5b	Name and contact information for the trial sponsor
	5c	Role of study sponsor and funders, if any, in study design; collection, management, analysis, and interpretation of data; writing of the report; and the decision to submit the report for publication, including whether they will have ultimate authority over any of these activities
	5d	Composition, roles, and responsibilities of the coordinating centre, steering committee, endpoint adjudication committee, data management team, and other individuals or groups overseeing the trial, if applicable (see Item 21a for data monitoring committee)
**Introduction**		
Background and rationale	6a	Description of research question and justification for undertaking the trial, including summary of relevant studies (published and unpublished) examining benefits and harms for each intervention
	6b	Explanation for choice of comparators
Objectives	7	Specific objectives or hypotheses
Trial design	8	Description of trial design including type of trial (eg, parallel group, crossover, factorial, single group), allocation ratio, and framework (eg, superiority, equivalence, noninferiority, exploratory)
**Methods: Participants, interventions, and outcomes**		
Study setting	9	Description of study settings (eg, community clinic, academic hospital) and list of countries where data will be collected. Reference to where list of study sites can be obtained
Eligibility criteria	10	Inclusion and exclusion criteria for participants. If applicable, eligibility criteria for study centres and individuals who will perform the interventions (eg, surgeons, psychotherapists)
Interventions	11a	Interventions for each group with sufficient detail to allow replication, including how and when they will be administered
	11b	Criteria for discontinuing or modifying allocated interventions for a given trial participant (eg, drug dose change in response to harms, participant request, or improving/worsening disease)
	11c	Strategies to improve adherence to intervention protocols, and any procedures for monitoring adherence (eg, drug tablet return, laboratory tests)
	11d	Relevant concomitant care and interventions that are permitted or prohibited during the trial
Outcomes	12	Primary, secondary, and other outcomes, including the specific measurement variable (eg, systolic blood pressure), analysis metric (eg, change from baseline, final value, time to event), method of aggregation (eg, median, proportion), and time point for each outcome. Explanation of the clinical relevance of chosen efficacy and harm outcomes is strongly recommended
Participant timeline	13	Time schedule of enrolment, interventions (including any run-ins and washouts), assessments, and visits for participants. A schematic diagram is highly recommended (see fig 1)
Sample size	14	Estimated number of participants needed to achieve study objectives and how it was determined, including clinical and statistical assumptions supporting any sample size calculations
Recruitment	15	Strategies for achieving adequate participant enrolment to reach target sample size
**Methods: Assignment of interventions (for controlled trials)**		
Allocation:		
Sequence generation	16a	Method of generating the allocation sequence (eg, computer-generated random numbers), and list of any factors for stratification. To reduce predictability of a random sequence, details of any planned restriction (eg, blocking) should be provided in a separate document that is unavailable to those who enrol participants or assign interventions
Allocation concealment mechanism	16b	Mechanism of implementing the allocation sequence (eg, central telephone; sequentially numbered, opaque, sealed envelopes), describing any steps to conceal the sequence until interventions are assigned
Implementation	16c	Who will generate the allocation sequence, who will enrol participants, and who will assign participants to interventions
Blinding (masking)	17a	Who will be blinded after assignment to interventions (eg, trial participants, care providers, outcome assessors, data analysts) and how
	17b	If blinded, circumstances under which unblinding is permissible and procedure for revealing a participant's allocated intervention during the trial
**Methods: Data collection, management, and analysis**		
Data collection methods	18a	Plans for assessment and collection of outcome, baseline, and other trial data, including any related processes to promote data quality (eg, duplicate measurements, training of assessors) and a description of study instruments (eg, questionnaires, laboratory tests) along with their reliability and validity, if known. Reference to where data collection forms can be found, if not in the protocol
	18b	Plans to promote participant retention and complete follow-up, including list of any outcome data to be collected for participants who discontinue or deviate from intervention protocols
Data management	19	Plans for data entry, coding, security, and storage, including any related processes to promote data quality (eg, double data entry; range checks for data values). Reference to where details of data management procedures can be found, if not in the protocol
Statistical methods	20a	Statistical methods for analysing primary and secondary outcomes. Reference to where other details of the statistical analysis plan can be found, if not in the protocol
	20b	Methods for any additional analyses (eg, subgroup and adjusted analyses)
	20c	Definition of analysis population relating to protocol non-adherence (eg, as randomised analysis), and any statistical methods to handle missing data (eg, multiple imputation)
**Methods: Monitoring**		
Data monitoring	21a	Composition of data monitoring committee (DMC); summary of its role and reporting structure; statement of whether it is independent from the sponsor and competing interests; and reference to where further details about its charter can be found, if not in the protocol. Alternatively, an explanation of why a DMC is not needed

Contined

chaa006386.indd 3 31/01/2013 10:33:37

Section/item	ItemNo	Description
	21b	Description of any interim analyses and stopping guidelines, including who will have access to these interim results and make the final decision to terminate the trial
Harms	22	Plans for collecting, assessing, reporting, and managing solicited and spontaneously reported adverse events and other unintended effects of trial interventions or trial conduct
Auditing	23	Frequency and procedures for auditing trial conduct, if any, and whether the process will be independent from investigators and the sponsor
**Ethics and dissemination**		
Research ethics approval	24	Plans for seeking research ethics committee/institutional review board (REC/IRB) approval
Protocol amendments	25	Plans for communicating important protocol modifications (eg, changes to eligibility criteria, outcomes, analyses) to relevant parties (eg, investigators, REC/IRBs, trial participants, trial registries, journals, regulators)
Consent or assent	26a	Who will obtain informed consent or assent from potential trial participants or authorised surrogates, and how (see Item 32)
	26b	Additional consent provisions for collection and use of participant data and biological specimens in ancillary studies, if applicable
Confidentiality	27	How personal information about potential and enrolled participants will be collected, shared, and maintained in order to protect confidentiality before, during, and after the trial
Declaration of interests	28	Financial and other competing interests for principal investigators for the overall trial and each study site
Access to data	29	Statement of who will have access to the final trial dataset, and disclosure of contractual agreements that limit such access for investigators
Ancillary and post-trial care	30	Provisions, if any, for ancillary and post-trial care, and for compensation to those who suffer harm from trial participation
Dissemination policy	31a	Plans for investigators and sponsor to communicate trial results to participants, healthcare professionals, the public, and other relevant groups (eg, via publication, reporting in results databases, or other data sharing arrangements), including any publication restrictions
	31b	Authorship eligibility guidelines and any intended use of professional writers
	31c	Plans, if any, for granting public access to the full protocol, participant-level dataset, and statistical code
**Appendices**		
Informed consent materials	32	Model consent form and other related documentation given to participants and authorised surrogates
Biological specimens	33	Plans for collection, laboratory evaluation, and storage of biological specimens for genetic or molecular analysis in the current trial and for future use in ancillary studies, if applicable

*Amendments to the protocol should be tracked and dated. The SPIRIT checklist belongs to the SPIRIT Group and is reproduced by BMJ with their permission

searches and rapid judgment of relevance.[20] It can also be helpful to include the trial framework (eg, superiority, non-inferiority), study objective or primary outcome, and if relevant, the study phase (eg, phase II).

Trial registration—registry

Item 2a: Trial identifier and registry name. If not yet registered, name of intended registry

Example

"EudraCT: 2010-019180-10
ClinicalTrials.gov: NCT01066572
ISRCTN: 54540667."[21]

Explanation

There are compelling ethical and scientific reasons for trial registration.[22-24] Documentation of a trial's existence on a publicly accessible registry can help to increase transparency,[24 25] decrease unnecessary duplication of research effort, facilitate identification of ongoing trials for prospective participants, and identify selective reporting of study results.[26-28] As mandated by the International Committee of Medical Journal Editors (ICMJE) and jurisdictional legislation,[29-31] registration of clinical trials should occur before recruitment of the first trial participant.

We recommend that registry names and trial identifiers assigned by the registries be prominently placed in the protocol, such as on the cover page. If the trial is not yet registered, the intended registry should be indicated and the protocol updated upon registration. When registration in multiple registries is required (eg, to meet local regulation), each identifier should be clearly listed in the protocol and each registry.

Trial registration—data set

Item 2b: All items from the World Health Organization Trial Registration Data Set

Example: see table 2

Explanation

In addition to a trial registration number, the World Health Organization (WHO) recommends a minimum standard list of items to be included in a trial registry in order for a trial to be considered fully registered (www.who.int/ictrp/network/trds/en/index.html). These standards are supported by ICMJE, other journal editors, and jurisdictional legislation.[29-31] We recommend that the WHO Trial Registration Data Set be included in the protocol to serve as a brief structured summary of the trial. Its inclusion in the protocol can also signal updates for the registry when associated protocol sections are amended—thereby promoting consistency between information in the protocol and registry.

Protocol version

Item 3: Date and version identifier

Example

"Issue date: 25 Jul 2005
Protocol amendment number: 05
Authors: *MD, JH*

Revision chronology:

UM . . . 00, 2004-Jan-30 Original

UM . . . 01, 2004-Feb-7 Amendment 01.:
Primary reason for amendment: changes in Section 7.1 regarding composition of comparator placebo
Additional changes (these changes in and of themselves would not justify a protocol amendment): correction of typographical error in Section 3.3 . . .

UM . . . 05, 2005-Jul-25 Amendment No.5:
At the request of US FDA statements were added to the protocol to better clarify and define the algorithm for determining clinical or microbiological failures prior to the follow-up visit."[33]

Explanation

Sequentially labelling and dating each protocol version helps to mitigate potential confusion over which document

Table 2 | Example of trial registration data

Data category	Information[32]
Primary registry and trial identifying number	ClinicalTrials.gov NCT01143272
Date of registration in primary registry	11 June, 2010
Secondary identifying numbers	BNI-2009-01, 2009-017374-20, ISRCTN01005546, DRKS00000084
Source(s) of monetary or material support	Bernhard Nocht Institute for Tropical Medicine
Primary sponsor	Bernhard Nocht Institute for Tropical Medicine
Secondary sponsor(s)	German Federal Ministry of Education and Research
Contact for public queries	SE, MD, MPH [email address]
Contact for scientific queries	SE, MD, MPH Bernhard Nocht Institute for Tropical Medicine, Hamburg, Germany
Public title	Probiotic *Saccharomyces boulardii* for the prevention of antibiotic associated diarrhoea (SacBo)
Scientific title	*S boulardii* for the prevention of antibiotic associated diarrhoea—randomised, double blind, placebo controlled trial
Countries of recruitment	Germany
Health condition(s) or problem(s) studied	Antibiotic treatment, *Clostridium difficile*, diarrhoea
Intervention(s)	Active comparator: *S boulardii* (500 mg *S boulardii* per day)
	Placebo comparator: microcristallin cellulose (matching capsules containing no active ingredients)
Key inclusion and exclusion criteria	Ages eligible for study: ≥18 years; Sexes eligible for study: both; Accepts healthy volunteers: no
	Inclusion criteria: adult patient (≥ 18 years), patient hospitalised . . .
	Exclusion criteria: allergy against yeast and/or Perenterol forte and/or placebos containing *S cerevisiae* HANSEN CBS 5926, lactose monohydrate, magnesium stearate, gelatine, sodium dodecyl sulfate, titan dioxide, microcrystalline cellulose
Study type	Interventional
	Allocation: randomized; Intervention model: parallel assignment; Masking: double blind . . .
	Primary purpose: prevention
	Phase III
Date of first enrolment	June 2010
Target sample size	1520
Recruitment status	Recruiting
Primary outcome(s)	Cumulative incidence of any antibiotic associated diarrhoea (time frame: 2 years; not designated as safety issue)
Key secondary outcomes	Cumulative incidence of *C difficile* associated diarrhoea (time frame: 2 years; not designated as safety issue) . . .

is the most recent. Explicitly listing the changes made relative to the previous protocol version is also important (see Item 25). Transparent tracking of versions and amendments facilitates trial conduct, review, and oversight.

Funding

Item 4: Sources and types of financial, material, and other support

Example

"Tranexamic acid will be manufactured by Pharmacia (Pfizer, Sandwich, UK) and placebo by South Devon Healthcare NHS Trust, UK. The treatment packs will be prepared by an independent clinical trial supply company (Brecon Pharmaceuticals Limited, Hereford, UK) . . .
LSHTM [London School of Hygiene and Tropical Medicine] is funding the run-in costs for the WOMAN trial and up to 2,000 patients' recruitment. The main phase is funded by the UK Department of Health and the Wellcome Trust. Funding for this trial covers meetings and central organisational costs only. Pfizer, the manufacturer of tranexamic acid, have provided the funding for the trial drug and placebo used for this trial. An educational grant, equipment and consumables for ROTEM [thromboelastometry procedure] analysis has been provided by Tem Innovations GmbH, M.-Kollar-Str. 13-15, 81829 Munich, Germany for use in the WOMAN-ETAC study. An application for funding to support local organisational costs has been made to University of Ibadan Senate Research Grant. The design, management, analysis and reporting of the study are entirely independent of the manufacturers of tranexamic acid and Tem Innovations GmbH."[34]

Explanation

A description of the sources of financial and non-financial support provides relevant information to assess study feasibility and potential competing interests (Item 28). Although both industry funded and non-industry funded trials are susceptible to bias,[4 35] the former are more likely to report trial results and conclusions that favour their own interventions.[27 36-39] This tendency could be due to industry trials being more likely to select effective interventions for evaluation (Item 6a), to use less effective control interventions (Item 6b), or to selectively report outcomes (Item 12), analyses (Item 20) or full studies (Item 31).[38 40-43] Non-financial support (eg, provision of drugs) from industry has not been shown to be associated with biased results, although few studies have examined this issue.[44 45]

At a minimum, the protocol should identify the sources of financial and non-financial support; the specific type (eg, funds, equipment, drugs, services) and time period of support; and any vested interest that the funder may have in the trial. If a trial is not yet funded when the protocol is first written, the proposed sources of support should be listed and updated as funders are confirmed.

No clear consensus exists regarding the level of additional funding details that should be provided in the trial protocol as opposed to trial contracts, although full disclosure of funding information in the protocol can help to better identify financial competing interests. Some jurisdictional guidelines require more detailed disclosure, including monetary amounts granted from each funder, the mechanism of providing financial support (eg, paid in fixed sum or per recruited participant), and the specific fund recipient (eg, trial investigator, department/institute).[46] Detailed disclosure allows research ethics committees/institutional review boards (REC/IRBs) to assess whether the reimbursement amount is reasonable in relation to the time and expenses incurred for trial conduct.

chaa006386.indd 5 31/01/2013 10:33:37

Roles and responsibilities—contributorship

Item 5a: Names, affiliations, and roles of protocol contributors

Example

"*RTL* [address], *EJM* [address], *AK* [address] . . .

Authors' contributions

RTL conceived of the study. *AK*, *EN*, *SB*, *PR*, *WJ*, *JH*, and *MC* initiated the study design and *JK* and *LG* helped with implementation. *RTL*, *JK*, *LG*, and *FP* are grant holders. *LT* and *EM* provided statistical expertise in clinical trial design and *RN* is conducting the primary statistical analysis. All authors contributed to refinement of the study protocol and approved the final manuscript."[47]

Explanation

Individuals who contribute substantively to protocol development and drafting should have their contributions reported. As with authorship of journal articles,[48] listing the protocol contributors, their affiliations, and their roles in the protocol development process provides due recognition, accountability, and transparency. Naming of contributors can also help to identify competing interests and reduce ghost authorship (Items 28 and 31b).[9 10] If professional medical writers are employed to draft the protocol, then this should be acknowledged as well.

Naming of authors and statements of contributorship are standard for protocols published in journals such as *Trials*[49] but are uncommon for unpublished protocols. Only five of 44 industry-initiated protocols approved in 1994-95 by a Danish research ethics committee explicitly identified the protocol authors.[9]

Roles and responsibilities—sponsor contact information

Item 5b: Name and contact information for the trial sponsor

Example

"Trial Sponsor: University of Nottingham

Sponsor's Reference: RIS 8024 . . .

Contact name: Mr *PC*

Address: King's Meadow Campus . . .

Telephone: . . .

Email: . . ."[50]

Explanation

The sponsor can be defined as the individual, company, institution, or organisation assuming overall responsibility for the initiation and management of the trial, and is not necessarily the main funder.[51 52] In general, the company is the sponsor in industry initiated trials, while the funding agency or institution of the principal investigator is often the sponsor for investigator initiated trials. For some investigator initiated trials, the principal investigator can be considered to be a "sponsor-investigator" who assumes both sponsor and investigator roles.[51 53]

Identification of the trial sponsor provides transparency and accountability. The protocol should identify the name, contact information, and if applicable, the regulatory agency identifying number of the sponsor.

Roles and responsibilities—sponsor and funder

Item 5c: Role of study sponsor and funders, if any, in study design; collection, management, analysis, and interpretation of data; writing of the report; and the decision to submit the report for publication, including whether they will have ultimate authority over any of these activities

Example

"This funding source had no role in the design of this study and will not have any role during its execution, analyses, interpretation of the data, or decision to submit results."[54]

Explanation

There is potential for bias when the trial sponsor or funder (sometimes the same entity) has competing interests (Item 28) and substantial influence on the planning, conduct, or reporting of a trial. Empirical research indicates that specific forms of bias tend to be more prevalent in trials funded by industry compared to those funded by non-commercial sources.[36-38 45 55-60] The design, analysis, interpretation, and reporting of most industry-initiated trials are controlled by the sponsor; this authority is often enforced by contractual agreements signed between the sponsor and trial investigators (Item 29).[10 61]

The protocol should explicitly outline the roles and responsibilities of the sponsor and any funders in study design, conduct, data analysis and interpretation, manuscript writing, and dissemination of results. It is also important to state whether the sponsor or funder controls the final decision regarding any of these aspects of the trial.

Despite the importance of declaring the roles of the trial sponsor and funders, few protocols explicitly do so. Among 44 protocols for industry-initiated trials receiving ethics approval in Denmark from 1994-95, none stated explicitly who had contributed to the design of the trial.[9]

Roles and responsibilities—committees

Item 5d: Composition, roles, and responsibilities of the coordinating centre, steering committee, endpoint adjudication committee, data management team, and other individuals or groups overseeing the trial, if applicable (see Item 21a for data monitoring committee)

Explanation

The protocol should outline the general membership of the various committees or groups involved in trial coordination and conduct; describe the roles and responsibilities of each; and (when known) identify the chairs and members. This information helps to ensure that roles and responsibilities are clearly understood at the trial onset, and facilitates communication from external parties regarding the trial. It also enables readers to understand the mandate and expertise of those responsible for overseeing participant safety, study design, database integrity, and study conduct. For example, empirical evidence supports the pivotal role of an epidemiologist or biostatistician in designing and conducting higher quality trials.[63 64]

Example

"Principal investigator and research physician

Design and conduct of RITUXVAS

Preparation of protocol and revisions

Preparation of investigators brochure (IB) and CRFs [case report forms]

Organising steering committee meetings

Managing CTO [clinical trials office]

Publication of study reports

Members of TMC [Trial Management Committee]

Steering committee (SC)

(see title page for members)

Agreement of final protocol

All lead investigators will be steering committee members. One lead investigator per country will be nominated as national coordinator.

Recruitment of patients and liaising with principle [sic] investigator

Reviewing progress of study and if necessary agreeing changes to the protocol and/or investigators brochure to facilitate the smooth running of the study.

Trial management committee (TMC)

(Principle [sic] investigator, research physician, administrator)

Study planning

Organisation of steering committee meetings

Provide annual risk report MHRA [Medicines and Healthcare Products Regulatory Agency] and ethics committee

SUSAR [Serious unexpected suspected adverse events] reporting to MHRA and Roche

Responsible for trial master file

Budget administration and contractual issues with individual centres

Advice for lead investigators

Audit of 6 monthly feedback forms and decide when site visit to occur.

Assistance with international review, board/independent ethics committee applications

Data verification

Randomisation

Organisation of central serum sample collection

Data manager

Maintenance of trial IT system and data entry

Data verification

Lead investigators

In each participating centre a lead investigator (senior nephrologist/rheumatologist/ immunologist) will be identified, to be responsible for identification, recruitment, data collection and completion of CRFs, along with follow up of study patients and adherence to study protocol and investigators brochure. . . . Lead investigators will be steering committee members, with one investigator per country being nominated as national coordinator."[62]

Section 2: Introduction

Background and rationale

Item 6a: Description of research question and justification for undertaking the trial, including summary of relevant studies (published and unpublished) examining benefits and harms for each intervention

Explanation

The value of a research question, as well as the ethical and scientific justification for a trial, depend to a large degree on the uncertainty of the comparative benefits or harms of the interventions, which depends in turn on the existing body of knowledge on the topic. The background section of a protocol should summarise the importance of the research question, justify the need for the trial in the context of available evidence, and present any available data regarding the potential effects of the interventions (efficacy and harms).[66 67] This information is particularly important to the trial participants and personnel, as it provides motivation for contributing to the trial.[68 69] It is also relevant to funders, REC/IRBs, and other stakeholders who evaluate the scientific and ethical basis for trial conduct.

To place the trial in the context of available evidence, it is strongly recommended that an up-to-date systematic review of relevant studies be summarised and cited in the protocol.[70] Several funders request this information in grant applications.[71 72] Failure to review the cumulated evidence can lead to unnecessary duplication of research or to trial participants being deprived of effective, or exposed to harmful, interventions.[73-76] A minority of published trial reports cite a systematic review of pre-existing evidence,[77 78] and in one survey only half of trial investigators

Example

"Background

Introduction: For people at ages 5 to 45 years, trauma is second only to HIV/AIDS as a cause of death. . . .

Mechanisms: The haemostatic system helps to maintain the integrity of the circulatory system after severe vascular injury, whether traumatic or surgical in origin.[reference] Major surgery and trauma trigger similar haemostatic responses . . . Antifibrinolytic agents have been shown to reduce blood loss in patients with both normal and exaggerated fibrinolytic responses to surgery, and do so without apparently increasing the risk of post-operative complications, . . .

Existing knowledge: Systemic antifibrinolytic agents are widely used in major surgery to prevent fibrinolysis and thus reduce surgical blood loss. A recent systematic review [reference] of randomised controlled trials of antifibrinolytic agents (mainly aprotinin or tranexamic acid) in elective surgical patients identified 89 trials including 8,580 randomised patients (74 trials in cardiac, eight in orthopaedic, four in liver, and three in vascular surgery). The results showed that these treatments reduced the numbers needing transfusion by one third, reduced the volume needed per transfusion by one unit, and halved the need for further surgery to control bleeding. These differences were all highly statistically significant. There was also a statistically non-significant reduction in the risk of death (RR=0.85: 95% CI 0.63 to 1.14) in the antifibrinolytic treated group.

. . .

Need for a trial: A simple and widely practicable treatment that reduces blood loss following trauma might prevent thousands of premature trauma deaths each year and secondly could reduce exposure to the risks of blood transfusion. Blood is a scarce and expensive resource and major concerns remain about the risk of transfusion-transmitted infection. . . . A large randomised trial is therefore needed of the use of a simple, inexpensive, widely practicable antifibrinolytic treatment such as tranexamic acid . . . in a wide range of trauma patients who, when they reach hospital are thought to be at risk of major haemorrhage that could significantly affect their chances of survival.

Dose selection

The systematic review of randomised controlled trials of antifibrinolytic agents in surgery showed that dose regimens of tranexamic acid vary widely.[reference] . . .

In this emergency situation, administration of a fixed dose would be more practicable as determining the weight of a patient would be impossible. Therefore a fixed dose within the dose range which has been shown to inhibit fibrinolysis and provide haemostatic benefit is being used for this trial. . . . The planned duration of administration allows for the full effect of tranexamic acid on the immediate risk of haemorrhage without extending too far into the acute phase response seen after surgery and trauma."[65]

chaa006386.indd 7 31/01/2013 10:33:37

were aware of a relevant existing review when they had designed their trial.[79] Given that about half of trials remain unpublished,[80-82] and that published trials often represent a biased subset of all trials,[80 83] it is important that systematic reviews include a search of online resources such as trial registries, results databases, and regulatory agency websites.[84]

Background and rationale—choice of comparators

Item 6b: Explanation for choice of comparators

> **Example**
>
> **"Choice of comparator**
>
> In spite of the increasing numbers of resistant strains, chloroquine monotherapy is still recommended as standard blood-stage therapy for patients with *P* [*Plasmodium*] *vivax* malaria in the countries in which this trial will be conducted. Its selection as comparator is therefore justified. The adult dose of chloroquine will be 620 mg for 2 days followed by 310 mg on the third day and for children 10 mg/kg for the first two days and 5 mg/kg for the third day. Total dose is in accordance with the current practice in the countries where the study is conducted. The safety profile of chloroquine is well established and known. Although generally well tolerated, the following side-effects of chloroquine treatment have been described: Gastro-intestinal disturbances, headache, hypotension, convulsions, visual disturbances, depigmentation or loss of hair, skin reactions (rashes, pruritus) and, rarely, bone-marrow suppression and hypersensitivity reactions such as urticaria and angioedema. Their occurrence during the present trial may however be unlikely given the short (3-day) duration of treatment."[85]

Explanation

The choice of control interventions has important implications for trial ethics, recruitment, results, and interpretation. In trials comparing an intervention to an active control or usual care, a clear description of the rationale for the comparator intervention will facilitate understanding of its appropriateness.[86 87] For example, a trial in which the control group receives an inappropriately low dose of an active drug will overestimate the relative efficacy of the study intervention in clinical practice; conversely, an inappropriately high dose in the control group will lead to an underestimate of the relative harms of the study intervention.[87 88]

The appropriateness of using placebo-only control groups has been the subject of extensive debate and merits careful consideration of the existence of other effective treatments, the potential risks to trial participants, and the need for assay sensitivity—that is, ability to distinguish an effective intervention from less effective or ineffective interventions.[89 90] In addition, surveys have demonstrated that a potential barrier to trial participation is the possibility of being allocated a placebo-only or active control intervention that is perceived to be less desirable than the study intervention.[68 69 91 92] Evidence also suggests that enrolled participants perceive the effect of a given intervention differently depending on whether the control group consists of an active comparator or only placebo.[93-96]

Finally, studies suggest that some "active" comparators in head-to-head randomised trials are presumed by trial investigators to be effective despite having never previously been shown to be superior to placebo.[74 97] In a systematic review of over 100 head-to-head antibiotic trials for mild to moderate chronic obstructive pulmonary disease,[74] cumulative meta-analysis of preceding placebo controlled trials did not show a significant effect of antibiotics over placebo. Such studies again highlight the importance of providing a thorough background and rationale for a trial and the choice of comparators—including data from an up-to-date systematic review—to enable potential participants, physicians, REC/IRBs, and funders to discern the merit of the trial.

Objectives

Item 7: Specific objectives or hypotheses

> **Example**
>
> **"1.1 Research hypothesis**
>
> Apixaban is noninferior to warfarin for prevention of stroke (hemorrhagic, ischemic or of unspecified type) or systemic embolism in subjects with atrial fibrillation (AF) and additional risk factor(s) for stroke.
>
> . . .
>
> 2 STUDY OBJECTIVES
>
> **2.1 Primary objective**
>
> To determine if apixaban is noninferior to warfarin (INR [international normalized ratio] target range 2.0-3.0) in the combined endpoint of stroke (hemorrhagic, ischemic or of unspecified type) and systemic embolism, in subjects with AF and at least one additional risk factor for stroke.
>
> **2.2 Secondary objectives**
>
> 2.2.1 Key secondary objectives
>
> The key secondary objectives are to determine, in subjects with AF and at least one additional risk factor for stroke, if apixaban is superior to warfarin (INR target range 2.0 - 3.0) for,
>
> - the combined endpoint of stroke (hemorrhagic, ischemic or of unspecified type) and systemic embolism
> - major bleeding [International Society of Thrombosis and Hemostasis]
> - all-cause death
>
> 2.2.2 Other secondary objectives
>
> - To compare, in subjects with AF and at least one additional risk factor for stroke, apixaban and warfarin with respect to:
>   The composite endpoint of stroke (ischemic, hemorrhagic, or of unspecified type), systemic embolism and major bleeding, in warfarin naive subjects
>
> . . .
>
> - To assess the safety of apixaban in subjects with AF and at least one additional risk factor for stroke."[98]

Explanation

The study objectives reflect the scientific questions to be answered by the trial, and define its purpose and scope. They are closely tied to the trial design (Item 8) and analysis methods (Item 20). For example, the sample size calculation and statistical analyses for superiority trials will differ from those investigating non-inferiority.

The objectives are generally phrased using neutral wording (eg, "to compare the effect of treatment A versus treatment B on outcome X") rather than in terms of a particular direction of effect.[99] A hypothesis states the predicted effect of the interventions on the trial outcomes. For multiarm trials, the objectives should clarify the way in which all the treatment groups will be compared (eg, A versus B; A versus C).

Trial design

Item 8: Description of trial design including type of trial (eg, parallel group, crossover, factorial, single group), allocation ratio, and framework (eg, superiority, equivalence, non-inferiority, exploratory)

Example

"The PROUD trial is designed as a randomised, controlled, observer, surgeon and patient blinded multicenter superiority trial with two parallel groups and a primary endpoint of wound infection during 30 days after surgery . . . randomization will be performed as block randomization with a 1:1 allocation."[100]

Explanation

The most common design for published randomised trials is the parallel group, two arm, superiority trial with 1:1 allocation ratio.[101] Other trial types include crossover, cluster, factorial, split body, and n of 1 randomised trials, as well as single group trials and non-randomised comparative trials.

For trials with more than one study group, the allocation ratio reflects the intended relative number of participants in each group (eg, 1:1 or 2:1). Unequal allocation ratios are used for a variety of reasons, including potential cost savings, allowance for learning curves, and ethical considerations when the balance of existing evidence appears to be in favour of one intervention over the other.[102] Evidence also suggests a preference of some participants for enrolling in trials with an allocation ratio that favours allocation to an active treatment.[92]

The framework of a trial refers to its overall objective to test the superiority, non-inferiority, or equivalence of one intervention with another, or in the case of exploratory pilot trials, to gather preliminary information on the intervention (eg, harms, pharmacokinetics) and the feasibility of conducting a full-scale trial.

It is important to specify and explain the choice of study design because of its close relation to the trial objectives (Item 7) and its influence on the study methods, conduct, costs,[103] results,[104-106] and interpretation. For example, factorial and non-inferiority trials can involve more complex methods, analyses, and interpretations than parallel group superiority trials.[107 108] In addition, the interpretation of trial results in published reports is not always consistent with the prespecified trial framework,[6 109 110] especially among reports claiming post hoc equivalence based on a failure to demonstrate superiority rather than a specific test of equivalence.[109]

There is increasing interest in adaptive designs for clinical trials, defined as the use of accumulating data to decide how to modify aspects of a study as it continues, without undermining the validity and integrity of the trial.[111 112] Examples of potential adaptations include stopping the trial early, modifying the allocation ratio, re-estimating the sample size, and changing the eligibility criteria. The most valid adaptive designs are those in which the opportunity to make adaptations is based on prespecified decision rules that are fully documented in the protocol (Item 21b).

Section 3a: Methods—participants, interventions, and outcomes

Study setting

Item 9: Description of study settings (eg, community clinic, academic hospital) and list of countries where data will be collected. Reference to where list of study sites can be obtained

Example

"Selection of countries

. . . To detect an intervention-related difference in HIV incidences with the desired power, the baseline incidences at the sites must be sufficiently high. We chose the participating sites so that the average baseline annual incidence across all communities in the study is likely to reach at least 3%. The various sites in sub-Saharan Africa met this criterion, but we also wanted sites in Asia to extend the generalizability of the intervention. The only location in Asia with sufficient incidence at the community level is in ethnic minority communities in Northern Thailand, where HIV incidence is currently in excess of 7%;[reference] thus they were invited to participate as well. Our final selection of sites combines rural (Tanzania, Zimbabwe, Thailand, and KwaZulu-Natal) and an urban (Soweto) location. The cultural circumstances between the sub-Saharan African sites vary widely . . .

Definition of community

Each of the three southern African sites (Harare, Zimbabwe; and Soweto and Vulindlela, South Africa) selected eight communities, the East African (Tanzanian) site selected 10 communities, and Thailand selected 14 communities . . . They are of a population size of approximately 10,000 . . . which fosters social familiarity and connectedness, and they are geographically distinct. Communities are defined primarily geographically for operational purposes for the study, taking into account these dimensions of social communality. The communities chosen within each country and site are selected to be sufficiently distant from each other so that there would be little cross-contamination or little possibility that individuals from a control community would benefit from the activities in the intervention community."[113]

Explanation

A description of the environment in which a trial will be conducted provides important context in terms of the applicability of the study results; the existence and type of applicable local regulation and ethics oversight; and the type of healthcare and research infrastructure available. These considerations can vary substantially within and between countries.

At a minimum, the countries, type of setting (eg, urban versus rural), and the likely number of study sites should be reported in the protocol. These factors have been associated with recruitment success and degree of attrition for some trials,[68 91 92 114-117] but not for others.[118 119] Trial location has also been associated with trial outcome,[120] aspects of trial quality (eg, authenticity of randomisation[121]), and generalisability.[122]

Eligibility criteria

Item 10: Inclusion and exclusion criteria for participants. If applicable, eligibility criteria for study centres and individuals who will perform the interventions (eg, surgeons, psychotherapists)

Explanation

Eligibility criteria for potential trial participants define the study population. They can relate to demographic information; type or severity of the health condition; comorbidities; previous or current treatment; diagnostic procedures; pregnancy; or other relevant considerations.[125] In trials of operator-dependent interventions such as surgery and psychotherapy, it is usually important to promote consistency of intervention delivery by also defining the eligibility criteria for care providers and centres where the intervention will be administered.[126]

Clear delineation of eligibility criteria serves several purposes. It enables study personnel to apply these criteria consistently throughout the trial.[127] The choice of eligibility criteria can affect recruitment and attri-

Examples

"Patients (or a representative) must provide written, informed consent before any study procedures occur (see Appendix 1 for sample Informed Consent Form) . . .

5.1. Inclusion Criteria

Patients eligible for the trial must comply with all of the following at randomization:

1. Age ≥16 years
2. Current admission under the care of the heart-failure service at the site

. . .

5.2. Exclusion Criteria

1. Acute decompensation thought by the attending heart-failure physician to require or be likely to require PAC [pulmonary-artery catheter] during the next 24 hours. Such patients should be entered into the PAC Registry (see below).
2. Inability to undergo PAC placement within the next 12 hours

. . .

Patients enrolled in other investigational drug studies are potential candidates for ESCAPE. As the ESCAPE protocol does not involve any investigational agents or techniques, patients would be eligible for dual randomization if they are on stable doses of the investigational drugs. . . .

13. Study Network, Training, and Responsibilities

. . . To qualify, physicians responsible for PAC [pulmonary-artery catheter] placements will be required to show proof of insertion of ≥50 PACs in the previous year with a complication rate of <5%. Further, clinicians will need to show competence in the following areas to participate in the study: 1) insertion techniques and cardiovascular anatomy; 2) oxygen dynamics; . . . and 7) common PAC complications. [reference] . . . we will assume basic competence in these areas after satisfactory completion of the PACEP [PAC educational programme] module."[123]

"Trial centre requirements

A number of guidelines have stated thrombolysis should only be considered if the patient is admitted to a specialist centre with appropriate experience and expertise.[reference] Hospitals participating in IST-3 [third International Stroke Trial] should have an organized acute stroke service. The components of effective stroke unit care have been identified . . . In brief, the facilities (details of these requirements are specified in the separate operations manual) should include:

- Written protocol for the acute assessment of patients with suspected acute stroke to include interventions to reduce time from onset to treatment.
- Immediate access to CT [computed tomographic] or MR [magnetic resonance] brain scanning (preferably 24 hours a day).

A treatment area where thrombolysis may be administered and the patient monitored according to trial protocol, preferably an acute stroke unit."[124]

tion,[67 114 115 117 118 128-130] as well as outcome event rates.[39 131] In addition, the criteria convey key information related to external validity (generalisability or applicability).[132] The importance of transparent documentation is highlighted by evidence that the eligibility criteria listed in publications are often different from those specified in the protocol.[125 133 134]

Certain eligibility criteria warrant explicit justification in the protocol, particularly when they limit the trial sample to a narrow subset of the population.[132 135 136] The appropriateness of restrictive participant selection depends on the trial objectives.[137] When trial participants differ substantially from the overall population to whom the intervention will be applied, the trial results may not reflect the impact in real world practice settings.[134 138-144]

Interventions

Item 11a: Interventions for each group with sufficient detail to allow replication, including how and when they will be administered

Explanation

Studies of trials and systematic reviews have shown that important elements of the interventions are not described in half of the publications.[146 147] If such elements are also missing from the protocol, or if the protocol simply refers to other documents that are not freely accessible, then it can be impossible for healthcare providers, systematic reviewers, policymakers, and others to fully understand, implement, or evaluate the trial intervention.[148] This principle applies to all types of interventions, but is particularly true for complex interventions (eg, health service delivery; psychotherapy), which consist of interconnected components that can vary between healthcare providers and settings.

For drugs, biological agents, or placebos, the protocol description should include the generic name, manufacturer, constituent components, route of administration, and dosing schedule (including titration and run-in periods, if applicable).[149 150] The description of non-drug interventions—such as devices, procedures, policies, models of care, or counselling—is generally more complex and warrants additional details about the setting (Item 9) and individuals administering the interventions. For example, the level of pre-trial expertise (Item 10) and specific training of individuals administering these complex interventions are often relevant to describe (eg, for surgeons, psychologists, physiotherapists). When intervention delivery is subject to variation, it is important to state whether the same individuals will deliver the trial interventions in all study groups, or whether different individuals will manage each study group—in which case it can be difficult to separate the effect of the intervention from that of the individual delivering it. Interventions that consist of "usual care" or "standard of care" require further elaboration in the protocol, as this care can vary substantially across centres and patients, as well as over the duration of the trial.

Example

"Eligible patients will be randomised in equal proportions between IL-1ra [interleukin-1 receptor antagonist] and placebo, receiving either a once daily, subcutaneous (s.c.) injection of IL-1ra (dose 100 mg per 24 h) for 14 days, or a daily s.c. injection of placebo for 14 days . . .

The study drug and placebo will be provided by Amgen Inc in its commercially available recombinant form . . . The study drug and placebo will be relabelled by Amgen, in collaboration with CTEU [Clinical Trials and Evaluation Unit] according to MHRA [Medicines and Healthcare Products Regulatory Agency] guidelines.

The first dose of IL-1ra will be given within 24 h +2 h of the positive Troponin. Injections will be given at a standardised time (24 ± 2 h after the previous dose), immediately after blood sampling. IL-1ra or placebo will [be] administered to the patient by the research nurse while the patient is in hospital. During the hospital stay, the patient will be taught to self-administer the injection by the research nurse and on discharge will continue at home. This has proven possible in other ACS [acute coronary syndrome] trials that required self injection of subcutaneous heparin [reference]. Full written guidance on self injection will also be provided to patients. If self injection is found not to be possible in an individual patient for unexpected reasons, an alternative method will be sought (eg district nurse, or attending the hospital) to try and maintain full compliance with scheduled study drug regimen after discharge. Patients will also be asked to complete a daily injection diary. All personnel will be blinded to the identity of the syringe contents."[145]

Interventions—modifications

Item 11b: Criteria for discontinuing or modifying allocated interventions for a given trial participant (eg, drug dose change in response to harms, participant request, or improving/worsening disease)

Explanation

For a given trial participant, the assigned study intervention may need to be modified or discontinued by

chaa006386.indd 10 31/01/2013 10:33:37

Example

"Gastro-Intestinal Upset

The tablets may be taken in two equally divided doses, if necessary, to improve gastro-intestinal tolerance. Should it be necessary the daily dose may be reduced by one tablet at a time to improve gastro-intestinal tolerance.

Renal Function Impairment

Since sodium clodronate is excreted unchanged by the kidney its use is contra-indicated in patients with moderate to severe renal impairment (serum creatinine greater than 2 times upper limit of normal range of the centre). If renal function deteriorates to this extent the trial medication should be withdrawn from the patient. This should be reported as an adverse event. In patients with normal renal function or mild renal impairment (serum creatinine less than 2 times upper limit of normal range of the centre) serum creatinine should be monitored during therapy.

Allergic Reactions

Allergic skin reactions have been observed in rare cases. If this is suspected withdraw the trial medication from the patient. This should be reported as an adverse event.

Biochemical Disturbances

Asymptomatic hypocalcaemia has been noted rarely. Temporary suspension of the trial medication until the serum calcium returns into the normal range is recommended. The trial medication can be then restarted at half the previous dose. If the situation returns withdraw the trial medication from the patient. This should be reported as an adverse event . . ."[151]

trial investigators for various reasons, including harms, improved health status, lack of efficacy, and withdrawal of participant consent. Comparability across study groups can be improved, and subjectivity in care decisions reduced, by defining standard criteria for intervention modifications and discontinuations in the protocol. Regardless of any decision to modify or discontinue their assigned intervention, study participants should be retained in the trial whenever possible to enable follow-up data collection and prevent missing data (Item 18b).[152]

Interventions—adherence

Item 11c: Strategies to improve adherence to intervention protocols, and any procedures for monitoring adherence (eg, drug tablet return; laboratory tests)

Example

"Adherence reminder sessions

Face-to-face adherence reminder sessions will take place at the initial product dispensing and each study visit thereafter. This session will include:

- The importance of following study guidelines for adherence to once daily study product
- Instructions about taking study pills including dose timing, storage, and importance of taking pills whole, and what to do in the event of a missed dose.
- Instructions about the purpose, use, and care of the MEMS® cap [medication event monitoring system] and bottle
- Notification that there will be a pill count at every study visit
- Reinforcement that study pills may be TDF [tenofovir disproxil fumarate] or placebo
- Importance of calling the clinic if experiencing problems possibly related to study product such as symptoms, lost pills or MEMS® cap.

Subsequent sessions will occur at the follow-up visits. Participants will be asked about any problems they are having taking their study pills or using the MEMS® cap. There will be brief discussion of reasons for missed doses and simple strategies for enhancing adherence, eg, linking pill taking to meals or other daily activities. Participants will have an opportunity to ask questions and key messages from the initial session will be reviewed as needed . . .

Adherence assessments

To enhance validity of data, multiple methods will be used to assess medication adherence including pill count; an electronic medication event monitoring system (MEMS® cap) [reference]; and ACASI [audio-computer administered interview] questionnaire items including a one month visual analogue scale,[reference] reasons for non-compliance, and use of the MEMS® cap. Participants will return the unused tablets and bottle at each follow-up visit. Unused tablets will be counted and recorded on the appropriate CRF [case report form]. Electronic data collected in the MEMS® cap will be downloaded into a designated, secure study computer."[153]

Explanation

Adherence to intervention protocols refers to the degree to which the behaviour of trial participants corresponds to the intervention assigned to them.[154] Distinct but related concepts include trial retention (Item 18b) and adherence to the follow-up protocol of procedures and assessments (Item 13).

On average, adherence to intervention protocols is higher in clinical trials than in non-research settings.[155] Although there is no consensus on the acceptable minimum adherence level in clinical trials, low adherence can have a substantial effect on statistical power and interpretation of trial results.[156-158] Since fewer participants are receiving the full intervention as intended, non-adherence can reduce the contrast between study groups—leading to decreased study power and increased costs associated with recruiting larger sample sizes for evaluating superiority, or leading to potentially inappropriate conclusions of non-inferiority or equivalence. There is also the possibility of underestimating any efficacy and harms of the study intervention.

Furthermore, if adherence is a marker for general healthy behaviour associated with better prognosis, then different rates of non-adherence between study groups can lead to a biased estimate of an intervention's effect. In support of this "healthy adherer" effect, non-adherers to placebo in clinical studies have been found to have poorer clinical outcomes than adherers.[159]

To help avoid these potential detrimental effects of non-adherence, many trials implement procedures and strategies for monitoring and improving adherence,[67 156-158] and any such plans should be described in the protocol.[160] Among applicable drug trials published in 1997-99, 47% reported monitoring the level of adherence.[161] Although each of the many types of monitoring methods has its limitations,[157 158] adherence data can help to inform the statistical analysis (Item 20c), trial interpretation, and choice of appropriate adherence strategies to implement in the trial as it progresses or in future trials and clinical practice.

A variety of adherence strategies exist,[156-158] and their use can be tailored to the specific type of trial design, intervention, and participant population. It may be desirable to select strategies that can be easily implemented in clinical practice, so that the level of adherence in the real world setting is comparable to that observed in the trial.[158]

Interventions—concomitant care

Item 11d: Relevant concomitant care and interventions that are permitted or prohibited during the trial

Explanation

In a controlled trial, a key goal is to have comparable study groups that differ only by the intervention being evaluated, so that any difference in outcomes can be attributed to effects of the study intervention. Cointervention bias can arise when the study groups receive different concomitant care or interventions (in addition to the assigned trial interventions) that may affect trial outcomes.[162] To promote comparability of study groups, the protocol should list the relevant concomitant care and interventions that are allowed (including rescue interventions), as well as any that are prohibited.

chaa006386.indd 11 31/01/2013 10:33:37

Example

"2. Rescue Medication

For weeks 0-3, topical mometasone furoate 0.1% cream or ointment (30 g/week) will be permitted with participants preferably using ointment. Participants will be instructed to apply the topical mometasone furoate to blisters/lesions as required (not to areas of unaffected skin). If the participant is allergic to mometasone furoate or the hospital pharmacy does not stock it, then an alternative topical steroid may be prescribed but this must be in the potent class. In addition, participants will be advised that they can apply a light moisturiser to blisters/lesions at any time during the study.

For weeks 3-6, use of mometasone furoate (or other topical corticosteroids) is strongly discouraged to prevent potential systemic effects. Accidental use of mometasone furoate or other potent topical steroid during this period will be classified as a protocol deviation.

After week 6, potent topical corticosteroids (up to 30 g/week) may be used to treat symptoms and localised disease if they would have normally been used as part of normal clinical care by the physician in charge of that patient. This must be recorded on the trial treatment log.

However, those patients who are on a dose reducing regime for oral steroids, 30 g/week of a "potent" topical steroid will be allowed.

3. Prohibited Concomitant Medications

The administration of live virus vaccines is not permitted for all participants during weeks 0-6 as the investigator is blinded to treatment allocation, and must therefore warn all participants to refrain for [sic] having a live virus vaccine. However, after week 6, once the investigator knows which medication the participant is on, only those taking prednisolone will not be allowed live virus vaccines. Participants should continue to take medications for other conditions as normal. However, if it is anticipated that the participant will need a live virus vaccine during the intervention phase, they will be ineligible for entry into the study . . ."[50]

Outcomes

Item 12: Primary, secondary, and other outcomes, including the specific measurement variable (eg, systolic blood pressure), analysis metric (eg, change from baseline, final value, time to event), method of aggregation (eg, median, proportion), and time point for each outcome. Explanation of the clinical relevance of chosen efficacy and harm outcomes is strongly recommended

Example

"1. Primary Outcome Measures

- Difference between the two treatment arms in the proportion of participants classed as treatment success at 6 weeks. Treatment success is defined as 3 or less significant blisters present on examination at 6 weeks. Significant blisters are defined as intact blisters containing fluid which are at least 5 mm in diameter. However, if the participant has popped a blister, or the blister is at a site that makes it susceptible to bursting such as the sole of the foot, it can be considered part of the blister count, providing there is a flexible (but not dry) roof present over a moist base. Mucosal blisters will be excluded from the count.

A survey of the UK DCTN [Dermatology Clinical Trials Network] membership showed that a point estimate of 25% inferiority in effectiveness would be acceptable assuming a gain in the safety profile of at least 10%.

- This measure of success was selected as it was considered to be more clinically relevant than a continuous measure of blister count. It would be less clinically relevant to perform an absolute blister count and report a percentage reduction. Instead, to state that treatment is considered a success if remission is achieved (ie the presence of three or less blisters on physical examination at 6 weeks) more closely reflects clinical practice. In addition, it is far less burdensome on investigators than including a full blister count, which would mean counting in the region of 50-60 blisters in many cases. This outcome measure will be performed as a single blind assessment.
- Difference between the two treatment arms in the proportion of participants reporting grade 3, 4 and 5 (mortality) adverse events which are possibly, probably or definitely related to BP [bullous pemphigoid] medication in the 52 weeks following randomisation. A modified version of The Common Terminology Criteria for Adverse Events (CTCAE v3.0) will be used to grade adverse events. At each study visit, participants will be questioned about adverse events they have experienced since the last study visit (using a standard list of known side effects of the two study drugs).

2. Secondary Outcome Measures

For the secondary and tertiary endpoints a participant will be classed as a treatment success if they have 3 or less significant blisters present on examination and have not had their treatment modified (changed or dose increased) on account of a poor response.

- Difference in the proportion of participants who are classed as a treatment success at 6 weeks.
- Difference in the proportion of participants in each treatment arm who are classed as treatment success at 6 weeks and are alive at 52 weeks. This measure will provide a good overall comparison of the two treatment arms."[50]

Explanation

The trial outcomes are fundamental to study design and interpretation of results. For a given intervention, an outcome can generally reflect efficacy (beneficial effect) or harm (adverse effect). The outcomes of main interest are designated as primary outcomes, which usually appear in the objectives (Item 7) and sample size calculation (Item 14). The remaining outcomes constitute secondary or other outcomes.

For each outcome, the trial protocol should define four components: the specific measurement variable, which corresponds to the data collected directly from trial participants (eg, Beck Depression Inventory score, all cause mortality); the participant-level analysis metric, which corresponds to the format of the outcome data that will be used from each trial participant for analysis (eg, change from baseline, final value, time to event); the method of aggregation, which refers to the summary measure format for each study group (eg, mean, proportion with score > 2); and the specific measurement time point of interest for analysis.[163]

It is also important to explain the rationale for the choice of trial outcomes. An ideal outcome is valid, reproducible, relevant to the target population (eg, patients), and responsive to changes in the health condition being studied.[67] The use of a continuous versus dichotomous method of aggregation can affect study power and estimates of treatment effect,[164 165] and subjective outcomes are more prone to bias from inadequate blinding (ascertainment bias) and allocation concealment (selection bias) than objective outcomes.[166 167] Although composite outcomes increase event rates and statistical power, their relevance and interpretation can be unclear if the individual component outcomes vary greatly in event rates, importance to patients, or amount of missing data.[168-171]

The number of primary outcomes should be as small as possible. Although up to 38% of trials define multiple primary outcomes,[4 35 163] this practice can introduce problems with multiplicity, selective reporting, and interpretation when there are inconsistent results across outcomes. Problems also arise when trial protocols do not designate any primary outcomes, as seen in half (28/59) of protocols for a sample of trials published from 2002-2008,[12] and in 25% of randomised trial protocols that received ethics approval in Denmark in 1994-95.[4] Furthermore, major discrepancies in the primary outcomes designated in protocols/registries/regulatory submissions versus final trial publications are common; favour the reporting of statistically significant primary outcomes over non-significant ones; and are often not acknowledged in final publications.[172-176] Such bias can only be identified and deterred if trial outcomes are clearly defined beforehand in the protocol and if protocol information is made public.[177]

Where possible, the development and adoption of a common set of key trial outcomes within a specialty can help to deter selective reporting of outcomes and to facilitate comparisons and pooling of results across trials in a meta-analysis.[178-180] The COMET (Core Outcome Measures in Effectiveness Trials) Initiative aims to facilitate the development and application of such standardised sets of core outcomes for clinical trials of specific conditions (www.comet-initiative.org). Trial investigators are encouraged to ascertain whether there is a core outcome set relevant to their trial and, if so, to include those outcomes in their trial. Existence of a common set of outcomes does not preclude inclusion of additional relevant outcomes for a given trial.

chaa006386.indd 12 31/01/2013 10:33:37

Participant timeline

Item 13: Time schedule of enrolment, interventions (including any run-ins and washouts), assessments, and visits for participants. A schematic diagram is highly recommended (see fig 1)

Examples

"The main outcomes of interest are the drug and sex-related HIV and HCV [hepatitis C virus] risk behaviors . . . Clients will be assessed using the full battery of instruments from the Common Assessment Battery (CAB), along with the Self-Efficacy and Stages of Change questionnaires and a Urine Drug Screen after consenting . . . questionnaires will take place for all participants 14-30 days after randomization during which they will be given the Stages of Change and Self-Efficacy questionnaires, the Timeline Follow-Back, and a UA [urine analysis]. Follow-up interviews, using the full battery (CAB and questionnaires), will be collected at 2 months (56 days), 4 months (112 days) and 6 months (168 days) after the randomization date. A 14 day window, defined as 7 days before and 7 days after the due date, will be available to complete the 2 and 4 month follow-up interviews and a 28 day window, defined as 7 days before and 21 days after the due date, will be available to complete the 6 month follow up interview . . .

7.1.1 Common Assessment Battery (CAB)

A Demographic Questionnaire . . .

The Composite International Diagnostic Interview Version 2.1 . . .

The Addiction Severity Index-Lite (ASI-Lite) . . .

The Risk Behavior Survey (RBS), . . .

7.1.2 Additional Interviews/Questionnaires

To assess drug use, urinalysis for morphine, cocaine, amphetamine, and methamphetamine will be performed at the 2-Week Interim Visit, and the 2-, 4-, and 6-month Follow-up visits . . .

Stage of change for quitting drug use will be measured using a modification of the Motivation Scales [table 3] . . ."[181]

"The trial consists of a 12-week intervention treatment phase with a 40-week follow-up phase. The total trial period will be 12 months. As shown . . . measurements will be undertaken at four time-points in each group: at baseline, directly after completing the 12-week internet program, and at six and 12-month follow-up [see fig 2]."[182]

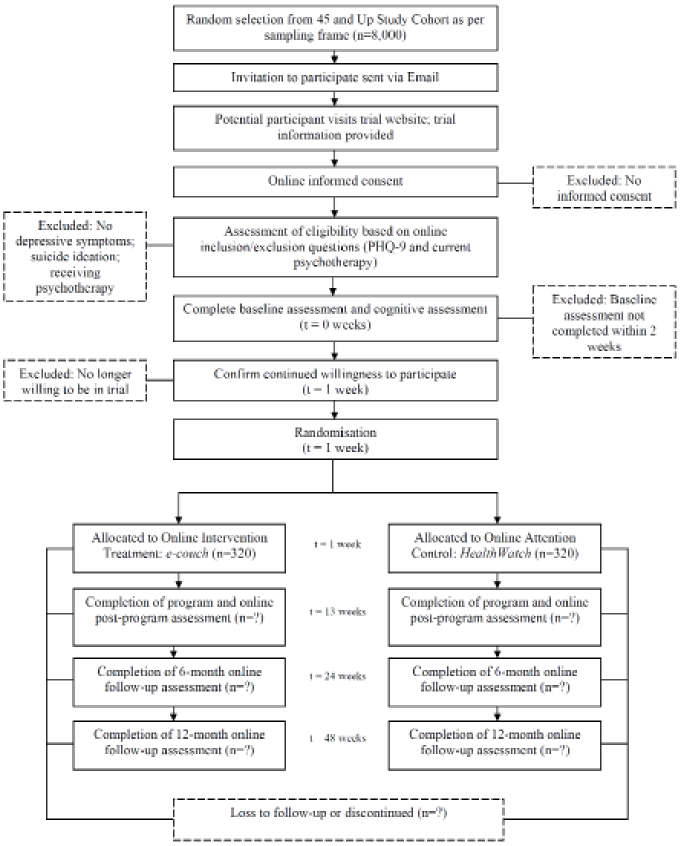

Fig 2: Flow of participants[182]

Explanation

A clear and concise timeline of the study visits, enrolment process, interventions, and assessments performed on participants can help to guide trial conduct and enable external review of participant burden and feasibility. These factors can also affect the decision of potential investigators and participants to join the trial (Item 15).[91]

A schematic diagram is highly recommended to efficiently present the overall schedule and time commitment for trial participants in each study group. Though various presentation formats exist, key information to convey includes the timing of each visit, starting from initial eligibility screening through to study close-out; time periods during which trial interventions will be administered; and the procedures and assessments performed at each visit (with reference to specific data collection forms, if relevant) (fig 1).

Sample size

Item 14: Estimated number of participants needed to achieve study objectives and how it was determined, including clinical and statistical assumptions supporting any sample size calculations

Explanation

The planned number of trial participants is a key aspect of study design, budgeting, and feasibility that is usually determined using a formal sample size calculation. If the planned sample size is not derived statistically, then this should be explicitly stated along with a rationale for the intended sample size (eg, exploratory nature of pilot studies; pragmatic considerations for trials in rare diseases).[17 184]

For trials that involve a formal sample size calculation, the guiding principle is that the planned sample size should be large enough to have a high probability (power) of detecting a true effect of a given magnitude, should it exist. Sample size calculations are generally based on one primary outcome; however, it may also be worthwhile to plan for adequate study power or report the power that will be available (given the proposed sample size) for other important outcomes or analyses because trials are often underpowered to detect harms or subgroup effects.[185 186]

Among randomised trial protocols that describe a sample size calculation, 4-40% do not state all components of the calculation.[6 11] The protocol should generally include the following: the outcome (Item 12); the values assumed for the outcome in each study group (eg, proportion with event, or mean and standard deviation) (table 4); the statistical test (Item 20a); alpha (type 1 error) level; power; and the calculated sample size per group—both assuming no loss of data and, if relevant, after any inflation for anticipated missing data (Item 20c). Trial investigators are also encouraged

Table 4 | Outcome values to report in sample size calculation

	Type of summary outcome		
Element	Binary	Continuous	Time to event
Assumed result for each study group	Proportion (%) with event	Mean and standard deviation	Proportion (%) with event at a given time point
Effect measure	Relative risk, odds ratio	Difference in means	Hazard ratio

Note: Although the sample size calculation uses the expected outcome value for each group, the corresponding contrast between groups (estimated effect) should also be reported.

chaa006386.indd 13 31/01/2013 10:33:37

Table 3 | HIV/HCV risk reduction protocol schedule of forms and procedures (adapted from original table[181])

Activity/ assessment	CRF (Yes/No)	Staff member	Approximate time to complete (min)	-1 Prestudy screening/ consent	0 Prestudy baseline/ randomisation	T1 Study visit 1	T2 Study/interim visit 2 and/or 2 week interim	F1 Follow-up 2 months	F2 Follow-up 4-months	F3 Follow-up 6-months
Prescreening consent	No	Study coordinator	5	X						
Screening log	No	Study coordinator	5	X						
Consent form/quiz	No	Study coordinator	45	X						
Inclusion/exclusion form	Yes	Study coordinator	N/A	X						
Urine screen	Yes	Study coordinator	10		X		X	X	X	X
Locator form	No	Interviewer	10		X		Update X	Update X	Update X	
Demographics questionnaire	Yes	Interviewer	10		X					
Addiction severity index (ASI) lite	Yes	Interviewer	45		X			X	X	X
Composite international diagnostic interview)	Yes	Interviewer	45		X					
HIV risk behaviour survey	Yes	Interviewer	15		X			X	X	X
Timeline follow back	Yes	Interviewer	5				X	X	X	X
Self efficacy	Yes	Interviewer	5		X		X	X	X	X
Stage of change	Yes	Interviewer	5		X		X	X	X	X
Randomisation	Yes	Study coordinator	15		X					
Voluntary blood sample Counselling and education intervention (treatment group)	Yes	Study phlebotomist	15			X				X
All groups, optional blood sample at study close	Yes	Study phlebotomist	15							X
Termination form	Yes	Study coordinator	N/A							X
Serious adverse event form	Yes	Study coordinator	N/A	As needed throughout protocol						
Progress notes	No	All team members	N/A	X	X	X	X	X	X	X
Communication log	No	All team members	N/A	Every phone or in-person contact outside of a regular visit						

Examples

"The sample size was calculated on the basis of the primary hypothesis. In the exploratory study,[reference] those referred to PEPS [psychoeducation with problem solving] had a greater improvement in social functioning at 6 month follow-up equivalent to 1.05 points on the SFQ [Social Functioning Questionnaire]. However, a number of people received PEPS who were not included in the trial (eg, the wait-list control) and, for this larger sample (N=93), the mean pre-post- treatment difference was 1.79 (pre-treatment mean=13.85, SD=4.21; post-treatment mean=12.06, SD=4.21). (Note: a lower SFQ score is more desirable). This difference of almost 2 points accords with other evidence that this is a clinically significant and important difference.[reference] A reduction of 2 points or more on the SFQ at 1 year follow-up in an RCT of cognitive behaviour therapy in health anxiety was associated with a halving of secondary care appointments (1.24.vs 0.65), a clinically significant reduction in the Hospital Anxiety and Depression Scale (HADS[reference]) Anxiety score of 2.5 (9.9 vs 7.45) and a reduction in health anxiety (the main outcome) of 5.6 points (17.8 vs 12.2) (11 is a normal population score and 18 is pathological).[reference] These findings suggest that improvements in social functioning may accrue over 1 year, hence we expect to find a greater magnitude of response at the 72 week follow-up than we did in the exploratory trial. Therefore, we have powered this trial to be able to detect a difference in SFQ score of 2 points. SFQ standard deviations vary between treatment, control, and the wait-list samples, ranging from 3.78 to 4.53. We have based our sample size estimate on the most conservative (ie, largest) SD [standard deviation]. To detect a mean difference in SFQ score of 2 point (SD = 4.53) at 72 weeks with a two-sided significance level of 1% and power of 80% with equal allocation to two arms would require 120 patients in each arm of the trial. To allow for 30% drop out, 170 will be recruited per arm, ie, 340 in total."[183]

"Superficial and deep incisional surgical site infection rates for patients in the PDS II® [polydioxanone suture] group are estimated to occur at a rate of 0.12.[reference] The trials by [reference] have shown a reduction of SSI [surgical site infections] of more than 50% (from 10.8% to 4.9% and from 9.2% to 4.3% respectively). Therefore, we estimate a rate of 0.06 for PDS Plus® [triclosan-coated continuous polydioxanone suture].

For a fixed sample size design, the sample size required to achieve a power of $1-\beta=0.80$ for the one-sided chi-square test at level $\alpha=0.025$ under these assumptions amounts to $2\times356=712$ (nQuery Advisor®, version 7.0). It can be expected that including covariates of prognostic importance in the logistic regression model as defined for the confirmatory analysis will increase the power as compared to the chi-square test. As the individual results for the primary endpoint are available within 30 days after surgery, the drop-out rate is expected to be small. Nevertheless, a potential dilution of the treatment effect due to drop-outs is taken into account (eg no photographs available, loss to follow up); it is assumed that this can be compensated by additional 5% of patients to be randomized, and therefore the total sample size required for a fixed sample size design amounts to n=712+38=750 patients.

. . .

An adaptive interim analysis [reference] will be performed after availability of the results for the primary endpoint for a total of 375 randomized patients (ie, 50% of the number of patients required in a fixed sample size design). The following type I error rates and decision boundaries for the interim and the final analysis are specified:

- Overall one-sided type I error rate: 0.025
- Boundary for the one-sided p-value of the first stage for accepting the null-hypothesis within the interim analysis: $\alpha_0=0.5$
- One-sided local type I error rate for testing the null-hypothesis within the interim analysis: $\alpha_1=0.0102$
- Boundary for the product of the one-sided p-values of both stages for the rejection of the null-hypothesis in the final analysis: $c\alpha=0.0038$

If the trial will be continued with a second stage after the interim analysis (this is possible if for the one-sided p-value p_1 of the interim analysis $p_1 \in]0.0102,0.5[$ [ie $0.5 \geq P_1 \geq 0.0102$] holds true, the results of the interim analysis can be taken into account for a recalculation of the required sample size. If the sample size recalculation leads to the conclusion that more than 1200 patients are required, the study is stopped, because the related treatment group difference is judged to be of minor clinical importance.

. . .

The actually achieved sample size is then not fixed but random, and a variety of scenarios can be considered. If the sample size is calculated under the same assumptions with respect to the SSI rates for the two groups, applying the same the overall significance level of $\alpha=0.025$ (one-sided) but employing additionally the defined stopping boundaries and recalculating the sample size for the second stage at a conditional power of 80% on the basis of the SSI rates observed in the interim analysis results in an average total sample size of n=766 patients; the overall power of the study is then 90% (ADDPLAN®, version 5.0)."[100]

to provide a rationale or reference for the outcome values assumed for each study group.[187] The values of certain pre-specified variables tend to be inappropriately inflated (eg, clinically important treatment effect size)[188 189] or underestimated (eg, standard deviation for continuous outcomes),[190] leading to trials having less power in the end than what was originally calculated. Finally, when uncertainty of a sample size estimate is acknowledged, methods exist for re-estimating sample size.[191] The intended use of such an adaptive design approach should be stated in the protocol.

For designs and frameworks other than parallel group superiority trials, additional elements are required in the sample size calculation. For example, an estimate of the standard deviation of within-person changes from baseline should be included for crossover trials[192]; the intracluster correlation coefficient for cluster randomised trials[193]; and the equivalence or non-inferiority margin for equivalence or non-inferiority trials respectively.[108 194] Such elements are often not described in final trial reports,[110 195-198] and it is unclear how often they are specified in the protocol.

Complete description of sample size calculations in the protocol enables an assessment of whether the trial will be adequately powered to detect a clinically important difference.[189 199-206] It also promotes transparency and discourages inappropriate post hoc revision that is intended to support a favourable interpretation of results or portray consistency between planned and achieved sample sizes.[6 207]

Recruitment

Item 15: Strategies for achieving adequate participant enrolment to reach target sample size

Explanation

The main goal of recruitment is to meet the target sample size (Item 14). However, recruitment difficulties are commonly encountered in clinical trials.[209-213] For example, reviews of government funded trials in the US and UK found that two thirds did not reach their recruitment targets.[214 215] Low enrolment will reduce statistical power and can lead to early trial stoppage or to extensions with delayed results and greater costs.

Strategies to promote adequate enrolment are thus important to consider during trial planning. Recruitment strategies can vary depending on the trial topic, context, and site. Different recruitment methods can substantially affect the number and type of trial participants recruited[128 209 216-220] and can incur different costs.[221-223] Design issues such as the number and stringency of eligibility criteria will also directly affect the number of eligible trial participants.

Protocol descriptions of where participants will be recruited (eg, primary care clinic, community), by whom (eg, surgeon), when (eg, time after diagnosis), and how (eg, advertisements, review of health records) can be helpful for assessing the feasibility of achieving the target sample size and the applicability of the trial results in practice. Other relevant information to explicitly provide in the protocol includes expected recruitment rates, duration of the recruitment period, plans to monitor recruitment during the trial, and any financial or non-financial incentives provided to trial investigators or participants for enrolment (Item 4). If strategies differ by site in multicentre trials, these should be detailed to the extent possible.

Example

"Each center will screen subjects to achieve screening percentages of 50% women and 33% minority; screening will continue until the target population is achieved (12 subjects/site). We recognize that, because of exclusion by genotype and genotypic variation among diverse populations,[reference], the enrolled cohort may not reflect the screened population. The enrollment period will extend over 12 months.

Recruitment Strategy

Each clinical center involved in the ACRN [Asthma Clinical Research Network] was chosen based on documentation for patient availability, among other things. It is, however, worthy to note the specific plans of each center.

. . . The Asthma Clinical Research Center at the Brigham & Women's Hospital utilizes three primary resources for identifying and recruiting potential subjects as described below.

1. Research Patient Database

The Asthma Clinical Research Center at the Brigham and Women's Hospital has a database of over 1,500 asthmatics . . .

2. Asthma Patient Lists . . .

3. Advertisements . . .

. . . the Madison ACRN site has utilized some additional approaches to target minority recruitment. We have utilized a marketing expert to coordinate and oversee our overall efforts in recruiting and retaining minorities. . . . As a result of his efforts, we have advertised widely in newspapers and other publications that target ethnic minorities, established contacts with various ethnic community, university, church, and business groups, and conducted community-based asthma programs . . . For example, student groups such as AHANA (a pre-health careers organization focusing on minority concerns) will be contacted. . . . In addition, we will utilize published examples of successful retention strategies such as frequent payment of subject honoraria as study landmarks are achieved and study participant group social events. Study visits will be carefully planned and scheduled to avoid exam-time and university calendar breaks . . .

The Harlem Hospital Center Emergency Department (ED) sees an average of eight adult patients per day for asthma. Through the REACH (Reducing Emergency Asthma Care in Harlem) project, we have . . . successfully recruited and interviewed 380 patients from the ED . . .

Responses to inquiries about participation in research studies are answered by a dedicated phone line that is manned during business hours and answered by voicemail at all other times. A research assistant responds to each inquiry immediately, using a screening instrument . . .

Patients are recruited for clinical trials at the Jefferson Center through two primary mechanisms: (1) local advertising; and (2) identification in the asthma patient registry (database). Local advertising takes advantage of the printed as well as the audio-visual media. Printed media include . . . All advertising in the printed and audio-visual media has prior approval of the Institutional Review Board.

The Jefferson patient registry (database) has been maintained since 1992 and currently contains 3,100 patients . . . It is estimated that 300-400 new asthmatic patients are seen each year, while a smaller number become inactive due to relocation, change of health care provider, etc. Once identified in the database, patients potentially eligible for a specific study are contacted by the nurse coordinator who explains the study and ascertains the patient's interest. If interested, the patient is seen in the clinical research laboratories where more detailed evaluations are made . . .

Each subject will receive financial compensation within FDA [Food and Drug Administration] guidelines for participation in an amount determined by the local center. For subjects who drop out, payments will be pro-rated for the length of time they stayed in the study, but payment will not be made until the study would have been completed had the subject not dropped out."[208]

chaa006386.indd 15 31/01/2013 10:33:38

Section 3b: Methods—assignment of interventions (for controlled trials)

Allocation—sequence generation

Item 16a: Method of generating the allocation sequence (eg, computer-generated random numbers) and list of any factors for stratification. To reduce predictability of a random sequence, details of any planned restriction (eg, blocking) should be provided in a separate document that is unavailable to those who enrol participants or assign interventions

Example

"Participants will be randomly assigned to either control or experimental group with a 1:1 allocation as per a computer generated randomisation schedule stratified by site and the baseline score of the Action Arm Research Test (ARAT; <=21 versus >21) using permuted blocks of random sizes. The block sizes will not be disclosed, to ensure concealment."[224]

Explanation

Participants in a randomised trial should be assigned to study groups using a random (chance) process characterised by unpredictability of assignments. Randomisation decreases selection bias in allocation; helps to facilitate blinding/masking after allocation; and enables the use of probability theory to test whether any difference in outcome between intervention groups reflects chance.[17 225-227] Use of terms such as "randomisation" without further elaboration is not sufficient to describe the allocation process, as these terms have been used inappropriately to describe non-random, deterministic allocation methods such as alternation or allocation by date of birth.[121] In general, these non-random allocation methods introduce selection bias and biased estimates of an intervention's effect size,[17 167 228 229] mainly due to the lack of allocation concealment (Item 16b). If non-random allocation is planned, then the specific method and rationale should be stated. Box 1 outlines the key elements of the random sequence that should be detailed in the protocol. Three quarters of randomised trial protocols approved by a research ethics committee in Denmark (1994-95) or conducted by a US cooperative cancer research group (1968-2006) did not describe the method of sequence generation.[2 11]

Box 1 | Key elements of random sequence to specify in trial protocols

- Method of sequence generation (eg, random number table or computerised random number generator)
- Allocation ratio (Item 8) (eg, whether participants are allocated with equal or unequal probabilities to interventions)
- Type of randomisation (box 2): simple versus restricted; fixed versus adaptive (eg, minimisation); and, where relevant, the reasons for such choices
- If applicable, the factors (eg, recruitment site, sex, disease stage) to be used for stratification (box 2), including categories and relevant cut-off boundaries

Box 2 | Randomisation and minimisation (adapted from CONSORT 2010 Explanation and Elaboration)[17 230 231]

Simple randomisation

Randomisation based solely on a single, constant allocation ratio is known as simple randomisation. Simple randomisation with a 1:1 allocation ratio is analogous to a coin toss, although tossing a coin is not recommended for sequence generation. No other allocation approach, regardless of its real or supposed sophistication, surpasses the bias prevention and unpredictability of simple randomisation.[231]

Restricted randomisation

Any randomised approach that is not simple randomisation is restricted. Blocked randomisation is the most common form. Other forms, used much less frequently, are methods such as replacement randomisation, biased coin, and urn randomisation.[231]

Blocked randomisation

Blocked randomisation (also called permuted block randomisation) assures that study groups of approximately the same size will be generated when an allocation ratio of 1:1 is used. Blocking can also ensure close balance of the numbers in each group at any time during the trial. After every block of eight participants, for example, four would have been allocated to each trial group.[232] Improved balance comes at the cost of reducing the unpredictability of the sequence. Although the order of interventions varies randomly within each block, a person running the trial could deduce some of the next treatment allocations if they discovered the block size.[233] Blinding the interventions, using larger block sizes, and randomly varying the block size will help to avoid this problem.

Biased coin and urn randomisation

Biased coin designs attain the similar objective as blocked designs without forcing strict equality. They therefore preserve much of the unpredictability associated with simple randomisation. Biased-coin designs alter the allocation ratio during the course of the trial to rectify imbalances that might be occurring.[231] Adaptive biased-coin designs, such as the urn design, vary allocation ratios based on the magnitude of the imbalance. However, these approaches are used infrequently.

Stratified randomisation

Stratification is used to ensure good balance of participant characteristics in each group. Without stratification, study groups may not be well matched for baseline characteristics, such as age and stage of disease, especially in small trials. Such imbalances can be avoided without sacrificing the advantages of randomisation. Stratified randomisation is achieved by performing a separate randomisation procedure within each of two or more strata of participants (eg, categories of age or baseline disease severity), ensuring that the numbers of participants receiving each intervention are closely balanced within each stratum. Stratification requires some form of restriction (eg, blocking within strata) in order to be effective. The number of strata should be limited to avoid over-stratification.[234] Stratification by centre is common in multicentre trials.

Minimisation

Minimisation assures similar distribution of selected participant factors between study groups.[230 235] Randomisation lists are not set up in advance. The first participant is truly randomly allocated; for each subsequent participant, the treatment allocation that minimises the imbalance on the selected factors between groups at that time is identified. That allocation may then be used, or a choice may be made at random with a heavy weighting in favour of the intervention that would minimise imbalance (for example, with a probability of 0.8). The use of a random component is generally preferable.[236] Minimisation has the advantage of making small groups closely similar in terms of participant characteristics at all stages of the trial.

Minimisation offers the only acceptable alternative to randomisation, and some have argued that it is superior.[237] On the other hand, minimisation lacks the theoretical basis for eliminating bias on all known and unknown factors. Nevertheless, in general, trials that use minimisation are considered methodologically equivalent to randomised trials, even when a random element is not incorporated. For SPIRIT, minimisation is considered a restricted randomisation approach without any judgment as to whether it is superior or inferior compared to other restricted randomisation approaches.

chaa006386.indd 16 31/01/2013 10:33:38

Box 3 | Need for a separate document to describe restricted randomisation

If some type of restricted randomisation approach is to be used, in particular blocked randomisation or minimisation, then the knowledge of the specific details could lead to bias.[238 239] For example, if the trial protocol for a two arm, parallel group trial with a 1:1 allocation ratio states that blocked randomisation will be used and the block size will be six, then trial implementers know that the intervention assignments will balance every six participants. Thus, if intervention assignments become known after assignment, knowing the block size will allow trial implementers to predict when equality of the sample sizes will arise. A sequence can be discerned from the pattern of past assignments and then some future assignments could be accurately predicted. For example, if part of a sequence contained two “As” and three “Bs,” trial implementers would know the last assignment in the sequence would be an “A.” If the first three assignments in a sequence contained three “As,” trial implementers would know the last three assignments in that sequence would be three “Bs.” Selection bias could result, regardless of the effectiveness of allocation concealment (Item 16b).

Of course, this is mainly a problem in open label trials, where everyone becomes aware of the intervention after assignment. It can also be a problem in trials where everyone is supposedly blinded (masked), but the blinding is ineffective or the intervention harms provide clues such that treatments can be guessed.

We recommend that trial investigators do not provide full details of a restricted randomisation scheme (including minimisation) in the trial protocol. Knowledge of these details might undermine randomisation by facilitating deciphering of the allocation sequence. Instead, this specific information should be provided in a separate document with restricted access. However, simple randomisation procedures could be reported in detail in the protocol, because simple randomisation is totally unpredictable.

Table 5 | Differences between allocation concealment and blinding (masking) for trials with individual randomisation

	Allocation concealment	Blinding
Definition	Unawareness of the next study group assignment in the allocation sequence	Unawareness of the study group to which trial participants have already been assigned
Purpose	Prevent selection bias by facilitating enrolment of comparable participants in each study group	Prevent ascertainment, performance, and attrition biases by facilitating comparable concomitant care (aside from trial interventions) and evaluation of participants in each study group
Timing of implementation	Before study group assignment	Upon study group assignment and beyond
Who is kept unaware	Trial participants and individuals enrolling them	One or more of the following: trial participants, investigators, care providers, outcome assessors. Other groups: endpoint adjudication committee, data handlers, data analysts
Always possible to implement?	Yes	No

Box 2 defines the various types of randomisation, including minimisation. When restricted randomisation is used, certain details should not appear in the protocol in order to reduce predictability of the random sequence (box 3). The details should instead be described in a separate document that is unavailable to trial implementers. For blocked randomisation, this information would include details on how the blocks will be generated (eg, permuted blocks by a computer random number generator), the block size(s), and whether the block size will be fixed or randomly varied. Specific block size was provided in 14/102 (14%) randomised trial protocols approved by a Danish research ethics committee in 1994-95, potentially compromising allocation concealment.[2] For trials using minimisation, it is also important to state the details in a separate document, including whether random elements will be used.

Allocation—concealment mechanism

Item 16b: Mechanism of implementing the allocation sequence (eg, central telephone; sequentially numbered, opaque, sealed envelopes), describing any steps to conceal the sequence until interventions are assigned

Example

“Participants will be randomised using TENALEA, which is an online, central randomisation service . . . Allocation concealment will be ensured, as the service will not release the randomisation code until the patient has been recruited into the trial, which takes place after all baseline measurements have been completed.”[240]

Explanation

Successful randomisation in practice depends on two interrelated aspects: 1) generation of an unpredictable allocation sequence (Item 16a) and 2) concealment of that sequence until assignment irreversibly occurs.[233 241] The allocation concealment mechanism aims to prevent participants and recruiters from knowing the study group to which the next participant will be assigned. Allocation concealment helps to ensure that a participant’s decision to provide informed consent, or a recruiter’s decision to enrol a participant, is not influenced by knowledge of the group to which they will be allocated if they join the trial.[242] Allocation concealment should not be confused with blinding (masking) (Item 17) (table 5).[243]

Without adequate allocation concealment, even random, unpredictable assignment sequences can be subverted.[233 241] For example, a common practice is to enclose assignments in sequentially numbered, sealed envelopes. However, if the envelopes are not opaque and contents are visible when held up to a light source, or if the envelopes can be unsealed and resealed, then this method of allocation concealment can be corrupted.

Protocols should describe the planned allocation concealment mechanism in sufficient detail to enable assessment of its adequacy. In one study of randomised trial protocols in Denmark, over half did not adequately describe allocation concealment methods.[2] In contrast, central randomisation was stated as the allocation concealment method in all phase III trial protocols initiated in 1968-2003 by a cooperative cancer research group that used extensive protocol review processes.[11] Like sequence generation, inadequate reporting of allocation concealment in trial publications is common and has been associated with inflated effect size estimates.[167 244 245]

Allocation—implementation

Item 16c: Who will generate the allocation sequence, who will enrol participants, and who will assign participants to interventions

Explanation

Based on the risk of bias associated with some methods of sequence generation and inadequate allocation concealment, trial investigators should strive for complete separation of the individuals involved in the steps before enrolment (sequence generation process and allocation concealment mechanism) from those involved in the implementation of study group assignments. When this separation is not possible, it is important for the investigators to

chaa006386.indd 17 31/01/2013 10:33:38

Example

"Randomization

All patients who give consent for participation and who fulfil the inclusion criteria will be randomized. Randomisation will be requested by the staff member responsible for recruitment and clinical interviews from CenTrial [Coordination Centre of Clinical Trials].

In return, CenTrial will send an answer form to the study therapist who is not involved in assessing outcome of the study. This form will include a randomisation number. In every centre closed envelopes with printed randomisation numbers on it are available. For every randomisation number the corresponding code for the therapy group of the randomisation list will be found inside the envelopes. The therapist will open the envelope and will find the treatment condition to be conducted in this patient. The therapist then gives the information about treatment allocation to the patient. Staff responsible for recruitment and symptom ratings is not allowed to receive information about the group allocation.

. . .

The allocation sequence will be generated by the Institute for Medical Biometry (IMB) applying a permuted block design with random blocks stratified by study centre and medication compliance (favourable vs. unfavourable). . . . The block size will be concealed until the primary endpoint will be analysed. Throughout the study, the randomisation will be conducted by CenTrial in order to keep the data management and the statistician blind against the study condition as long as the data bank is open. The randomisation list remains with CenTrial for the whole duration of the study. Thus, randomisation will be conducted without any influence of the principal investigators, raters or therapists."[246]

ensure that the assignment schedule is unpredictable and locked away from even the person who generated it. The protocol should specify who will implement the various stages of the randomisation process, how and where the allocation list will be stored, and mechanisms employed to minimise the possibility that those enrolling and assigning participants will obtain access to the list.

Blinding (masking)

Item 17a: Who will be blinded after assignment to interventions (eg, trial participants, care providers, outcome assessors, data analysts) and how

Example

"Assessments regarding clinical recovery will be conducted by an assessor blind to treatment allocation. The assessor will go through a profound assessment training program . . . Due to the nature of the intervention neither participants nor staff can be blinded to allocation, but are strongly inculcated not to disclose the allocation status of the participant at the follow up assessments. An employee outside the research team will feed data into the computer in separate datasheets so that the researchers can analyse data without having access to information about the allocation."[247]

Explanation

Blinding or masking (the process of keeping the study group assignment hidden after allocation) is commonly used to reduce the risk of bias in clinical trials with two or more study groups.[166 248] Awareness of the intervention assigned to participants can introduce ascertainment bias in the measurement of outcomes, particularly subjective ones (eg, quality of life)[166 167]; performance bias in the decision to discontinue or modify study interventions (eg, dosing changes) (Item 11b), concomitant interventions, or other aspects of care (Item 11d)[229]; and exclusion/attrition bias in the decision to withdraw from the trial or to exclude a participant from the analysis.[249 250] We have elected to use the term "blinding" but acknowledge that others prefer the term "masking" because "blind" also relates to an ophthalmological condition and health outcome.[251 252]

Many groups can be blinded: trial participants, care providers, data collectors, outcome assessors or committees (Item 5d), data analysts,[253] and manuscript writers. Blinding of data monitoring committees is generally discouraged.[254 255]

When blinding of trial participants and care providers is not possible because of obvious differences between the interventions,[256 257] blinding of the outcome assessors can often still be implemented.[17] It may also be possible to blind participants or trial personnel to the study hypothesis in terms of which intervention is considered active. For example, in a trial evaluating light therapy for depression, participants were informed that the study involved testing two different forms of light therapy, whereas the true hypothesis was that bright blue light was considered potentially effective and that dim red light was considered placebo.[258]

Despite its importance, blinding is often poorly described in trial protocols.[3] The protocol should explicitly state who will be blinded to intervention groups—at a minimum, the blinding status of trial participants, care providers, and outcome assessors. Such a description is much preferred over the use of ambiguous terminology such as "single blind" or "double blind."[259 260] Protocols should also describe the comparability of blinded interventions (Item 11a)[150]—for example, similarities in appearance, use of specific flavours to mask a distinctive taste—and the timing of final unblinding of all trial participants (eg, after the creation of a locked analysis data set).[3]

Furthermore, any strategies to reduce the potential for unblinding should be described in the protocol, such as pretrial testing of blinding procedures.[261] The use of a fixed code (versus a unique code for each participant) to denote each study group assignment (eg, A=Group 1; B=Group 2) can be problematic, as the unblinding of one participant will result in the inadvertent loss of blinding for all trial participants.

Some have suggested that the success of blinding be formally tested by asking key trial persons to guess the study group assignment and comparing these responses to what would be expected by chance.[262] However, it is unclear how best to interpret the results of such tests.[263 264] If done, the planned testing methods should be described in the trial protocol.

Blinding (masking)—emergency unblinding

Item 17b: If blinded, circumstances under which unblinding is permissible and procedure for revealing a participant's allocated intervention during the trial

Explanation

Among 58 blinded Danish trials approved in 1994-95, three quarters of protocols described emergency unblinding procedures.[3] Such procedures to reveal the assigned intervention in certain circumstances are intended to increase the safety of trial participants by informing the clinical management of harms or other relevant conditions that arise. A clear protocol description of the conditions and procedures for emergency unblinding helps to prevent unnecessary unblinding; facilitates implementation by trial personnel when indicated; and enables evaluation of the appropriateness of the planned procedures. In some cases (eg, minor, reversible harms), stopping and then cautiously reintroducing the assigned intervention in the affected participant can avoid both unblinding and further harm.

chaa006386.indd 18 31/01/2013 10:33:38

Example

"To maintain the overall quality and legitimacy of the clinical trial, code breaks should occur only in exceptional circumstances when knowledge of the actual treatment is absolutely essential for further management of the patient. Investigators are encouraged to discuss with the Medical Advisor or PHRI [Population Health Research Institute] physician if he/she believes that unblinding is necessary.

If unblinding is deemed to be necessary, the investigator should use the system for emergency unblinding through the PHRI toll-free help line as the main system or through the local emergency number as the back-up system.

The Investigator is encouraged to maintain the blind as far as possible. The actual allocation must NOT be disclosed to the patient and/or other study personnel including other site personnel, monitors, corporate sponsors or project office staff; nor should there be any written or verbal disclosure of the code in any of the corresponding patient documents.

The Investigator must report all code breaks (with reason) as they occur on the corresponding CRF [case report form] page.

Unblinding should not necessarily be a reason for study drug discontinuation."[265]

Section 3c: Methods—data collection, management, and analysis

Data collection methods

Item 18a: Plans for assessment and collection of outcome, baseline, and other trial data, including any related processes to promote data quality (eg, duplicate measurements, training of assessors) and a description of study instruments (eg, questionnaires, laboratory tests) along with their reliability and validity, if known. Reference to where data collection forms can be found, if not in the protocol

Explanation

The validity and reliability of trial data depend on the quality of the data collection methods. The processes of acquiring and recording data often benefit from attention to training of study personnel and use of standardised, pilot tested methods. These should be identical for all study groups, unless precluded by the nature of the intervention.

The choice of methods for outcome assessment can affect study conduct and results.[268-273] Substantially different responses can be obtained for certain outcomes (eg, harms) depending on who answers the questions (eg, the participant or investigator) and how the questions are presented (eg, discrete options or open ended).[269 274-276] Also, when compared to paper based data collection, the use of electronic handheld devices and internet websites has the potential to improve protocol adherence, data accuracy, user acceptability, and timeliness of receiving data.[268 270 271 277]

The quality of data also depends on the reliability, validity, and responsiveness of data collection instruments such as questionnaires[278] or laboratory instruments. Instruments with low inter-rater reliability will reduce

Examples

"Primary outcome

Delirium recognition: In accordance with national guidelines [reference], the study will identify delirium by using the RASS [Richmond Agitation-Sedation Scale] and the CAM-ICU [Confusion Assessment Method for the intensive care unit] on all patients who are admitted directly from the emergency room or transferred from other services to the ICU. Such assessment will be performed after 24 hours of ICU admission and twice daily until discharge from the hospital . . . RASS has excellent inter-rater reliability among adult medical and surgical ICU patients and has excellent validity when compared to a visual analogue scale and other selected sedation scales[reference] . . . The CAM-ICU was chosen because of its practical use in the ICU wards, its acceptable psychometric properties, and based on the recommendation of national guidelines[reference] . . . The CAM-ICU diagnosis of delirium was validated against the DSM-III-R [Diagnostic and Statistical Manual of Mental Disorders, Third Edition—Revised] delirium criteria determined by a psychiatrist and found to have a sensitivity of 97% and a specificity of 92%. [reference] The CAM-ICU has been developed, validated and applied into ICU settings and multiple investigators have used the same method to identify patients with delirium.[reference]

Delirium severity: Since the CAM-ICU does not evaluate delirium severity, we selected the Delirium Rating Scale revised-1998 (DRS-R-98)[reference] . . . The DRS-R-98 was designed to evaluate the breadth of delirium symptoms for phenomenological studies in addition to measuring symptom severity with high sensitivity and specificity . . . The DRS-R-98 is a 16-item clinician-rated scale with anchored items descriptions . . . The DRS-R-98 has excellent inter-rater reliability (intra-class correlation 0.97) and internal consistency (Cronbach's alpha 0.94).[reference]

Secondary outcomes

The study will collect demographic and baseline functional information from the patient's legally authorized representative and/or caregivers. Cognitive function status will be obtained by interviewing the patient's legally authorized representative using the Informant Questionnaire on Cognitive Decline in the Elderly (IQCODE). IQCODE is a questionnaire that can be completed by a relative or other caregiver to determine whether that person has declined in cognitive functioning. The IQCODE lists 26 everyday situations . . . Each situation is rated by the informant for amount of change over the previous 10 years, using a Likert scale ranging from 1-much improved to 5-much worse. The IQCODE has a sensitivity between 69% to 100% and specificity of 80% to 96% for dementia.[reference]

Utilizing the electronic medical record system (RMRS), we will collect several data points of interest at baseline and throughout the study period . . . We have previously defined hospital-related consequences to include: the number of patients with documented falls, use of physical restraints . . . These will be assessed using the RMRS, direct daily observation, and retrospective review of the electronic medical record. This definition of delirium related hospital complications has been previously used and published.[reference]"[266]

"Training and certification plans

. . . Each center's personnel will be trained centrally in the study requirements, standardized measurement of height, weight, and blood pressure, requirements for laboratory specimen collection including morning urine samples, counseling for adherence and the eliciting of information from study participants in a uniform reproducible manner.

. . . The data to be collected and the procedures to be conducted at each visit will be reviewed in detail. Each of the data collection forms and the nature of the required information will be discussed in detail on an item by item basis. Coordinators will learn how to code medications using the WHODrug software and how to code symptoms using the MedDRA software. Entering data forms, responding to data discrepancy queries and general information about obtaining research quality data will also be covered during the training session.

. . .

13.7. Quality Control of the Core Lab

Data from the Core Lab will be securely transmitted in batches and quality controlled in the same manner as Core Coordinating Center data; ie data will be entered and verified in the database on the Cleveland Clinic Foundation SUN with a subset later selected for additional quality control. Appropriate edit checks will be in place at the key entry (database) level.

The Core Lab is to have an internal quality control system established prior to analyzing any FSGS [focal segmental glomerulosclerosis] samples. This system will be outlined in the Manual of Operations for the Core Lab(s) which is prepared and submitted by the Core Lab to the DCC [data coordinating centre] prior to initiating of the study.

At a minimum this system must include:

1) The inclusion of at least two known quality control samples; the reported measurements of the quality control samples must fall within specified ranges in order to be certified as acceptable.

2) Calibration at FDA approved manufacturers' recommended schedules.

13.8. Quality Control of the Biopsy Committee

The chair of the pathology committee will circulate to all of the study pathologists . . . samples [sic] biopsy specimens for evaluation after criteria to establish diagnosis of FSGS has been agreed. This internal review process will serve to ensure common criteria and assessment of biopsy specimens for confirmation of diagnosis of FSGS."[267]

statistical power,[272] while those with low validity will not accurately measure the intended outcome variable. One study found that only 35% (47/133) of randomised trials in acute stroke used a measure with established reliability or validity.[279] Modified versions of validated measurement tools may no longer be considered validated, and use of unpublished measurement scales can introduce bias and inflate treatment effect sizes.[280]

Standard processes should be implemented by local study personnel to enhance data quality and reduce bias by detecting and reducing the amount of missing or incomplete data, inaccuracies, and excessive variability in measurements.[281-285] Examples include standardised training and testing of outcome assessors to promote consistency; tests of the validity or reliability of study instruments; and duplicate data measurements.

A clear protocol description of the data collection process—including the personnel, methods, instruments, and measures to promote data quality—can facilitate implementation and helps protocol reviewers to assess their appropriateness. Inclusion of data collection forms in the protocol (ie, as appendices) is highly recommended, as the way in which data are obtained can substantially affect the results. If not included in the protocol, then a reference to where the forms can be found should be provided. If performed, pilot testing and assessment of reliability and validity of the forms should also be described.

Data collection methods—retention

Item 18b: Plans to promote participant retention and complete follow-up, including list of any outcome data to be collected for participants who discontinue or deviate from intervention protocols

Explanation

Trial investigators must often seek a balance between achieving a sufficiently long follow-up for clinically relevant outcome measurement,[122 288] and a sufficiently short follow-up to decrease attrition and maximise completeness of data collection. Non-retention refers to instances where participants are prematurely "off-study" (ie, consent withdrawn or lost to follow-up) and thus outcome data cannot be obtained from them. The majority of trials will have some degree of non-retention, and the number of these "off-study" participants usually increases with the length of follow-up.[116]

It is desirable to plan ahead for how retention will be promoted in order to prevent missing data and avoid the associated complexities in both the study analysis (Item 20c) and interpretation. Certain methods can improve participant retention,[67 152 289-292] such as financial reimbursement; systematic methods and reminders for contacting patients, scheduling appointments, and monitoring retention; and limiting participant burden related to follow-up visits and procedures (Item 13). A participant who withdraws consent for follow-up assessment of one outcome may be willing to continue with assessments for other outcomes, if given the option.

Non-retention should be distinguished from non-adherence.[293] Non-adherence refers to deviation from intervention protocols (Item 11c) or from the follow-up schedule of assessments (Item 13), but does not mean that the participant is "off-study" and no longer in the trial. Because missing data can be a major threat to trial validity and statistical power, non-adherence should not be an automatic reason for ceasing to collect data from the trial participant prior to study completion. In particular for randomised trials, it is widely recommended that all participants be included in an intention to treat analysis, regardless of adherence (Item 20c).

Examples

"5.2.2 Retention

. . . As with recruitment, retention addresses all levels of participant.

At the parent and student level, study investigators and staff:

- Provide written feedback to all parents of participating students about the results of the "health screenings" . . .
- Maintain interest in the study through materials and mailings . . .
- Send letters to parents and students prior to the final data collection, reminding them of the upcoming data collection and the incentives the students will receive.

At the school level, study investigators and staff:

- Provide periodic communications via newsletters and presentations to inform the school officials/staff, students, and parents about type 2 diabetes, the current status of the study, and plans for the next phase, as well as to acknowledge their support.
- . . .
- Become a presence in the intervention schools to monitor and maintain consistency in implementation, . . . be as flexible as possible with study schedule and proactive in resolving conflicts with schools.
- Provide school administration and faculty with the schedule or grid showing how the intervention fits into the school calendar . . .
- Solicit support from parents, school officials/staff, and teachers . . .
- Provide periodic incentives for school staff and teachers.
- Provide monetary incentives for the schools that increase with each year of the study [table 6]. . ."[286]

"5.4 Infant Evaluations in the Case of Treatment Discontinuation or Study Withdrawal

All randomized infants completing the 18-month evaluation schedule will have fulfilled the infant clinical and laboratory evaluation requirements for the study. . .

All randomized infants who are prematurely discontinued from study drug will be considered *off study drug/on study* and will follow the same schedule of events as those infants who continue study treatment except adherence assessment. All of these infants will be followed through 18 months as scheduled.

Randomized infants prematurely discontinued from the study before the 6-month evaluation will have the following clinical and laboratory evaluations performed, if possible: . . .

- Roche Amplicor HIV-1 DNA PCR [polymerase chain reaction] and cell pellet storage
- Plasma for storage (for NVP [nevirapine] resistance, HIV-1 RNA PCR and NVP concentration)

. . .

Randomized infants prematurely discontinued from the study at any time after the 6-month evaluation will have the following clinical and laboratory evaluations performed, if possible:

. . .

5.5 Participant Retention

Once an infant is enrolled or randomized, the study site will make every reasonable effort to follow the infant for the entire study period . . . It is projected that the rate of loss-to-follow-up on an annual basis will be at most 5% . . . Study site staff are responsible for developing and implementing local standard operating procedures to achieve this level of follow-up.

5.6 Participant Withdrawal

Participants may withdraw from the study for any reason at any time. The investigator also may withdraw participants from the study in order to protect their safety and/or if they are unwilling or unable to comply with required study procedures after consultation with the Protocol Chair, National Institutes of Health (NIH) Medical Officers, Statistical and Data Management Center (SDMC) Protocol Statistician, and Coordinating and Operations Center (CORE) Protocol Specialist.

Participants also may be withdrawn if the study sponsor or government or regulatory authorities terminate the study prior to its planned end date.

Note: Early discontinuation of study product for any reason is not a reason for withdrawal from the study."[287]

chaa006386.indd 20 31/01/2013 10:33:38

Table 6 | Excerpts from table showing compensation provided in study[286]

Who	What	Amount
**School**		
Intervention school	School program enhancement	$2000 in year 1, $3000 in year 2, $4000 in year 3
	Physical education class equipment required to implement intervention	$15 000 over 3 years
	Food service department to defray costs of nutrition intervention	$3000/year
Control school	School program enhancement	$2000 in year 1, $4000 in year 2, $6000 in year 3
**Student**		
All	Return consent form (signed or not)	Gift item worth ~ $5
	Participation in health screening data collection measures and forms	$50 baseline (6th grade), $10 interim (7th grade), $60 end of study (8th grade)
**Family**		
Intervention parents	Focus groups to provide input about family outreach events and activities	$35/year per parent, up to two focus groups per field center, 6-10 participants per focus group

Protocols should describe any retention strategies and define which outcome data will be recorded from protocol non-adherers.[152] Protocols should also detail any plans to record the reasons for non-adherence (eg, discontinuation of intervention due to harms versus lack of efficacy) and non-retention (ie, consent withdrawn; lost to follow-up), as this information can influence the handling of missing data and interpretation of results.[152 294 295]

Data management

Item 19: Plans for data entry, coding, security, and storage, including any related processes to promote data quality (eg, double data entry; range checks for data values). Reference to where details of data management procedures can be found, if not in the protocol

Explanation

Careful planning of data management with appropriate personnel can help to prevent flaws that compromise data validity. The protocol should provide a full description of the data entry and coding processes, along with measures to promote their quality, or provide key elements and a reference to where full information can be found. These details are particularly important for the primary outcome data. The protocol should also document data security measures to prevent unauthorised access to or loss of participant data, as well as plans for data storage

Example

"13.9.2. Data Forms and Data Entry

In the FSGS-CT [focal segmental glomerulosclerosis—clinical trial], all data will be entered electronically. This may be done at a Core Coordinating Center or at the participating site where the data originated. Original study forms will be entered and kept on file at the participating site. A subset will be requested later for quality control; when a form is selected, the participating site staff will pull that form, copy it, and sent [sic] the copy to the DCC [data coordinating center] for re-entry.

. . . Participant files are to be stored in numerical order and stored in a secure and accessible place and manner. Participant files will be maintained in storage for a period of 3 years after completion of the study.

13.9.3. Data Transmission and Editing

The data entry screens will resemble the paper forms approved by the steering committee. Data integrity will be enforced through a variety of mechanisms. Referential data rules, valid values, range checks, and consistency checks against data already stored in the database (ie, longitudinal checks) will be supported. The option to chose [sic] a value from a list of valid codes and a description of what each code means will be available where applicable. Checks will be applied at the time of data entry into a specific field and/or before the data is written (committed) to the database. Modifications to data written to the database will be documented through either the data change system or an inquiry system. Data entered into the database will be retrievable for viewing through the data entry applications. The type of activity that an individual user may undertake is regulated by the privileges associated with his/her user identification code and password.

13.9.4. Data Discrepancy Inquiries and Reports to Core Coordinating Centers

Additional errors will be detected by programs designed to detect missing data or specific errors in the data. These errors will be summarized along with detailed descriptions for each specific problem in Data Query Reports, which will be sent to the Data Managers at the Core Coordinating Centers . . .

The Data Manager who receives the inquiry will respond by checking the original forms for inconsistency, checking other sources to determine the correction, modifying the original (paper) form entering a response to the query. Note that it will be necessary for Data Managers to respond to each inquiry received in order to obtain closure on the queried item.

The Core Coordinating Center and participating site personnel will be responsible for making appropriate corrections to the original paper forms whenever any data item is changed . . . Written documentation of changes will be available via electronic logs and audit trails.

. . .

Biopsy and biochemistry reports will be sent via e-mail when data are received from the Core Lab.

. . .

13.9.5. Security and Back-Up of Data

. . . All forms, diskettes and tapes related to study data will be kept in locked cabinets. Access to the study data will be restricted. In addition, Core Coordinating Centers will only have access to their own center's data. A password system will be utilized to control access . . . These passwords will be changed on a regular basis. All reports prepared by the DCC will be prepared such that no individual subject can be identified.

A complete back up of the primary DCC database will be performed twice a month. These tapes will be stored off-site in a climate-controlled facility and will be retained indefinitely. Incremental data back-ups will be performed on a daily basis. These tapes will be retained for at least one week on-site. Back-ups of periodic data analysis files will also be kept. These tapes will be retained at the off-site location until the Study is completed and the database is on file with NIH [National Institutes of Health]. In addition to the system back-ups, additional measures will be taken to back-up and export the database on a regular basis at the database management level. . .

13.9.6. Study status reports

The DCC will send weekly email reports with information on missing data, missing forms, and missing visits. Personnel at the Core Coordinating Center and the Participating Sites should review these reports for accuracy and report any discrepancies to the DCC.

. . .

13.9.8. Description of Hardware at DCC

A SUN Workstation environment is maintained in the department with a SUN SPARCstation 10 model 41 as the server . . . Primary access to the departments [sic] computing facilities will be through the Internet . . . For maximum programming efficiency, the Oracle database management system and the SAS and BMDP statistical analysis systems will be employed for this study. . . . Oracle facilitates sophisticated integrity checks through a variety of mechanisms including stored procedures, stored triggers, and declarative database integrity—for between table verifications. Oracle allows data checks to be programmed once in the database rather than repeating the same checks among many applications . . . Security is enforced through passwords and may be assigned at different levels to groups and individuals."[267]

chaa006386.indd 21 31/01/2013 10:33:38

(including timeframe) during and after the trial. This information facilitates an assessment of adherence to applicable standards and regulations.

Differences in data entry methods can affect the trial in terms of data accuracy,[268] cost, and efficiency.[271] For example, when compared with paper case report forms, electronic data capture can reduce the time required for data entry, query resolution, and database release by combining data entry with data collection (Item 18a).[271 277] When data are collected on paper forms, data entry can be performed locally or at a central site. Local data entry can enable fast correction of missing or inaccurate data, while central data entry facilitates blinding (masking), standardisation, and training of a core group of data entry personnel.

Raw, non-numeric data are usually coded for ease of data storage, review, tabulation, and analysis. It is important to define standard coding practices to reduce errors and observer variation. When data entry and coding are performed by different individuals, it is particularly important that the personnel use unambiguous, standardised terminology and abbreviations to avoid misinterpretation.

As with data collection (Item 18a), standard processes are often implemented to improve the accuracy of data entry and coding.[281 284] Common examples include double data entry[296]; verification that the data are in the proper format (eg, integer) or within an expected range of values; and independent source document verification of a random subset of data to identify missing or apparently erroneous values. Though widely performed to detect data entry errors, the time and costs of independent double data entry from paper forms need to be weighed against the magnitude of reduction in error rates compared to single-data entry.[297-299]

Statistical methods

The planned methods of statistical analysis should be fully described in the protocol. If certain aspects of the analysis plan cannot be prespecified (eg, the method of handling missing data is contingent on examining patterns of "missingness" before study unblinding), then the planned approach to making the final methodological choices should be outlined. Some trials have a separate document—commonly called a statistical analysis plan (SAP)—that fully details the planned analyses. Any SAP should be described in the protocol, including its key elements and where it can be found. As with the protocol, the SAP should be dated, amendments noted and dated, and the SAP authors provided.

Statistical methods—outcomes

Item 20a: Statistical methods for analysing primary and secondary outcomes. Reference to where other details of the statistical analysis plan can be found, if not in the protocol

Explanation

The protocol should indicate explicitly each intended analysis comparing study groups. An unambiguous, complete, and transparent description of statistical methods facilitates execution, replication, critical appraisal, and the ability to track any changes from the original pre-specified methods.

Example

"The intervention arm (SMS [short message system (text message)]) will be compared against the control (SOC [standard of care]) for all primary analysis. We will use chi-squared test for binary outcomes, and T-test for continuous outcomes. For subgroup analyses, we will use regression methods with appropriate interaction terms (respective subgroup×treatment group). Multivariable analyses will be based on logistic regression . . . for binary outcomes and linear regression for continuous outcomes. We will examine the residual to assess model assumptions and goodness-of-fit. For timed endpoints such as mortality we will use the Kaplan-Meier survival analysis followed by multivariable Cox proportional hazards model for adjusting for baseline variables. We will calculate Relative Risk (RR) and RR Reductions (RRR) with corresponding 95% confidence intervals to compare dichotomous variables, and difference in means will be used for additional analysis of continuous variables. P-values will be reported to four decimal places with p-values less than 0.001 reported as $p < 0.001$. Up-to-date versions of SAS (Cary, NC) and SPSS (Chicago, IL) will be used to conduct analyses. For all tests, we will use 2-sided p-values with alpha≤0.05 level of significance. We will use the Bonferroni method to appropriately adjust the overall level of significance for multiple primary outcomes, and secondary outcomes.

To assess the impact of potential clustering for patients cared by the same clinic, we will use generalized estimating equations [GEE] assuming an exchangeable correlation structure. Table [7] provides a summary of methods of analysis for each variable. Professional academic statisticians (LT, RN) blinded to study groups will conduct all analyses."[47]

Results for the primary outcome can be substantially affected by the choice of analysis methods. When investigators apply more than one analysis strategy for a specific primary outcome, there is potential for inappropriate selective reporting of the most interesting result.[6] The protocol should prespecify the main ("primary") analysis of the primary outcome (Item 12), including the analysis methods to be used for statistical comparisons (Items 20a and 20b); precisely which trial participants will be included (Item 20c); and how missing data will be handled (Item 20c). Additionally, it is helpful to indicate the effect measure (eg, relative risk) and significance level that will be used, as well as the intended use of confidence intervals when presenting results.

The same considerations will often apply equally to prespecified secondary and exploratory outcomes. In some instances, descriptive approaches to evaluating rare outcomes such as adverse events—might be preferred over formal analysis given the lack of power.[300] Adequately powered analyses may require preplanned meta-analyses with results from other studies.

Most trials are affected to some extent by multiplicity issues.[301 302] When multiple statistical comparisons are performed (eg, multiple study groups, outcomes, interim analyses), the risk of false positive (type 1) error is inflated and there is increased potential for selective reporting of favourable comparisons in the final trial report. For trials with more than two study groups, it is important to specify in the protocol which comparisons (of two or more study groups) will be performed and, if relevant, which will be the main comparison of interest. The same principle of specifying the main comparison also applies when there is more than one outcome, including when the same variable

chaa006386.indd 22 31/01/2013 10:33:38

Table 7 | Variables, measures, and methods of analysis (reproduced from original table[47])

Variable/outcome	Hypothesis	Outcome measure	Methods of analysis
1) Primary	Intervention improved outcome from baseline to 6 months		
a) Adherence at 12 months		Percent adherence in previous 30 days >95% [binary]	Chi-squared test
b) Suppression of HIV viral load at 12 months		Viral load ≤400 copies/ml [binary]	Chi-squared test
2) Secondary	improvement occurred	Adherence % (>95%) [binary]	Chi-squared test
Adherence percentage at 12 months			
HIV viral load at 12 months	improvement occurred	Viral load (copies)	T-test
Immune reconstitution (change in CD4 T cellcount from baseline)	improvement occurred	CD4 T-cells/mm^3 (continuous)	T-test
Time to virological failure	improvement occurred	Virological failure after successful suppression	Kaplan-Meier survivalanalysis
Weight gain [lbs] and BMI	improvement occurred	Change in weight (lbs) and BMI [continuous]	T-test
Occurrence of opportunistic infections (OIs)	improvement occurred	Presence of AIDS definingopportunistic infection [binary]	Chi-squared test
Time to reporting of adverse drug events (ADEs)	improvement occurred	Presence of drug-related adverse event [time to event]	Kaplan-Meier survivalanalysis
Deaths (all cause)	improvement occurred	All-cause mortality [binary]	Chi-squared test and Kaplan-Meier survival analysis
SF-12 [short form 12 adapted for regional application in Kiswahili]	improvement occurred	Quality pf [sic] life questionnaire[continuous]	T-test
Satisfaction with care provided	improvement occurred	Questionnaire [continuous]	T-test
Level of disclosure of HIV status	improvement occurred	Disclosed to a family member [binary]	Chi-squared test
Impression of stigma	improvement occurred	Questionnaire [continuous]	T-test
Family dyamics [sic]	improvement occurred	Questionnaire [continuous]	T-test
Employment attendance	improvement occurred	Questionnaire [continuous]	T-test
Household member school attendance	improvement occurred	Questionnaire [continuous]	T-test
Cell phones lost/stolen	improvement occurred	Presence of cellphone [binary]	Poisson regression
Stopped taking HAART [highly active antiretroviral therapy]	improvement occurred	Self-report [binary]	Chi-squared test
Required active tracing for 12 month follow-up	improvement occurred	Field officers [binary]	Chi-squared test
3) Subgroup Analyses:			Regression methods with appropriate interaction term
Urban vs. rural	Distance affectsadherence		
Female vs. male	Sex affects adherence		
Phone ownership (owned vs. shared)	Ownership affectsadherence		
Level of education	Low education affectsadherence		
4) Sensitivity Analyses:	improvement occurred	All outcomes	
a) Per protocol analysis			a) Chi-squared/T-test
b) Adjusting for baseline covariates			b) Multivariable regression
c) clustering among individuals within a clinic			c) GEE

IMPORTANT REMARKS:
- The GEE [generalised estimating equations] [reference] is a technique that allows to specify the correlation structure between patients within a hospital and this approach produces unbiased estimates under the assumption that missing observations will be missing at random. An amended approach of weighted GEE will be employed if missingness is found not to be at random [reference].
- In all analyses results will be expressed as coefficient, standard errors, corresponding 95% and associated p-values.
- Goodness-of-fit will be assessed by examining the residuals for model assumptions and chi-squared test of goodness-of-fit.
- Bonferroni method will be used to adjust the overall level of significance for multiple secondary outcomes.

is measured at several time points (Item 12). Any statistical approaches to account for multiple comparisons and time points should also be described.

Finally, different trial designs dictate the most appropriate analysis plan and any additional relevant information that should be included in the protocol. For example, cluster, factorial, crossover, and within-person randomised trials require specific statistical considerations, such as how clustering will be handled in a cluster randomised trial.

Statistical methods—additional analyses

Item 20b: Methods for any additional analyses (eg, subgroup and adjusted analyses)

Explanation

Subgroup analysis

Subgroup analyses explore whether estimated treatment effects vary significantly between subcategories of trial participants. As these data can help tailor healthcare decisions to individual patients, a modest number of prespecified subgroup analyses can be sensible.

However, subgroup analyses are problematic if they are inappropriately conducted or selectively reported. Subgroup analyses described in protocols or grant applications do not match those reported in subsequent publications for more than two thirds of randomised trials, suggesting that subgroup analyses are often selectively reported or not prespecified.[6 7 305] Post hoc (data driven) analyses have a high risk of spurious findings and are discouraged.[306] Conducting a large number of subgroup comparisons leads to issues of multiplicity, even when all of the comparisons have been pre-specified. Furthermore, when subgroups are based on variables measured after randomisation, the analyses are particularly susceptible to bias.[307]

Preplanned subgroup analyses should be clearly specified in the protocol with respect to the precise baseline variables to be examined, the definition of the subgroup categories (including cut-off boundaries for continuous or ordinal variables), the statistical method to be used, and the hypothesised direction of the subgroup effect based on plausibility.[308 309]

Examples

"We plan to conduct two subgroup analyses, both with strong biological rationale and possible interaction effects. The first will compare hazard ratios of re-operation based upon the degree of soft tissue injury (Gustilo-Anderson Type I/II open fractures vs. Gustilo-Anderson Type IIIA/B open fractures). The second will compare hazard ratios of re-operation between fractures of the upper and lower extremity. We will test if the treatment effects differ with fracture types and extremities by putting their main effect and interaction terms in the Cox regression. For the comparison of pressure, we anticipate that the low/gravity flow will be more effective in the Type IIIA-B open fracture than in the Type I/II open fracture, and be more effective in the upper extremity than the lower extremity. For the comparison of solution, we anticipate that soap will do better in the Type IIIA-B open fracture than in the Type I/II open fracture, and better in the upper extremity than the lower extremity."[303]

"A secondary analysis of the primary endpoint will adjust for those pre-randomization variables which might reasonably be expected to be predictive of favorable outcomes. Generalized linear models will be used to model the proportion of subjects with neurologically intact (MRS ≤ 3 [Modified Rankin Score]) survival to hospital discharge by ITD [impedance threshold device]/sham device group adjusted for site (dummy variables modeling the 11 ROC [Resuscitation Outcomes Consortium] sites), patient sex, patient age (continuous variable), witness status (dummy variables modeling the three categories of unwitnessed arrest, non-EMS [emergency medical services] witnessed arrest, and EMS witnessed arrest), location of arrest (public versus non-public), time or response (continuous variable modeling minutes between call to 911 and arrival of EMS providers on scene), presenting rhythm (dummy variables modeling asystole, PEA [pulseless electrical activity], VT/VF [ventricular tachycardia/fibrillation], or unknown), and treatment assignment in the Analyze Late vs. Analyze Early intervention. The test statistic used to assess any benefit of the ITD relative to the sham device will be computed as the generalized linear model regression coefficient divided by the estimated "robust" standard error based on the Huber- White sandwich estimator[reference] in order to account for within group variability which might depart from the classical assumptions. Statistical inference will be based on one-sided P values and 95% confidence intervals which adjust for the stopping rule used for the primary analysis."[304]

Adjusted analysis

Some trials prespecify adjusted analyses to account for imbalances between study groups (eg, chance imbalance across study groups in small trials), improve power, or account for a known prognostic variable. Adjustment is often recommended for any variables used in the allocation process (eg, in stratified randomisation), on the principle that the analysis strategy should match the design.[310] Most trial protocols and publications do not adequately address issues of adjustment, particularly the description of variables.[6 310]

It is important that trial investigators indicate in the protocol if there is an intention to perform or consider adjusted analyses, explicitly specifying any variables for adjustment and how continuous variables will be handled. When both unadjusted and adjusted analyses are intended, the main analysis should be identified (Item 20a). It may not always be clear, in advance, which variables will be important for adjustment. In such situations, the objective criteria to be used to select variables should be prespecified. As with subgroup analyses, adjustment variables based on post-randomisation data rather than baseline data can introduce bias.[311 312]

Statistical methods—analysis population and missing data

Item 20c: Definition of analysis population relating to protocol non-adherence (eg, as randomised analysis), and any statistical methods to handle missing data (eg, multiple imputation)

Explanation

In order to preserve the unique benefit of randomisation as a mechanism to avoid selection bias, an "as randomised" analysis retains participants in the group to which they were originally allocated. To prevent attrition bias, outcome data obtained from all participants are included in the data analysis, regardless of protocol adherence (Items 11c and 18b).[249 250] These two conditions (ie, all participants, as randomised) define an "intention to treat" analysis, which is widely recommended as the preferred analysis strategy.[17]

Some trialists use other types of data analyses (commonly labelled as "modified intention to treat" or "per protocol") that exclude data from certain participants—such as those who are found to be ineligible after randomisation or who deviate from the intervention or follow-up protocols. This exclusion of data from protocol non-adherers can introduce bias, particularly if the frequency of and the reasons for non-adherence vary between the study groups.[314 315] In some trials, the participants to be included in the analysis will vary by outcome—for example, analysis

Example

"Nevertheless, we propose to test non-inferiority using two analysis sets; the intention-to-treat set, considering all patients as randomized regardless of whether they received the randomized treatment, and the "per protocol" analysis set. Criteria for determining the "per protocol" group assignment would be established by the Steering Committee and approved by the PSMB [performance and safety monitoring board] before the trial begins. Given our expectation that very few patients will crossover or be lost to follow-up, these analyses should agree very closely. We propose declaring medical management non-inferior to interventional therapy, only if shown to be non-inferior using both the "intention to treat" and "per protocol" analysis sets.

. . .

10.4.7 Imputation Procedure for Missing Data

While the analysis of the primary endpoint (death or stroke) will be based on a log-rank test and, therefore, not affected by patient withdrawals (as they will be censored) provided that dropping out is unrelated to prognosis; other outcomes, such as the Rankin Score at five years post-randomization, could be missing for patients who withdraw from the trial. We will report reasons for withdrawal for each randomization group and compare the reasons qualitatively . . . The effect that any missing data might have on results will be assessed via sensitivity analysis of augmented data sets. Dropouts (essentially, participants who withdraw consent for continued follow-up) will be included in the analysis by modern imputation methods for missing data.

The main feature of the approach is the creation of a set of clinically reasonable imputations for the respective outcome for each dropout. This will be accomplished using a set of repeated imputations created by predictive models based on the majority of participants with complete data. The imputation models will reflect uncertainty in the modeling process and inherent variability in patient outcomes, as reflected in the complete data.

After the imputations are completed, all of the data (complete and imputed) will be combined and the analysis performed for each imputed-and-completed dataset. Rubin's method of multiple (ie, repeated) imputation will be used to estimate treatment effect. We propose to use 15 datasets (an odd number to allow use of one of the datasets to represent the median analytic result).

These methods are preferable to simple mean imputation, or simple "best-worst" or "worst-worst" imputation, because the categorization of patients into clinically meaningful subgroups, and the imputation of their missing data by appropriately different models, accords well with best clinical judgment concerning the likely outcomes of the dropouts, and therefore will enhance the trial's results."[313]

chaa006386.indd 24 31/01/2013 10:33:39

of harms (adverse events) is sometimes restricted to participants who received the intervention, so that absence or occurrence of harm is not attributed to a treatment that was never received.

Protocols should explicitly describe which participants will be included in the main analyses (eg, all randomised participants, regardless of protocol adherence) and define the study group in which they will be analysed (eg, as randomised). In one cohort of randomised trials approved in 1994-5, this information was missing in half of the protocols.[6] The ambiguous use of labels such as "intention to treat" or "per protocol" should be avoided unless they are fully defined in the protocol.[6 314] Most analyses labelled as "intention to treat" do not actually adhere to its definition because of missing data or exclusion of participants who do not meet certain post-randomisation criteria (eg, specific level of adherence to intervention).[6 316] Other ambiguous labels such as "modified intention to treat" are also variably defined from one trial to another.[314]

In addition to defining the analysis population, it is necessary to address the problem of missing data in the protocol. Most trials have some degree of missing data,[317 318] which can introduce bias depending on the pattern of "missingness" (eg, not missing at random). Strategies to maximise follow-up and prevent missing data, as well as the recording of reasons for missing data, are thus important to develop and document (Item 18b).[152]

The protocol should also state how missing data will be handled in the analysis and detail any planned methods to impute (estimate) missing outcome data, including which variables will be used in the imputation process (if applicable).[152] Different statistical approaches can lead to different results and conclusions,[317 319] but one study found that only 23% of trial protocols specified the planned statistical methods to account for missing data.[6]

Imputation of missing data allows the analysis to conform to intention to treat analysis but requires strong assumptions that are untestable and may be hard to justify.[152 318 320 321] Methods of multiple imputation are more complex but are widely preferred to single imputation methods (eg, last observation carried forward; baseline observation carried forward), as the latter introduce greater bias and produce confidence intervals that are too narrow.[152 320-322] Specific issues arise when outcome data are missing for crossover or cluster randomised trials.[323] Finally, sensitivity analyses are highly recommended to assess the robustness of trial results under different methods of handling missing data.[152 324]

Section 3d: Methods—monitoring

Data monitoring—formal committee

Item 21a: Composition of data monitoring committee (DMC); summary of its role and reporting structure; statement of whether it is independent from the sponsor and competing interests; and reference to where further details about its charter can be found, if not in the protocol. Alternatively, an explanation of why a DMC is not needed

Explanation

For some trials, there are important reasons for periodic inspection of the accumulating outcome data by study group. In principle, a trial should be modified or discontinued when the accumulated data have sufficiently disturbed the clinical equipoise that justified the initiation of the trial. Data monitoring can also inform aspects of trial conduct, such as recruitment, and identify the need to make adjustments.

The decision to have a data monitoring committee (DMC) will be influenced by local standards. While certain trials warrant some form of data monitoring, many do not need a formal committee,[326] such as trials with a short duration or known minimal risks. A DMC was described in 65% (98/150) of cancer trial protocols with time-to-event outcomes in Italy in 2000-5,[327] and in 17% (12/70) of protocols for Danish randomised trials approved in 1994-5.[6] About 40% of clinical trials registered on ClinicalTrials.gov from 2007-2010 reported having a DMC.[328] The protocol should either state that there will be a DMC and provide further details, as discussed below, or indicate that there will not be a DMC, preferably with reasons.

When formal data monitoring is performed, it is often done by a DMC consisting of members from a variety of disciplines.[254 329] The primary role of a DMC is to periodically review the accumulating data and determine if a trial should be modified or discontinued. The DMC does not usually have executive power; rather, it communicates the outcome of its deliberations to the trial steering committee or sponsor.

Independence, in particular from the sponsor and trial investigators, is a key characteristic of the DMC and can be broadly defined as the committee comprising members who are "completely uninvolved in the running of the trial and who cannot be unfairly influenced (either directly

Example

"Appendix 3. Charter and responsibilities of the Data Monitoring Committee

A Data Monitoring Committee (DMC) has been established. The DMC is independent of the study organisers. During the period of recruitment to the study, interim analyses will be supplied, in strict confidence, to the DMC, together with any other analyses that the committee may request. This may include analyses of data from other comparable trials. In the light of these interim analyses, the DMC will advise the TSC [trial steering committee] if, in its view:

a) the active intervention has been proved, beyond reasonable doubt*, to be different from the control (standard management) for all or some types of participants, and

b) the evidence on the economic outcomes is sufficient to guide a decision from health care providers regarding recommendation of early lens extraction for PACG [primary angle closure glaucoma].

The TSC can then decide whether or not to modify intake to the trial. Unless this happens, however, the TSC, PMG [project management group], clinical collaborators and study office staff (except those who supply the confidential analyses) will remain ignorant of the interim results.

The frequency of interim analyses will depend on the judgement of the Chair of the DMC, in consultation with the TSC. However, we anticipate that there might be three interim analyses and one final analysis.

The Chair is Mr *D.G.-H.*, with Dr *D.C.*, and Professor *B.D.* Terms of reference for the DMC are available on request from the EAGLE [Effectiveness in Angle Closure Glaucoma of Lens Extraction] study office.

*Appropriate criteria for proof beyond reasonable doubt cannot be specified precisely. A difference of at least three standard deviation [sic] in the interim analysis of a major endpoint may be needed to justify halting, or modifying, such a study prematurely.[reference]"[325]

chaa006386.indd 25 31/01/2013 10:33:39

or indirectly) by people, or institutions, involved in the trial."[254] DMC members are usually required to declare any competing interests (Item 28). Among the 12 trial protocols that described a DMC and were approved in Denmark in 1994-5,[6] four explicitly stated that the DMC was independent from the sponsor and investigators; three had non-independent DMCs; and independence was unclear for the remaining five protocols.

The protocol should name the chair and members of the DMC. If the members are not yet known, the protocol can indicate the intended size and characteristics of the membership until further details are available. The protocol should also indicate the DMC's roles and responsibilities, planned method of functioning, and degree of independence from those conducting, sponsoring, or funding the trial.[254 330 331] A charter is recommended for detailing this information[331]; if this charter is not appended to the protocol, the protocol should indicate whether a charter exists or will be developed, and if so, where it can be accessed.

Data monitoring—interim analysis

Item 21b: Description of any interim analyses and stopping guidelines, including who will have access to these interim results and make the final decision to terminate the trial

Example

"Premature termination of the study

An interim-analysis is performed on the primary endpoint when 50% of patients have been randomised and have completed the 6 months follow-up. The interim-analysis is performed by an independent statistician, blinded for the treatment allocation. The statistician will report to the independent DSMC [data and safety monitoring committee]. The DSMC will have unblinded access to all data and will discuss the results of the interim-analysis with the steering committee in a joint meeting. The steering committee decides on the continuation of the trial and will report to the central ethics committee. The Peto approach is used: the trial will be ended using symmetric stopping boundaries at P < 0.001 [reference]. The trial will not be stopped in case of futility, unless the DSMC during the course of safety monitoring advices [sic] otherwise. In this case DSMC will discuss potential stopping for futility with the trial steering committee."[332]

Explanation

Interim analyses can be conducted as part of an adaptive trial design to formally monitor the accumulating data in clinical trials. They are generally performed in trials that have a DMC, longer duration of recruitment, and potentially serious outcomes. Interim analyses were described in 71% (106/150) of cancer trial protocols with time-to-event outcomes in Italy in 2000-5,[327] and in 19% (13/70) of protocols for Danish randomised trials approved in 1994-5.[6] The results of these analyses, along with non-statistical criteria, can be part of a stopping guideline that helps inform whether the trial should be continued, modified, or halted earlier than intended for benefit, harm, or futility. Criteria for stopping for harm are often different from those for benefit and might not employ a formal statistical criterion.[333] Stopping for futility occurs in instances where, if the study were to continue, it is unlikely that an important effect would be seen (ie, low chance of rejecting null hypothesis). Multiple analyses of the accumulating data increase the risk of a false positive (type I) error, and various statistical strategies have been developed to compensate for this inflated risk.[254 333-335] Aside from informing stopping guidelines, prespecified interim analyses can be used for other trial adaptations such as sample size re-estimation, alteration to the proportion of participants allocated to each study group, and changes to eligibility criteria.[111]

A complete description of any interim analysis plan, even if it is only to be performed at the request of an oversight body (eg, DMC), should be provided in the protocol—including the statistical methods, who will perform the analyses, and when they will be conducted (timing and indications). If applicable, details should also be provided about the decision criteria—statistical or other—that will be adopted to judge the interim results as part of a guideline for early stopping or other adaptations. Among 86 protocols for randomised trials with a time-to-event cancer outcome that proposed efficacy interim analyses, all stated the planned timing of the analyses, 91% specified the overall reason to be used for stopping (eg, superiority, futility), and 94% detailed the statistical approach.[327]

In addition, it is important to state who will see the outcome data while the trial is ongoing, whether these individuals will remain blinded (masked) to study groups, and how the integrity of the trial implementation will be protected (eg, maintaining blinding) when any adaptations to the trial are made. A third of protocols for industry initiated randomised trials receiving Danish ethics approval in 1994-95 stated that the sponsor had access to accumulating trial data, which can introduce potential bias due to competing interests.[10] Finally, the protocol should specify who has the ultimate authority to stop or modify the trial—eg, the principal investigator, trial steering committee, or sponsor.

Harms

Item 22: Plans for collecting, assessing, reporting, and managing solicited and spontaneously reported adverse events and other unintended effects of trial interventions or trial conduct

Explanation

Evaluation of harms has a key role in monitoring the condition of participants during a trial and in enabling appropriate management of adverse events. Documentation of trial related adverse events also informs clinical practice and the conduct of ongoing and future studies. We use the term "harms" instead of "safety" to better reflect the negative effects of interventions.[300] An adverse event refers to an untoward occurrence during the trial, which may or may not be causally related to the intervention or other aspects of trial participation.[300 336] This definition includes unfavourable changes in symptoms, signs, laboratory values, or health conditions. In the context of clinical trials, it can be difficult to attribute causation for a given adverse event. An adverse effect is a type of adverse event that can be attributed to the intervention.

Harms can be specified as primary or secondary outcomes (Item 12) or can be assessed as part of routine monitoring. To the extent possible, distinctions should be made between adverse events that are anticipated versus unanticipated, and solicited versus unsolicited, because expectation can influence the number and perceived severity of recorded events.

Example

"Secondary outcomes

. . . In our study an adverse event will be defined as any untoward medical occurrence in a subject without regard to the possibility of a causal relationship. Adverse events will be collected after the subject has provided consent and enrolled in the study. If a subject experiences an adverse event after the informed consent document is signed (entry) but the subject has not started to receive study intervention, the event will be reported as not related to study drug. All adverse events occurring after entry into the study and until hospital discharge will be recorded. An adverse event that meets the criteria for a serious adverse event (SAE) between study enrollment and hospital discharge will be reported to the local IRB [institutional review board] as an SAE. If haloperidol is discontinued as a result of an adverse event, study personnel will document the circumstances and data leading to discontinuation of treatment. A serious adverse event for this study is any untoward medical occurrence that is believed by the investigators to be causally related to study-drug and results in any of the following: Life-threatening condition (that is, immediate risk of death); severe or permanent disability, prolonged hospitalization, or a significant hazard as determined by the data safety monitoring board. Serious adverse events occurring after a subject is discontinued from the study will NOT be reported unless the investigators feels that the event may have been caused by the study drug or a protocol procedure. Investigators will determine relatedness of an event to study drug based on a temporal relationship to the study drug, as well as whether the event is unexpected or unexplained given the subject's clinical course, previous medical conditions, and concomitant medications.

. . . The study will monitor for the following movement-related adverse effects daily through patient examination and chart review: dystonia, akathisia, pseudoparkinsonism, akinesia, and neuroleptic malignant syndrome. Study personnel will use the Simpson-Angus [reference] and Barnes Akathisia [reference] scales to monitor movement-related effects.

. . .

For secondary outcomes, binary measures, eg mortality and complications, logistic regression will be used to test the intervention effect, controlling for covariates when appropriate . . ."[266]

For example, providing statements in the informed consent process about the possibility of a particular adverse effect or using structured, as opposed to open ended, questionnaires for data collection, can increase the reporting of specific events ("priming").[269 337-339] The timeframe for recording adverse events can also affect the type of data obtained.[340 341]

The protocol should describe the procedures for and frequency of harms data collection, the overall surveillance timeframe, any instruments to be used, and their validity and reliability, if known. Substantial discrepancies have been observed between protocol specified plans for adverse event collection and reporting, and what is described in final publications.[5] Although trials are often not powered to detect important differences in rates of uncommon adverse events, it is also important to describe plans for data analysis, including formal hypothesis testing or descriptive statistics.[300 342]

Finally, the protocol should address the reporting of harms to relevant groups (eg, sponsor, research ethics committee/institutional review board, data monitoring committee, regulatory agency), which is an important process that is subject to local regulation.[343] Key considerations include the severity of the adverse event, determination of potential causality, and whether it represents an unexpected or anticipated event. For multicentre studies, procedures and timing should be outlined for central collection, evaluation, and reporting of pooled harms data.

Example

"11.4 Data Monitoring and Quality Assurance

Through the combination of our web-based, instantaneous electronic validation, the DCC's [data coordinating centre] daily visual cross-validation of the data for complex errors, and regular on-site monitoring, the quality and completeness of the data will be reflective of the state of the art in clinical trials.

Both the European and US DCCs will conduct monitoring of source documents via fax at all enrolling ARUBA [A Randomised trial of Unruptured Brain Arteriovenous malformations] sites and will conduct at least one on-site monitoring visit per year over the course of the study at 100% of clinical sites (with repeat visits to sites where performance is a concern). Monitoring of European study sites will be assured by the European Coordinating Center (Paris). The primary objectives of the DCC during the on-site visits are to educate, support and solve problems. The monitors will discuss the protocol in detail and identify and clarify any areas of weakness. At the start of the trial, the monitors will conduct a tutorial on the web-based data entry system. The coordinators will practice entering data so that the monitors can confirm that the coordinators are proficient in all aspects of data entry, query response, and communication with the DCC. They will audit the overall quality and completeness of the data, examine source documents, interview investigators and coordinators, and confirm that the clinical center has complied with the requirements of the protocol. The monitors will verify that all adverse events were documented in the correct format, and are consistent with protocol definition.

The monitors will review the source documents as needed, to determine whether the data reported in the Web-based system are complete and accurate. Source documents are defined as medical charts, associated reports and records including initial hospital admission report . . .

The monitors will confirm that the regulatory binder is complete and that all associated documents are up to date. The regulatory binder should include the protocol and informed consent (all revisions), IRB [institutional review board] approvals for all of the above documents, IRB correspondence, case report forms, investigator's agreements . . .

Scheduling monitoring visits will be a function of patient enrollment, site status and other commitments. The DCC will notify the site in writing at least three weeks prior to a scheduled visit. The investigators must be available to meet with the monitors. Although notification of the visits will include the list of patients scheduled to be reviewed, the monitors reserve the right to review additional ARUBA patients.

If a problem is identified during the visit (ie, poor communication with the DCC, inadequate or insufficient staff to conduct the study, missing study documents) the monitor will assist the site in resolving the issues. Some issues may require input from the Operations Committee, Steering Committee or one of the principal investigators.

The focus of the visit/electronic monitoring will be on source document review and confirmation of adverse events. The monitor will verify the following variables for all patients: initials, date of birth, sex, signed informed consent, eligibility criteria, date of randomization, treatment assignment, adverse events, and endpoints . . ."[313]

Auditing

Item 23: Frequency and procedures for auditing trial conduct, if any, and whether the process will be independent from investigators and the sponsor

Explanation

Auditing involves periodic independent review of core trial processes and documents. It is distinct from routine day-to-day measures to promote data quality (Items 18a and 19). Auditing is intended to preserve the integrity of the trial by independently verifying a variety of processes and prompting corrective action if necessary. The processes reviewed can relate to participant enrolment, consent, eligibility, and allocation to study groups; adherence to trial interventions and policies to protect

participants, including reporting of harms (Item 22); and completeness, accuracy, and timeliness of data collection. In addition, an audit can verify adherence to applicable policies such as the International Conference on Harmonisation *Good Clinical Practice* and regulatory agency guidelines.[160]

In multicentre trials, auditing is usually considered both overall and for each recruiting centre. Audits can be done by exploring the trial dataset or performing site visits. Audits might be initially conducted across all sites, and subsequently conducted using a risk based approach that focuses, for example, on sites that have the highest enrolment rates, large numbers of withdrawals, or atypical (low or high) numbers of reported adverse events.

If auditing is planned, the procedures and anticipated frequency should be outlined in the protocol, including a description of the personnel involved and their degree of independence from the trial investigators and sponsor. If procedures are further detailed elsewhere (eg, audit manual), then the protocol should reference where the full details can be obtained.

Section 4: Ethics and dissemination

Research ethics approval

Item 24: Plans for seeking research ethics committee/institutional review board (REC/IRB) approval

Example

"This protocol and the template informed consent forms contained in Appendix II will be reviewed and approved by the sponsor and the applicable IRBs/ECs [institutional review boards/ethical committees] with respect to scientific content and compliance with applicable research and human subjects regulations. . . .

The protocol, site-specific informed consent forms (local language and English versions), participant education and recruitment materials, and other requested documents—and any subsequent modifications — also will be reviewed and approved by the ethical review bodies. . .

Subsequent to initial review and approval, the responsible local Institutional Review Boards/Ethical Committees (IRBs/ECs) will review the protocol at least annually. The Investigator will make safety and progress reports to the IRBs/ECs at least annually and within three months of study termination or completion at his/her site. These reports will include the total number of participants enrolled . . . and summaries of each DSMB [data safety and monitoring board] review of safety and/or efficacy."[287]

Explanation

A universal requirement for the ethical conduct of clinical research is the review and approval of the research protocol by qualified individuals who are not associated with the research team and have no disqualifying competing interests as reviewers.[1] The review is typically conducted by a formal REC/IRB in accordance with jurisdictional policy. Despite the importance of ethics review, approval by a REC/IRB is not always obtained. Among 767 trials published in leading general medical journals from 1993-95, 37 authors (5%) disclosed that such approval had not been sought for their trials.[344] The protocol should document where approval has been obtained, or outline plans to seek such approval.

Protocol amendments

Item 25: Plans for communicating important protocol modifications (eg, changes to eligibility criteria, outcomes, analyses) to relevant parties (eg, investigators, REC/IRBs, trial participants, trial registries, journals, regulators)

Example

"13.10 Modification of the Protocol

Any modifications to the protocol which may impact on the conduct of the study, potential benefit of the patient or may affect patient safety, including changes of study objectives, study design, patient population, sample sizes, study procedures, or significant administrative aspects will require a formal amendment to the protocol. Such amendment will be agreed upon by BCIRG [Breast Cancer International Research Group] and Aventis, and approved by the Ethics Committee/IRB [institutional review board] prior to implementation and notified to the health authorities in accordance with local regulations.

Administrative changes of the protocol are minor corrections and/or clarifications that have no effect on the way the study is to be conducted. These administrative changes will be agreed upon by BCIRG and Aventis, and will be documented in a memorandum. The Ethics Committee/IRB may be notified of administrative changes at the discretion of BCIRG."[345]

Explanation

After initial ethics approval, about half of trials have subsequent protocol amendments submitted to the REC/IRB.[125 346 347] While some amendments may be unavoidable, a study of pharmaceutical industry trials found that according to the sponsors, a third of amendments could have been prevented with greater attention to key issues during protocol development.[346] Substantive amendments can generate challenges to data analysis and interpretation if they occur part way through the trial (eg, changes in eligibility criteria),[348] and can introduce bias if the changes are made based on the trial data.[173-176] The implementation and communication of amendments are also burdensome and potentially costly.[346]

Numerous studies have revealed substantive changes between prespecified methods (eg, as stated in approved protocols, registries, or regulatory agency submissions) and those described in trial publications, including changes to primary outcomes,[12 172-176] sample size calculations,[6] eligibility criteria,[125 133 134] as well as methods of allocation concealment,[2] blinding,[3] and statistical analysis.[6-8 174] These substantive modifications are rarely acknowledged in the final trial reports, providing an inaccurate impression of trial integrity.

It is important that substantive protocol amendments be reviewed by an independent party, such as the REC/IRB, and transparently described in trial reports. The notion of "substantive" is variably defined by authorities, but in general refers to a protocol amendment that can affect the safety of trial participants or the scientific validity, scope, or ethical rigour of the trial.[349 350] To reflect the degree of oversight for the trial and adherence to applicable regulation, the protocol should describe the process for making amendments, including who will be responsible for the decision to amend the protocol and how substantive changes will be communicated to relevant stakeholders (eg, REC/IRBs, trial registries, regulatory agencies). Version

chaa006386.indd 28 31/01/2013 10:33:39

control using protocol identifiers and dates (Item 3), as well as a list of amendments, can help to track the history of amendments and identify the most recent protocol version.

Consent or assent

Item 26a: Who will obtain informed consent or assent from potential trial participants or authorised surrogates, and how (see Item 32)

> **Example**
>
> "... Trained Research Nurses will introduce the trial to patients who will be shown a video regarding the main aspects of the trial. Patients will also receive information sheets. Research Nurses will discuss the trial with patients in light of the information provided in the video and information sheets. Patients will then be able to have an informed discussion with the participating consultant. Research Nurses will obtain written consent from patients willing to participate in the trial. Information sheets and consent forms are provided for all parents involved in the trial however these have been amended accordingly in order to provide separate information sheets and consent form [sic] which are suitable for children and teenagers. All information sheets, consent forms and the video transcript have been translated into Bengali, Punjabi, Gujarati, and Urdu. There are also separate information sheets and consent forms for the cohort group."[351]

Explanation

The notion of acquiring informed consent involves the presentation of comprehensible information about the research to potential participants, confirmation that they understand the research, and assurance that their agreement to participate is voluntary. The process typically involves discussion between the potential participant and an individual knowledgeable about the research; the presentation of written material (eg, information leaflet or consent document); and the opportunity for potential participants to ask questions. Surveys of trial investigators reveal that appropriate informed consent is not always obtained.[344 352]

The content, quantity, and mode of delivery of consent information can affect trial recruitment, participant comprehension, anxiety, retention rates, and recruitment cos ts.[68 114 218 292 353-355] We recommend that a model consent or assent form be provided as a protocol appendix (Item 32). Assent represents a minor's affirmative agreement to participate in the trial, which typically involves signing a document that provides age appropriate information about the study.

The protocol should include details of the consent process as well as the status, experience, and training (if applicable) of the research team members who will conduct it. In paediatric research, regulations may stipulate obtaining affirmative assent for participation from children above a certain age.[356] The protocol should then describe how pertinent information will be provided to potential participants and how their understanding and assent will be ascertained. When potential participants lack decisional capacity for reasons other than young age (eg, mental status), and proxy consent can be obtained from a legally-authorised representative, the protocol should describe who will determine an individual's decisional capacity, whether a formal capacity instrument will be utilised, and how the individual's informed agreement to continue participation will be secured should they regain decisional capacity. For certain trials, such as cluster randomised trials, it may not be possible to acquire individual informed consent from participants before randomisation, and the consent process may be modified or waived. An explanation should be provided in the protocol in these instances.[357]

Consent or assent—ancillary studies

Item 26b: Additional consent provisions for collection and use of participant data and biological specimens in ancillary studies, if applicable

> **Example**
>
> **"6.4.1. Samples for Biorepositories**
>
> Additional biological samples will be obtained to be stored for use in future studies of the pathobiology of FSGS [focal segmental glomerulosclerosis]. A materials consent will be obtained to specifically address the collection of these ... urine, serum and plasma specimens ...
>
> **14.3.4. Instructions for Preparation of Requests for an Ancillary Study**
>
> ... A signed consent must be obtained from every participant in the ancillary study, if the data collection/request is not covered in the original informed consent process for the main FSGS Clinical Trial.
>
> ...
>
> A copy of the IRB [institutional review board] letter for the ancillary study should be sent to the DCC [data coordinating centre]. If a separate consent form is required for the ancillary study, a copy of the signed ancillary study consent form for each study participant must be included in the FSGS-CT [clinical trial] record. A data file tracking all signed ancillary consent forms must be maintained by the ancillary study and an electronic copy of that file must be delivered to the FSGS-CT DCC."[267]

Explanation

Ancillary studies involve the collection or derivation of data for purposes that are separate from the main trial. The acquisition and storage of data and biological specimens for ancillary studies is increasingly common in the context of clinical trials (Item 33). Specimens may be used for a specified subset of studies or for submission to biorepositories for future specified or unspecified research.

Ancillary studies have additional processes and considerations relating to consent, which should be detailed in the protocol. Guidance for the creation of a simplified informed consent document for biobanking is available.[358] Participants can be given several options to consider with respect to their participation in ancillary research: consent for the use of their data and specimens in specified protocols; consent for use in future research unrelated to the clinical condition under study; consent for submission to an unrelated biorepository; and consent to be contacted by trial investigators for further informational and consent-related purposes. This is commonly referred to as tiered consent. Participants should also be informed about whether their withdrawal from the ancillary research is possible (eg, the data and specimens are coded and identifiable); what withdrawal means in this context (eg, used specimens and data derived from them cannot be withdrawn); and what information derived from the specimen related research will be provided to them, if any.

chaa006386.indd 29 31/01/2013 10:33:39

Confidentiality

Item 27: How personal information about potential and enrolled participants will be collected, shared, and maintained in order to protect confidentiality before, during, and after the trial

Example

"8.5 Confidentiality
All study-related information will be stored securely at the study site. All participant information will be stored in locked file cabinets in areas with limited access. All laboratory specimens, reports, data collection, process, and administrative forms will be identified by a coded ID [identification] number only to maintain participant confidentiality. All records that contain names or other personal identifiers, such as locator forms and informed consent forms, will be stored separately from study records identified by code number. All local databases will be secured with password-protected access systems. Forms, lists, logbooks, appointment books, and any other listings that link participant ID numbers to other identifying information will be stored in a separate, locked file in an area with limited access.

All HIV test results will be kept strictly confidential, all counseling and blood draws will be conducted in private rooms, and study staff will be required to sign agreements to preserve the confidentiality of all participants. Study staff will never inform network members of the serostatus of other members of their group, but counselors will provide general messages about the prevalence of HIV in the study population in the interests of emphasizing harm reduction.

Participants' study information will not be released outside of the study without the written permission of the participant, except as necessary for monitoring by NIAID [National Institute of Allergy and Infectious Diseases] and/or its contractors . . . representatives of the HPTN CORE [HIV Prevention Trials Network Coordinating and Operations Center] . . . and US or in-country government and regulatory authorities."[359]

Explanation

Personal information about participants is acquired during the process of trial recruitment, eligibility screening, and data collection. Much of this information consists of private details over which people customarily wish to maintain control, such as their health status, personal genotype, and social and family history.

The protocol should describe the means whereby personal information is collected, kept secure, and maintained. In general, this involves: 1) the creation of coded, depersonalised data where the participant's identifying information is replaced by an unrelated sequence of characters; 2) secure maintenance of the data and the linking code in separate locations using encrypted digital files within password protected folders and storage media; and 3) limiting access to the minimum number of individuals necessary for quality control, audit, and analysis. The protocol should also describe how the confidentiality of data will be preserved when the data are transmitted to sponsors and coinvestigators (eg, virtual private network internet transmission).

Declaration of interests

Item 28: Financial and other competing interests for principal investigators for the overall trial and each study site

Example

"*PS*:

1. Was the Principal Investigator of the second International Stroke Trial (IST-2) to evaluate a neuroprotective compound (619c89). . .
2. Has received lecture fees and travel expenses from Bayer and from Boehringer Ingelheim for lectures given at international conferences.
3. He serves on the Independent Data Monitoring and Safety Board of the RELY trial, funded by Boehringer Ingelheim and receives attendance fees and travel expenses for attending board meetings.
4. He does not have any paid consultancies with pharmaceutical companies, and is not a member of the Speaker's Panel of any company.

KBS:

Received an honorarium for a lecture from Boehringer Ingelheim and had costs for participating in scientific meetings reimbursed. . ." [124]

Explanation

Competing interests, or conflicts of interest, exist when there is potential for divergence between an individual's or institution's private interests and their responsibilities to scientific and publishing activities.[360] More positive outcomes, larger treatment effect sizes, and more favourable interpretation of results have been found in clinical trials with pharmaceutical industry sponsorship (Item 4)[27 36-38 42] and investigators who have declared competing interests,[57 60] compared to those without such interests. Although competing interests are most often associated with drug and device industries, they may exist with support from or affiliation with government agencies, charities, not for profit organisations, and professional and civic organisations.

Competing interests do not in themselves imply wrongdoing. Their disclosure and regular updating enables appropriate management plans to be developed and implemented, and facilitates transparent assessment of the potential for bias.

Many trials and non-industry sponsors have a conflict of interest policy for their investigators, and checklists are available to guide potential interests that should be disclosed and regularly updated by trial investigators.[361 362] Types of financial ties include salary support or grants; ownership of stock or options; honorariums (eg, for advice, authorship, or public speaking); paid consultancy or service on advisory boards and medical education companies; and receipt of patents or patents pending. Non-financial competing interests include academic commitments; personal or professional relationships; and political, religious, or other affiliations with special interests or advocacy positions.

Access to data

Item 29: Statement of who will have access to the final trial dataset, and disclosure of contractual agreements that limit such access for investigators

Explanation

The validity of results from interventional trials can be verified only by individuals who have full access to the complete final dataset. For some multicentre trials, only

Example

"12.10.1 Intra-Study Data Sharing
The Data Management Coordinating Center will oversee the intra-study data sharing process, with input from the Data Management Subcommittee.
All Principal Investigators (both US and host country) will be given access to the cleaned data sets. Project data sets will be housed on the Project Accept Web site and/or the file transfer protocol site created for the study, and all data sets will be password protected. Project Principal Investigators will have direct access to their own site's data sets, and will have access to other sites data by request. To ensure confidentiality, data dispersed to project team members will be blinded of any identifying participant information."[113]

the steering group has access to the full trial dataset in order to ensure that the overall results are not disclosed by an individual study site prior to the main publication. Many of these trials will allow site investigators to access the full dataset if a formal request describing their plans is approved by the steering group. The World Medical Association supports the principle that trial investigators retain the right to access data.[363] However, among protocols of industry initiated randomised trials published in 2008-9 in the *Lancet* or approved in 2004 by a Danish ethics committee, 30-39% stated that the sponsor owned the data while 0-3% stated that principal investigators had access to all trial data.[10 364] Similar constraints were found in Danish trial protocols from 1994-5.[10]

The protocol should identify the individuals involved in the trial who will have access to the full dataset. Any restrictions in access for trial investigators should also be explicitly described.

Ancillary and post-trial care

Item 30: Provisions, if any, for ancillary and post-trial care, and for compensation to those who suffer harm from trial participation

Explanation

The provision of ancillary care refers to the provision of care beyond that immediately required for the proper and safe conduct of the trial, and the treatment of immediate adverse events related to trial procedures. It is generally agreed that trial sponsors and investigators should plan to provide care for participants' healthcare needs that arise as a direct consequence of trial participation (eg, intervention related harms). It is also important to consider whether care should be provided for certain ancillary needs that may otherwise arise during trial participation. Provision of care for ancillary needs reflects the fact that participants implicitly, but unavoidably, entrust certain aspects of their health to the research team. The scope of entrustment will vary depending on the nature of the trial (eg, setting, health condition under study, investigations performed).[366] Additional factors that influence the strength of the claim to ancillary care include participants' vulnerabilities; uncompensated burdens and harms; the intensity and duration of the participant-researcher relationship; and the degree to which participants are uniquely dependent on the research team for health care.[367]

The Declaration of Helsinki states that "the protocol should describe arrangements for post-study access by

Examples

"Patients that are enrolled into the study are covered by indemnity for negligent harm through the standard NHS [National Health Service] Indemnity arrangements. The University of Sheffield has insurance to cover for non-negligent harm associated with the protocol . . . This will include cover for additional health care, compensation or damages whether awarded voluntarily by the Sponsor, or by claims pursued through the courts. Incidences judged to arise from negligence (including those due to major protocol violations) will not be covered by study insurance policies. The liability of the manufacturer of IL1RA (Amgen Corporation) is strictly limited to those claims arising from faulty manufacturing of the commercial product and not to any aspects of the conduct of the study."[145]

"13.6 Access to Effective Products
Should this study provide evidence of the effectiveness of TDF [tenofovir disoproxil fumarate], FTC [emtricitabine]/TDF and/or tenofovir 1% gel in preventing HIV infection, it will be critical to provide access to the effective product(s) to study participants, their communities, and the worldwide population at risk for HIV infection in a timely manner. In preparation for this study, discussions have begun with Gilead Sciences, Inc. and CONRAD [Contraceptive Research and Development Organization] to ensure such access. Considerations under discussion include licensing agreements and preferred pricing arrangements for the study communities and other resource-poor settings.
While this study is ongoing, the MTN [Microbicide Trials Network] will continue these discussions. In addition, discussions will be initiated with other public and private funding sources such as the WHO, UNAIDS, Gates Foundation, and appropriate site government agencies that may be able to purchase product supplies in bulk and offer them at low or no cost to the study communities and other resource-poor communities most in need of the product(s). Operations and marketing research also may be conducted to determine how best to package and distribute the products, and maximize their acceptability and use, in at-risk populations."[365]

study participants to interventions identified as beneficial in the study or access to other appropriate care or benefits."[1] This principle is particularly applicable—and controversial—when research enabling the development and regulatory approval of interventions is performed in countries where subsequent access to the interventions is limited by cost or lack of availability.[368]

The protocol should describe any plans to provide or pay for ancillary care during the trial and identify any interventions, benefits, or other care that the sponsor will continue to provide to participants and host communities after the trial is completed.[369] Any plans to compensate participants for trial related harms should also be outlined.

Dissemination policy—trial results

Item 31a: Plans for investigators and sponsor to communicate trial results to participants, healthcare professionals, the public, and other relevant groups (eg, via publication, reporting in results databases, or other data sharing arrangements), including any publication restrictions

Explanation

A fundamental ethical principle in clinical trials is that the potential risks incurred by study participants should be balanced by the benefit of contributing to publicly available knowledge.[371] Unfortunately, about half of

chaa006386.indd 31 31/01/2013 10:33:39

Example

"XII. Publication Policy

The Publications subcommittee will review all publications following the guidelines given below and report its recommendations to the Steering Committee.

A. Data analysis and release of results

The scientific integrity of the project requires that the data from all BEST [Beta-Blocker Evaluation of Survival Trial] sites be analyzed study-wide and reported as such. Thus, an individual center is not expected to report the data collected from its center alone . . . all presentations and publications are expected to protect the integrity of the major objective(s) of the study; data that break the blind will not be presented prior to the release of mainline results. Recommendations as to the timing of presentation of such endpoint data and the meetings at which they might be presented will be given by the Steering Committee.

B. Review process

Each paper or abstract, as described below, must be submitted to the appropriate Subcommittee for review of its appropriateness and scientific merit prior to submission. The Subcommittee may recommend changes to the authors and will finally submit its recommendations to the Steering Committee for approval.

C. Primary outcome papers

The primary outcome papers of BEST are papers that present outcome data . . . The determination of whether or not a particular analysis represents a primary outcome will be made by the Steering Committee on the recommendation of the Publications Subcommittee . . .

D. Other study papers, abstracts and presentations

All studies other than those designated as "Primary Outcome" fall within this category . . . All papers and abstracts must be approved by the Publications Committee before they are submitted.

It is possible that in certain instances BEST may be asked to contribute papers to workshops, symposia, volumes, etc. The individuals to work on such requests should be appointed by the Executive Committee, but where time permits, a proposal will be circulated soliciting other participants as in the case of other study papers as described in the Application Review Process.

XIII. Close-out Procedures

BEST may terminate at the planned target of 1.5 years after the last participant has been randomized, or at an earlier or later date if the circumstances warrant . . . Regardless of the timing and circumstances of the end of the study, close-out will proceed in two stages:

- Interim period for analysis and documentation of study results.
- Debriefing of participants and dissemination of study results.

A. Interim

Every attempt will be made to reduce to an absolute minimum the interval between the completion of data collection and the release of the study results. We expect to take about 3 to 4 months to compile the final results paper for an appropriate journal.

B. Reporting of study results

The study results will be released to the participating physicians, referring physicians, patients and the general medical community."[370]

clinical trials remain unpublished.[80 83] Trials with statistically non-significant results or industry funding are more prone to non-publication,[36 38 80-83] although government funded trials are also susceptible.[81] When published, trials with non-significant results often have a longer delay to publication.[80 83] Overall, the medical literature represents a biased subset of existing data, potentially leading to overestimation of benefits, underestimation of harms, and a detrimental impact on patient care and research.[80 372-377]

Although peer reviewers can be biased in favour of positive findings,[378] lack of publication appears to be primarily due to trial investigators or sponsors failing to submit negative or null results, rather than journals rejecting them.[80 379] A plan to disseminate trial results to key stakeholders should be outlined in the protocol, including a process and timeframe for approving and submitting reports for dissemination (eg, via journal publication, trial registry, trial website), and an explicit statement that the results will be disseminated regardless of the magnitude or direction of effect.

Furthermore, any conditions relating to the investigators' right to publish or present trial results should be explicitly described. Publication restrictions have been imposed by various groups, including industry sponsors or the trial steering group (eg, to maintain the integrity of the overall dataset).[10 380] These restrictions are sometimes not described in the protocol but rather in separate publication agreements.[10] However, as they can interfere with the ethical responsibility of investigators and sponsors to disseminate trial results in an unbiased and timely manner,[38 381-384] any restrictions should be disclosed in the protocol for review by REC/IRBs, funders, and other stakeholders. A review of industry initiated randomised trial protocols approved in Denmark in 1994-95 revealed that 91% had publication restrictions imposed by sponsors; similar constraints were noted for protocols approved in 2004.[10]

Dissemination policy—authorship

Item 31b: Authorship eligibility guidelines and any intended use of professional writers

Example

"17.4. Assignment of Writing Committees

Topics suggested for presentation or publication will be circulated to the PIs [principal investigators] of the CCCs [core coordinating centers], the DCC [data coordinating centre], Core Lab and the NIH [National Institutes of Health]. These groups are requested to suggest and justify names for authors to be reviewed by the PC [publications committee]. . . If a topic is suggested by a participant of the FSGS-CT [focal segmental glomerulosclerosis—clinical trial], the writing committee will be formed as just described except that the person making the suggestion may be considered as the lead author. The PI of an ancillary study should be considered for lead author of material derived from this study. Disputes regarding authorship will be settled by the Study Chair after consultation with the Chair of the PC . . .

17.5. Reports of the FSGS-CT: Classes of Reports

There are three classes of reports of the FSGS-CT:

A. Reports of the major outcomes of the Study.

B. Reports addressing in detail one aspect of the FSGS-CT, but in which the data are derived from the entire study.

C. Reports of data derived from a subset of centers by members of the FSGS-CT, (eg, sub-studies or ancillary studies), or reports of investigations initiated outside of the FSGS-CT, but using data or samples collected by the FSGS-CT. . .

17.6. Authorship Policy

The authors of FSGS publications will be listed as detailed below.

Type A publications:

abstracts: from the FSGS Clinical Trial Group[x], presented by XXXX.

papers: from the FSGS Clinical Trial Group[x], prepared by XXXX.

[x]The FSGS participant box, detailed below, must be included in these papers. If a journal's publication policy does not allow authorship by a group, the authors will be listed first as in Type B publications.

Type B publications:

. . .

17.7. Authorship: Professional Participants Listing in the FSGS Participant Box

The FSGS participant box will list all professionals that have participated in the FSGS-CT for a minimum of one year."[267]

chaa006386.indd 32 31/01/2013 10:33:39

Explanation

Substantive contributions to the design, conduct, interpretation, and reporting of a clinical trial are recognised through the granting of authorship on the final trial report. Authorship guidelines in the protocol are intended to help enhance transparency and avoid disputes or misunderstanding after trial completion. These guidelines should define criteria for individually named authors or group authorship.[385]

Individuals who fulfil authorship criteria should not remain hidden (ghost authorship) and should have final authority over manuscript content.[9 386 387] Similarly, those who do not fulfil such criteria should not be granted authorship (guest authorship).[386 388] The International Committee of Medical Journal Editors has defined authorship criteria for manuscripts submitted for publication,[389] although these criteria have reportedly been open to abuse.[390] If some protocol authors are not named authors of subsequent publications, their role in protocol design should at least be acknowledged in the published report. Among 44 protocols of industry initiated trials, 75% had evidence of ghost authorship when compared with corresponding journal publications.[9]

Professional medical writers are sometimes hired to improve clarity and structure in a trial report, and guidelines for ethical collaborative writing have been developed.[391 392] Because the drafting of text can influence how the study results and conclusions are portrayed, plans for the employment of writers and their funding source should be acknowledged in both protocols and trial reports.

Dissemination policy—reproducible research

Item 31c: Plans, if any, for granting public access to the full protocol, participant-level dataset, and statistical code

> **Example**
>
> "**Data sharing statement** No later than 3 years after the collection of the 1-year postrandomisation interviews, we will deliver a completely deidentified data set to an appropriate data archive for sharing purposes."[393]

Explanation

Given the central role of protocols in enhancing transparency, reproducibility, and interpretation of trial results, there is a strong ethical and scientific imperative to ensure that full protocols are made publicly available.[24 394 395] High quality protocols contain relevant details on study design and conduct that are generally not available in journal publications or trial registries.[84 396] It is also important to make available the full study report, such as the "clinical study report" submitted to regulatory agencies by industry sponsors.[377 396-400] This detailed report provides the most comprehensive description of trial methods (including the full protocol) and all published and unpublished analyses. In addition, there have increasingly been calls to improve the availability of participant-level datasets and statistical code after journal publication to enable verification and replication of analyses, facilitate pooling with other studies, and accelerate research through open knowledge sharing.[372 401-406]

Avenues for providing access to full protocols include journals,[407 408] trial websites, and trial registries.[163] Several journals and funders support the sharing of participant level data,[405 409-411] while others routinely publish a statement regarding sharing of protocols, statistical codes, and datasets for all of their published research articles.[412 413] The protocol should indicate whether the trial protocol, full study report, anonymised participant level dataset, and statistical code for generating the results will be made publicly available; and if so, describe the timeframe and any other conditions for access.

Section 5: Appendices

Informed consent materials

Item 32: Model consent form and other related documentation given to participants and authorised surrogates

> **Example**
>
> "APPENDIX 7 SAMPLE PATIENT INFORMED CONSENT
>
> Note: . . . Each Ethics Committee or Institutional Review Board will revise and adapt according to their own institution's guidelines.
>
> MULTICENTER PHASE III RANDOMIZED TRIAL COMPARING DOXORUBICIN AND CYCLOPHOSPHAMIDE . . .
>
> Study number: BCIRG 006 (TAX GMA 302)
>
> Investigator name:
>
> Address:
>
> Consent Form:
>
> This consent form is part of the informed consent process. It is designed to give you an idea of what this research study is about and what will happen to you if you choose to be in the study. . ."[345]

Explanation

The Declaration of Helsinki states that each potential trial participant must normally, at a minimum, be adequately informed about the purpose of the trial; potential benefits and risks; their right to refuse participation or to withdraw consent at any time; institutional affiliation and potential competing interests of the researcher; and sources of trial funding.[1] There are rare exceptions where deferred consent can be acceptable, such as trials involving unconscious patients in emergency situations.

Special attention is required to ensure that relevant information is provided and appropriate modes of delivery are used during the consent process (Item 26).[414] Consent and participant information forms are often written at a much higher reading level than is acceptable for the general population.[415] Depending on the nature of the trial, several different consent documents may be needed. For example, a paediatric trial may involve both parental permission and participant assent documents. For multicentre trials, a model or sample document is typically drafted for distribution to local investigators, who may then revise the document to comply with local requirements.

Biological specimens

Item 33: Plans for collection, laboratory evaluation, and storage of biological specimens for genetic or molecular analysis in the current trial and for future use in ancillary studies, if applicable

chaa006386.indd 33 31/01/2013 10:33:39

Example

"White Blood Cell and Plasma Collection Procedures

1.0 Objectives

1.1 To provide a resource for studies of early markers, etiology, and genetic risk factors for prostate cancer and other diseases.

2.0 Background

The Prostate Cancer Prevention Trial (PCPT) is a randomized double blind chemoprevention trial . . . Initial blood collection was specifically for the analysis of PSA [prostate specific antigen] and storage of serum . . . an additional blood collection will be carried out using anticoagulant so that plasma and white blood cells can be isolated. Plasma will allow the analysis of additional biomarkers . . . This DNA will be used (among other possible uses) for studies to investigate polymorphisms in genes which may influence prostate cancer risk . . .

The PCPT WBC [white blood cell] sample will be available to PCPT investigators as well as outside researchers who have important, timely hypotheses to test. Because the sample bank is a limited resource, proposals to use it will be evaluated in terms of scientific relevance, significance, and validity as well as the potential impact of the proposed study. The amount and type of material needed will also be considered and the efficient use of material will be required. Strict confidentiality will be exercised and the information provided to investigators will not contain personal identifiers. When specific uses of the WBC samples are approved, the SWOG-9217 protocol will be amended. Participation in this research is not required for continued participation in the PCPT.

3.0 Methods

3.1 Because the original model consent form did not specifically address genetic studies, participants will be asked to sign an additional consent form to document their consent to the collection and submission of additional blood samples for storage and future testing (including genetic analysis).

3.2 Institutions will be asked to submit additional materials from participants who consent to the additional blood collection. The blood is to be collected, processed and shipped as described in the PCPT Study Manual.

3.3 NCI-Frederick Cancer Research Development Center (FCRDC) in Frederick, Maryland will serve as the processing, aliquotting and storage facility.

3.4 Upon arrival at FCRDC the blood will be pooled and centrifuged. Plasma will be separated into 5 x 1.8 ml aliquots and frozen . . .

3.5 All samples will be logged in and aliquots will be bar coded with a unique storage ID. These data will be electronically transmitted to the Statistical Center for verification.

3.6 The scientists who will carry out analyses on these materials will not have access to personal identifiers and will not be able to link the results of these tests to personal identifier information. No individual results will be presented in publications or other reports. . . .

3.7 Participants will not be informed on an individual basis of any results from these studies . . .

4.0 Sample analysis

4.1 Investigators planning to submit NIH [National Institutes of Health] grant applications must obtain approval for their study and specimen access from the PCPT Serum and Tissue Utilization Committee before submission of a grant proposal. Potential investigators will be required to submit a brief abstract and 1-4 page outline . . . This proposal will be circulated for review to members of the PCPT Serum and Tissue Utilization Committee and two ad hoc members having relevant expertise . . .

4.2 It is anticipated that proposals will be reviewed once a year . . . Approval by this group as well as appropriate Institutional Review Board approval from the investigator's institution will be required before release of samples."[416]

Explanation

Biological specimens (eg, biopsy tissue; blood for DNA extraction) obtained during the conduct of clinical trials can be stored in repositories—often designated as biobanks—for the current trial and future research. This process is usually governed by local regulation and has particular ethical considerations (Item 26b).

If the trial involves genetic or molecular analysis of biological specimens derived from humans, or if any specimens will be stored for future use (specified or unspecified), the protocol should describe details about specimen collection, storage, and evaluation, including the location of repositories. In addition, the protocol should state whether collected samples and associated participant related data will be de-identified or coded to protect participant confidentiality. If a repository is overseen by a named research ethics committee/institutional review board, then this information should also be provided.

Discussion

It is critical that every clinical trial has a complete and transparent protocol, which can then facilitate trial conduct and appraisal by communicating relevant information to key stakeholders. In response to observed deficiencies in protocol content, the SPIRIT Initiative has produced recommendations for minimum relevant protocol items to include in a protocol, published in the form of the SPIRIT 2013 Statement and this Explanation and Elaboration (E&E) paper.[14] The strengths that distinguish SPIRIT from other protocol guidance documents include its systematic and transparent development methods; participation of a wide range of key stakeholders; use of empirical evidence to support its recommendations; and availability of detailed guidance including model examples from protocols.

The overall aim of SPIRIT is to improve the completeness and transparency of trial protocols. The SPIRIT documents can serve as a practical resource for trial investigators and personnel to draft and understand the key elements of a protocol. In doing so, our vision is that the SPIRIT 2013 Statement and E&E paper will also facilitate and expedite the review of protocols by research ethics committees/institutional review boards, scientific review groups, and funders—for example, by reducing the number of avoidable queries to trial investigators regarding missing or unclear protocol information during the review process. Furthermore, improved protocol content would help facilitate the critical appraisal of final trial reports and results. Finally, several SPIRIT items correspond to items on the CONSORT 2010 checklist (Consolidated Standards of Reporting Trials),[417] which should facilitate the transition from the protocol to the final study report.

The next steps for the SPIRIT Initiative include an implementation strategy to encourage uptake of the SPIRIT 2013 Statement. The SPIRIT website (www.spirit-statement.org) will provide the latest resources and information on the initiative, including a list of supporters. We invite stakeholders to assist in the evaluation of the SPIRIT Statement and E&E paper by using the documents and providing feedback to inform future revisions. Through widespread uptake and support, the potential to improve the completeness and quality of trial protocols, as well as the efficiency of their review, can be fully realised.

We thank Raymond Daniel for his help with reference management and Jessica Kitchen for her work with manuscript formatting and identification of protocol examples. We also acknowledge GlaxoSmithKline for providing a sample of their trial protocols to serve as potential examples.

Competing interests: All authors have completed the ICJME unified declaration form at www.icmje.org/coi_disclosure.pdf (available on request from the corresponding author) and declare: JAB is employed by the Janssen Pharmaceutical Companies of Johnson & Johnson; KKJ was formerly employed by CIHR (Knowledge Translation Branch), and WRP is affiliated with the NCIC Clinical Trials Group. Trish Groves is deputy editor of *BMJ* and a member of the SPIRIT group but did not take part in the peer review and decision making process about this publication.

Contributors: AWC, JT, and DM conceived of the paper. All authors contributed to the drafting and revision of the manuscript, and approve the final version. AWC is the guarantor for the article.

Funding: The SPIRIT meetings were funded by the Canadian Institutes of Health Research (CIHR grant DET - 106068); National Cancer Institute of Canada (now Canadian Cancer Society Research Institute); and Canadian Agency for Drugs and Technologies in Health. CIHR has also funded ongoing dissemination activities (grant MET-117434). KKJ was formerly employed by CIHR (Knowledge Translation Branch), and WRP is affiliated with the NCIC Clinical Trials Group. The funders had no input into the

design and conduct of the project; collection, management, analysis, and interpretation of the data; and preparation, review, or approval of the manuscript.

Provenance and peer review: Not commissioned; externally peer reviewed.

1 World Medical Association. WMA Declaration of Helsinki—ethical principles for medical research involving human subjects. 2008. www.wma.net/en/30publications/10policies/b3/index.html.
2 Pildal J, Chan A-W, Hróbjartsson A, Forfang E, Altman DG, Gøtzsche PC. Comparison of descriptions of allocation concealment in trial protocols and the published reports: cohort study. *BMJ* 2005;330:1049.
3 Hróbjartsson A, Pildal J, Chan A-W, Haahr MT, Altman DG, Gøtzsche PC. Reporting on blinding in trial protocols and corresponding publications was often inadequate but rarely contradictory. *J Clin Epidemiol* 2009;62:967-73.
4 Chan A-W, Hróbjartsson A, Haahr MT, Gøtzsche PC, Altman DG. Empirical evidence for selective reporting of outcomes in randomized trials: comparison of protocols to published articles. *JAMA* 2004;291:2457-65.
5 Scharf O, Colevas AD. Adverse event reporting in publications compared with sponsor database for cancer clinical trials. *J Clin Oncol* 2006;24:3933-8.
6 Chan A-W, Hróbjartsson A, Jørgensen KJ, Gøtzsche PC, Altman DG. Discrepancies in sample size calculations and data analyses reported in randomised trials: comparison of publications with protocols. *BMJ* 2008;337:a2299.
7 Al-Marzouki S, Roberts I, Evans S, Marshall T. Selective reporting in clinical trials: analysis of trial protocols accepted by the Lancet. *Lancet* 2008;372:201.
8 Hernández AV, Steyerberg EW, Taylor GS, Marmarou A, Habbema JD, Maas AI. Subgroup analysis and covariate adjustment in randomized clinical trials of traumatic brain injury: a systematic review. *Neurosurgery* 2005;57:1244-53.
9 Gøtzsche PC, Hróbjartsson A, Johansen HK, Haahr MT, Altman DG, Chan A-W. Ghost authorship in industry-initiated randomised trials. *PLoS Med* 2007;4:e19.
10 Gøtzsche PC, Hróbjartsson A, Johansen HK, Haahr MT, Altman DG, Chan A-W. Constraints on publication rights in industry-initiated clinical trials. *JAMA* 2006;295:1645-6.
11 Mhaskar R, Djulbegovic B, Magazin A, Soares HP, Kumar A. Published methodological quality of randomized controlled trials does not reflect the actual quality assessed in protocols. *J Clin Epidemiol* 2012;65:602-9.
12 Smyth RM, Kirkham JJ, Jacoby A, Altman DG, Gamble C, Williamson PR. Frequency and reasons for outcome reporting bias in clinical trials: interviews with trialists. *BMJ* 2011;342:c7153.
13 Tetzlaff JM, Chan A-W, Kitchen J, Sampson M, Tricco AC, Moher D. Guidelines for randomized controlled trial protocol content: a systematic review. *Syst Rev* 2012;1:43.
14 Chan A-W, Tetzlaff JM, Altman DG, Laupacis A, Gøtzsche PC, Krleža-Jerić K, et al. SPIRIT 2013 Statement: Defining standard protocol items for clinical trials. *Ann Intern Med* 2013. www.annals.org/article.aspx?doi=10.7326/0003-4819-158-3-201302050-00583.
15 Tetzlaff JM, Moher D, Chan A-W. Developing a guideline for reporting clinical trial protocols: Delphi consensus survey. *Trials* 2012;13:176.
16 Moher D, Schulz KF, Simera I, Altman DG. Guidance for developers of health research reporting guidelines. *PLoS Med* 2010;7:e1000217.
17 Moher D, Hopewell S, Schulz KF, Montori V, Gøtzsche PC, Devereaux PJ, et al. CONSORT 2010 Explanation and Elaboration: updated guidelines for reporting parallel group randomised trials. *BMJ* 2010;340:c869.
18 Liberati A, Altman DG, Tetzlaff J, Mulrow C, Gøtzsche PC, Ioannidis JP, et al. The PRISMA statement for reporting systematic reviews and meta-analyses of studies that evaluate health care interventions: explanation and elaboration. *J Clin Epidemiol* 2009;62:e1-34.
19 Warner Chilcott. A comparison of once a day dose compared to 2 doses/day. http://clinicaltrials.gov/show/NCT00505778.
20 Dickersin K, Manheimer E, Wieland S, Robinson KA, Lefebvre C, McDonald S. Development of the Cochrane Collaboration's CENTRAL Register of controlled clinical trials. *Eval Health Prof* 2002;25:38-64.
21 Shaw L, Price C, McLure S, Howel D, McColl E, Ford GA. Paramedic Initiated Lisinopril For Acute Stroke Treatment (PIL-FAST): study protocol for a pilot randomised controlled trial [protocol]. *Trials* 2011;12:152.
22 Sim I, Chan A-W, Gülmezoglu AM, Evans T, Pang T. Clinical trial registration: transparency is the watchword. *Lancet* 2006;367:1631-3.
23 Dickersin K, Rennie D. Registering clinical trials. *JAMA* 2003;290:516-23.
24 Krleža-Jerić K, Chan A-W, Dickersin K, Sim I, Grimshaw J, Gluud C for the Ottawa Group. Principles for international registration of protocol information and results from human trials of health related interventions: Ottawa statement (part 1). *BMJ* 2005;330:956-8.
25 DeAngelis CD, Drazen JM, Frizelle FA, Haug C, Hoey J, Horton R, et al. Clinical trial registration: a statement from the International Committee of Medical Journal Editors. *JAMA* 2004;292:1363-4.
26 Mathieu S, Boutron I, Moher D, Altman DG, Ravaud P. Comparison of registered and published primary outcomes in randomized controlled trials. *JAMA* 2009;302:977-84.
27 Bourgeois FT, Murthy S, Mandl KD. Outcome reporting among drug trials registered in ClinicalTrials.gov. *Ann Intern Med* 2010;153:158-66.
28 You B, Gan HK, Pond G, Chen EX. Consistency in the analysis and reporting of primary end points in oncology randomized controlled trials from registration to publication: a systematic review. *J Clin Oncol* 2012;30:210-6.
29 United States Congress. Food and Drug Administration Amendments Act of 2007, Title VIII, Section 801. Expanded clinical trial registry data bank. 2007. www.govtrack.us/congress/billtext.xpd?bill=h110-3580.
30 European Commission. Communication from the Commission regarding the guideline on the data fields contained in the clinical trials database provided for in Article 11 of Directive 2001/20/EC to be included in the database on medicinal products provided for in Article 57 of Regulation (EC) No 726/2004 (2008/C 168/02). *Official Journal of the European Union* 2008;51:3-4.
31 Laine C, Horton R, DeAngelis CD, Drazen JM, Frizelle FA, Godlee F, et al. Clinical trial registration. *BMJ* 2007;334:1177-8.
32 Bernhard Nocht Institute for Tropical Medicine. Probiotic Saccharomyces boulardii for the prevention of antibiotic-associated diarrhoea (SacBo). http://clinicaltrials.gov/ct2/show/NCT01143272.
33 Dalessandro M, Hirman J. Protocol SB-275833/030—Studies 030A and 030B: two identical double-blind, double-dummy, multicenter, comparative phase III studies of the safety and efficacy of topical 1% SB-275833, applied twice daily, versus oral Cephalexin, 500 mg in adults, or 12.5 mg/kg (250 mg/5 ml) in children, twice daily, in the treatment of uncomplicated secondarily infected traumatic lesions [protocol]. Version 5 (July 25, 2005). www.spirit-statement.org/wp-content/uploads/2012/12/Protocol-SB-275833.pdf.
34 Effect of tranexamic acid on coagulation in a sample of participants in the WOMAN trial: WOMAN-ETAC study [protocol]. Version 1 (August 3, 2011). www.thewomantrial.lshtm.ac.uk/Images/WOMAN_ETACprotocol.pdf.
35 Chan A-W, Krleža-Jerić K, Schmid I, Altman DG. Outcome reporting bias in randomized trials funded by the Canadian Institutes of Health Research. *CMAJ* 2004;171:735-40.
36 Lexchin J, Bero LA, Djulbegovic B, Clark O. Pharmaceutical industry sponsorship and research outcome and quality: systematic review. *BMJ* 2003;326:1167-70.
37 Als-Nielsen B, Chen W, Gluud C, Kjaergard LL. Association of funding and conclusions in randomized drug trials: a reflection of treatment effect or adverse events? *JAMA* 2003;290:921-8.
38 Bekelman JE, Li Y, Gross CP. Scope and impact of financial conflicts of interest in biomedical research: a systematic review. *JAMA* 2003;289:454-65.
39 Heres S, Davis J, Maino K, Jetzinger E, Kissling W, Leucht S. Why olanzapine beats risperidone, risperidone beats quetiapine, and quetiapine beats olanzapine: an exploratory analysis of head-to-head comparison studies of second-generation antipsychotics. *Am J Psychiatry* 2006;163:185-94.
40 Djulbegovic B, Cantor A, Clarke M. The importance of preservation of the ethical principle of equipoise in the design of clinical trials: relative impact of the methodological quality domains on the treatment effect in randomized controlled trials. *Account Res* 2003;10:301-15.
41 Etter J-F, Burri M, Stapleton J. The impact of pharmaceutical company funding on results of randomized trials of nicotine replacement therapy for smoking cessation: a meta-analysis. *Addiction* 2007;102:815-22.
42 Golder S, Loke YK. Is there evidence for biased reporting of published adverse effects data in pharmaceutical industry-funded studies? *Br J Clin Pharmacol* 2008;66:767-73.
43 Min Y-I, Unalp-Arida A, Scherer R, Dickersin K. Assessment of equipoise using a cohort of randomized controlled trials [abstract]. International congress on peer review and biomedical publication, Chicago, IL, 16-18 September, 2005.
44 Yaphe J, Edman R, Knishkowy B, Herman J. The association between funding by commercial interests and study outcome in randomized controlled drug trials. *Fam Pract* 2001;18:565-8.
45 Ahmer S, Arya P, Anderson D, Faruqui R. Conflict of interest in psychiatry. *Psychiatr Bull* 2005;29:302-4.
46 The Danish National Committee on Biomedical Research Ethics. Guidelines about notification etc. of a biomedical research project to the committee system on biomedical research ethics, No 9154, 5 May 2011. 2011. www.cvk.sum.dk/English/guidelinesaboutnotification.aspx.
47 Lester RT, Mills EJ, Kariri A, Ritvo P, Chung M, Jack W, et al. The HAART cell phone adherence trial (WelTel Kenya1): a randomized controlled trial protocol [protocol]. *Trials* 2009;10:87.
48 Rennie D, Yank V, Emanuel L. When authorship fails. A proposal to make contributors accountable. *JAMA* 1997;278:579-85.
49 Trials. Instructions for authors— study protocols. 2012. www.trialsjournal.com/authors/instructions/studyprotocol#formatting-contributions.
50 Williams H. Bullous Pemphigoid Steroids and Tetracyclines (BLISTER) Study. A randomised controlled trial to compare the safety and effectiveness of doxycycline (200 mg/day) with prednisolone (0.5 mg/kg/day) for initial treatment of bullous pemphigoid [protocol]. Version 4.0 (July 20, 2011). www.spirit-statement.org/wp-content/uploads/2012/12/Blister-Protocol-v4-20July2011.pdf .

51 Gertel A, Block P, Gawrylewski H-M, Raymond S, Quinn T, Muhlbradt E. CDISC Clinical research glossary. Version 8.0. 2009. www.cdisc.org/stuff/contentmgr/files/0/be650811feb46f381f0af41ca40ade2e/misc/cdisc_2009_glossary.pdf.
52 World Health Organization. Operational guidelines for ethics committees that review biomedical research. 2000. www.who.int/tdr/publications/documents/ethics.pdf.
53 World Health Organization. Handbook for good clinical research practice (GCP): Guidance for implementation. 2002. http://apps.who.int/prequal/info_general/documents/GCP/gcp1.pdf.
54 Pierce MA, Hess EP, Kline JA, Shah ND, Breslin M, Branda ME, et al. The Chest Pain Choice trial: a pilot randomized trial of a decision aid for patients with chest pain in the emergency department [protocol]. *Trials* 2010;11:57.
55 Vlad SC, LaValley MP, McAlindon TE, Felson DT. Glucosamine for pain in osteoarthritis: why do trial results differ? *Arthritis Rheum* 2007;56:2267-77.
56 Kjaergard LL, Als-Nielsen B. Association between competing interests and authors' conclusions: epidemiological study of randomised clinical trials published in the BMJ. *BMJ* 2002;325:249.
57 Liss H. Publication bias in the pulmonary/allergy literature: effect of pharmaceutical company sponsorship. *Isr Med Assoc J* 2006;8:451-4.
58 Montgomery JH, Byerly M, Carmody T, Li B, Miller DR, Varghese F, et al. An analysis of the effect of funding source in randomized clinical trials of second generation antipsychotics for the treatment of schizophrenia. *Control Clin Trials* 2004;25:598-612.
59 Perlis RH, Perlis CS, Wu Y, Hwang C, Joseph M, Nierenberg AA. Industry sponsorship and financial conflict of interest in the reporting of clinical trials in psychiatry. *Am J Psychiatry* 2005;162:1957-60.
60 Jagsi R, Sheets N, Jankovic A, Motomura AR, Amarnath S, Ubel PA, et al. Frequency, nature, effects, and correlates of conflicts of interest in published clinical cancer research. *Cancer* 2009;115:2783-91.
61 Mello MM, Clarridge BR, Studdert DM. Academic medical centers' standards for clinical-trial agreements with industry. *N Engl J Med* 2005;352:2202-10.
62 European Vasculitis Study Group (EUVAS). RITUXVAS Clinical Trial Protocol: An international, randomised, open label trial comparing a rituximab based regimen with a standard cyclophosphamide/azathioprine regimen in the treatment of active, 'generalised' ANCA associated vasculitis [protocol]. Version 1b (November 15, 2005). www.vasculitis.nl/media/documents/rituxvas.pdf.
63 Delgado-Rodriguez M, Ruiz-Canela M, De Irala-Estevez J, Llorca J, Martinez-Gonzalez MA. Participation of epidemiologists and/or biostatisticians and methodological quality of published controlled clinical trials. *J Epidemiol Community Health* 2001;55:569-72.
64 Llorca J, Martinez-Sanz F, Prieto-Salceda D, Fariñas-Alvarez C, Chinchon MV, Quinones D, et al. Quality of controlled clinical trials on glaucoma and intraocular high pressure. *J Glaucoma* 2005;14:190-5.
65 CRASH2 Clinical Randomisation of an Antifibrinolytic in Significant Haemorrhage. A large randomised placebo controlled trial among trauma patients with or at risk of significant haemorrhage, of the effects of antifibrinolytic treatment on death and transfusion requirement [protocol]. Version 3 (July 2, 2005). www.crash2.lshtm.ac.uk/.
66 Clarke M. Doing new research? Don't forget the old. *PLoS Med* 2004;1:e35.
67 Prescott RJ, Counsell CE, Gillespie WJ, Grant AM, Russell IT, Kiauka S, et al. Factors that limit the quality, number and progress of randomised controlled trials. *Health Technol Assess* 1999;3:1-143.
68 Centre for Reviews and Dissemination. Systematic review of barriers, modifiers and benefits involved in participation in cancer trials. CRD Report 31. York: University of York, 2006.
69 Tournoux C, Katsahian S, Chevret S, Levy V. Factors influencing inclusion of patients with malignancies in clinical trials. *Cancer* 2006;106:258-70.
70 Clarke M, Hopewell S, Chalmers I. Clinical trials should begin and end with systematic reviews of relevant evidence: 12 years and waiting. *Lancet* 2010;376:20-1.
71 Canadian Institutes of Health Research. RCT evaluation criteria and headings. 2010. www.cihr.ca/e/39187.html.
72 National Institute for Health Research. Efficacy and mechanism evaluation program. Important information & guidance notes—preliminary application. 2012. www.eme.ac.uk/funding/Researcher-led.asp.
73 Jüni P, Nartey L, Reichenbach S, Sterchi R, Dieppe PA, Egger M. Risk of cardiovascular events and rofecoxib: cumulative meta-analysis. *Lancet* 2004;364:2021-9.
74 Puhan MA, Vollenweider D, Steurer J, Bossuyt PM, ter Riet G. Where is the supporting evidence for treating mild to moderate chronic obstructive pulmonary disease exacerbations with antibiotics? A systematic review. *BMC Med* 2008;6:28.
75 Fergusson D, Glass KC, Hutton B, Shapiro S. Randomized controlled trials of aprotinin in cardiac surgery: could clinical equipoise have stopped the bleeding? *Clin Trials* 2005;2:218-29.
76 Lau J, Antman EM, Jimenez-Silva J, Kupelnick B, Mosteller F, Chalmers TC. Cumulative meta-analysis of therapeutic trials for myocardial infarction. *N Engl J Med* 1992;327:248-54.
77 Robinson KA, Goodman SN. A systematic examination of the citation of prior research in reports of randomized, controlled trials. *Ann Intern Med* 2011;154:50-5.
78 Goudie AC, Sutton AJ, Jones DR, Donald A. Empirical assessment suggests that existing evidence could be used more fully in designing randomized controlled trials. *J Clin Epidemiol* 2010;63:983-91.
79 Cooper NJ, Jones DR, Sutton AJ. The use of systematic reviews when designing studies. *Clin Trials* 2005;2:260-4.
80 Song F, Parekh S, Hooper L, Loke YK, Ryder J, Sutton AJ, et al. Dissemination and publication of research findings: an updated review of related biases. *Health Technol Assess* 2010;14:iii-193.
81 Ross JS, Tse T, Zarin DA, Xu H, Zhou L, Krumholz HM. Publication of NIH funded trials registered in ClinicalTrials.gov: cross sectional analysis. *BMJ* 2012;344:d7292.
82 Ross JS, Mulvey GK, Hines EM, Nissen SE, Krumholz HM. Trial publication after registration in ClinicalTrials.Gov: a cross-sectional analysis. *PLoS Med* 2009;6:e1000144.
83 Hopewell S, Loudon K, Clarke MJ, Oxman AD, Dickersin K. Publication bias in clinical trials due to statistical significance or direction of trial results. *Cochrane Database Syst Rev* 2009;1:MR000006.
84 Chan A-W. Out of sight but not out of mind: how to search for unpublished clinical trial evidence. *BMJ* 2012;344:d8013.
85 A phase III multi-centre, randomised, double-blind, double-dummy, comparative clinical study to assess the safety and efficacy of a fixed-dose formulation of oral pyronaridine artesunate (180:60 mg tablet) versus chloroquine (155 mg tablet), in children and adult patients with acute Plasmodium vivax malaria [protocol]. Version 2.0 (March 5, 2007). www.plosone.org/article/info%3Adoi%2F10.1371%2Fjournal.pone.0014501#s5
86 Dawson L, Zarin DA, Emanuel EJ, Friedman LM, Chaudhari B, Goodman SN. Considering usual medical care in clinical trial design. *PLoS Med* 2009;6:e1000111.
87 Van Luijn JCF, Van Loenen AC, Gribnau FWJ, Leufkens HGM. Choice of comparator in active control trials of new drugs. *Ann Pharmacother* 2008;42:1605-12.
88 Johansen HK, Gøtzsche PC. Problems in the design and reporting of trials of antifungal agents encountered during meta-analysis. *JAMA* 1999;282:1752-9.
89 Stang A, Hense H-W, Jöckel K-H, Turner EH, Tramèr MR. Is it always unethical to use a placebo in a clinical trial? *PLoS Med* 2005;2:e72.
90 Emanuel EJ, Miller FG. The ethics of placebo-controlled trials—A middle ground. *N Engl J Med* 2001;345:915-9.
91 Ross S, Grant A, Counsell C, Gillespie W, Russell I, Prescott R. Barriers to participation in randomised controlled trials: a systematic review. *J Clin Epidemiol* 1999;52:1143-56.
92 Mills EJ, Seely D, Rachlis B, Griffith L, Wu P, Wilson K, et al. Barriers to participation in clinical trials of cancer: a meta-analysis and systematic review of patient-reported factors. *Lancet Oncol* 2006;7:141-8.
93 Rochon PA, Gurwitz JH, Simms RW. A study of manufacturer supported trials of non-steroidal anti-inflammatory drugs in the treatment of arthritis. *Arch Int Med* 1994;9:157-63.
94 Rutherford BR, Sneed JR, Roose SP. Does study design influence outcome? The effects of placebo control and treatment duration in antidepressant trials. *Psychother Psychosom* 2009;78:172-81.
95 Sneed JR, Rutherford BR, Rindskopf D, Lane DT, Sackeim HA, Roose SP. Design makes a difference: a meta-analysis of antidepressant response rates in placebo-controlled versus comparator trials in late-life depression. *Am J Geriatr Psychiatry* 2008;16:65-73.
96 Sinyor M, Levitt AJ, Cheung AH, Schaffer A, Kiss A, Dowlati Y, et al. Does inclusion of a placebo arm influence response to active antidepressant treatment in randomized controlled trials? Results from pooled and meta-analyses. *J Clin Psychiatry* 2010;71:270-9.
97 Tang J-L, Zhan S-Y, Ernst E. Review of randomised controlled trials of traditional Chinese medicine. *BMJ* 1999;319:160-1.
98 A phase 3, active (Warfarin) controlled, randomized, double-blind, parallel arm study to evaluate efficacy and safety of Apixaban in preventing stroke and systemic embolism in subjects with nonvalvular atrial fibrillation (ARISTOTLE: Apixaban for Reduction In STroke and Other ThromboemboLic Events in Atrial Fibrillation) [protocol]. Version 4 (August 4, 2010). www.nejm.org/doi/full/10.1056/NEJMoa1107039.
99 Fleming TR. Clinical trials: discerning hype from substance. *Ann Intern Med* 2010;153:400-406.
100 Heger U, Voss S, Knebel P, Doerr-Harim C, Neudecker J, Schuhmacher C, et al. Prevention of abdominal wound infection (PROUD trial, DRKS00000390): study protocol for a randomized controlled trial [protocol]. *Trials* 2011;12:245.
101 Hopewell S, Dutton S, Yu L-M, Chan A-W, Altman DG. The quality of reports of randomised trials in 2000 and 2006: comparative study of articles indexed in PubMed. *BMJ* 2010;340:c723.
102 Dumville JC, Hahn S, Miles JN, Torgerson DJ. The use of unequal randomisation ratios in clinical trials: a review. *Contemp Clin Trials* 2006;27:1-12.
103 Gilbody S, Bower P, Torgerson D, Richards D. Cluster randomized trials produced similar results to individually randomized trials in a meta-analysis of enhanced care for depression. *J Clin Epidemiol* 2008;61:160-8.

chaa006386.indd 36 31/01/2013 10:33:39

104 Lathyris D, Trikalinos TA, Ioannidis JPA. Evidence from crossover trials: Empirical evaluation and comparison against parallel arm trials. *Int J Epidemiol* 2007;36:422-30.
105 Khan KS, Daya S, Collins JA, Walter SD. Empirical evidence of bias in infertility research: overestimation of treatment effect in crossover trials using pregnancy as the outcome measure. *Fertil Steril* 1996;65:939-45.
106 Katz J, Finnerup NB, Dworkin RH. Clinical trial outcome in neuropathic pain: relationship to study characteristics. *Neurology* 2008;70:263-72.
107 Le Henanff A, Giraudeau B, Baron G, Ravaud P. Quality of reporting of noninferiority and equivalence randomized trials. *JAMA* 2006;295:1147-51.
108 Fleming TR, Odem-Davis K, Rothmann MD, Li SY. Some essential considerations in the design and conduct of non-inferiority trials. *Clin Trials* 2011;8:432-9.
109 Krysan DJ, Kemper AR. Claims of equivalence in randomized controlled trials of the treatment of bacterial meningitis in children. *Pediatr Infect Dis J* 2002;21:753-8.
110 Tinmouth JM, Steele LS, Tomlinson G, Glazier GH. Are claims of equivalency in digestive diseases trials supported by the evidence. *Gastroenterol* 2004;126:1700-10.
111 Kairalla JA, Coffey CS, Thomann MA, Muller KE. Adaptive trial designs: a review of barriers and opportunities. *Trials* 2012;13:145.
112 Dragalin V. Adaptive designs: terminology and classification. *Drug Inf J* 2006;40:425-35.
113 Project Accept Study Group. Project Accept (HPTN 043): A phase III randomized controlled trial of community mobilization, mobile testing, same-day results, and post-test support for HIV in Sub-Saharan Africa and Thailand [protocol]. Version 2.4 (April 15, 2011). www.hptn.org/research_studies/hptn043.asp.
114 Ford JG, Howerton MW, Lai GY, Gary TL, Bolen S, Gibbons MC, et al. Barriers to recruiting underrepresented populations to cancer clinical trials: a systematic review. *Cancer* 2008;112:228-42.
115 Elkins JS, Khatabi T, Fung L, Rootenberg J, Johnston SC. Recruiting subjects for acute stroke trials: a meta-analysis. *Stroke* 2006;37:123-8.
116 Heo M, Papademetriou E, Meyers BS. Design characteristics that influence attrition in geriatric antidepressant trials: meta-analysis. *Int J Geriatr Psychiatry* 2009;24:990-1001.
117 Fabricatore AN, Wadden TA, Moore RH, Butryn ML, Gravallese EA, Erondu NE, et al. Attrition from randomized controlled trials of pharmacological weight loss agents: a systematic review and analysis. *Obes Rev* 2009;10:333-41.
118 Lemieux J, Goodwin PJ, Pritchard KI, Gelmon KA, Bordeleau LJ, Duchesne T, et al. Identification of cancer care and protocol characteristics associated with recruitment in breast cancer clinical trials. *J Clin Oncol* 2008;26:4458-65.
119 Jones R, Jones RO, McCowan C, Montgomery AA, Fahey T, Jones R, et al. The external validity of published randomized controlled trials in primary care. *BMC Fam Pract* 2009;10:5.
120 Sood A, Knudsen K, Sood R, Wahner-Roedler DL, Barnes SA, Bardia A, et al. Publication bias for CAM trials in the highest impact factor medicine journals is partly due to geographical bias. *J Clin Epidemiol* 2007;60:1123-6.
121 Wu T, Li Y, Bian Z, Liu G, Moher D. Randomized trials published in some Chinese journals: how many are randomized? *Trials* 2009;10:46.
122 Hotopf M, Lewis G, Normand C. Putting trials on trial--the costs and consequences of small trials in depression: a systematic review of methodology. *J Epidemiol Community Health* 1997;51:354-8.
123 Evaluation study of congestive heart failure and pulmonary artery catheterization effectiveness (ESCAPE) [protocol]. Version 3.0 (November 29, 1999). https://biolincc.nhlbi.nih.gov/studies/escape/?q=escape.
124 Sandercock P, Lindley R, Wardlaw J, Dennis M, Lewis S, Venables G, et al. The third international stroke trial (IST-3) of thrombolysis for acute ischaemic stroke [protocol]. *Trials* 2008;9:37.
125 Blümle A, Meerpohl JJ, Rücker G, Antes G, Schumacher M, von Elm E. Reporting of eligibility criteria of randomised trials: cohort study comparing trial protocols with subsequent articles. *BMJ* 2011;342:d1828.
126 Cook JA. The challenges faced in the design, conduct and analysis of surgical randomised controlled trials. *Trials* 2009;10:9.
127 Simpson F, Sweetman EA, Doig GS. Systematic review of techniques and interventions for improving adherence to inclusion and exclusion criteria during enrolment into randomised controlled trials. *Trials* 2010;11:17.
128 Rendell JM, Merritt RK, Geddes JR. Incentives and disincentives to participation by clinicians in randomised controlled trials. *Cochrane Database Syst Rev* 2007;2:MR000021.
129 Weijer C. Characterizing the population in clinical trials: barriers, comparability, and implications for review. *Philosophy Publications*. Paper 250.1995. http://ir.lib.uwo.ca/philosophypub/250.
130 Townsley CA, Selby R, Siu LL. Systematic review of barriers to the recruitment of older patients with cancer onto clinical trials. *J Clin Oncol* 2005;23:3112-24.
131 Uchino K, Billheimer D, Cramer SC. Entry criteria and baseline characteristics predict outcome in acute stroke trials. *Stroke* 2001;32:909-16.
132 Van Spall HGC, Toren A, Kiss A, Fowler RA. Eligibility criteria of randomized controlled trials published in high-impact general medical journals: a systematic sampling review. *JAMA* 2007;297:1233-40.
133 Shapiro SH, Weijer C, Freedman B. Reporting the study populations of clinical trials. Clear transmission or static on the line? *J Clin Epimiol* 2000;53:973-9.
134 Gandhi M, Ameli N, Bacchetti P, Sharp GB, French AL, Young M, et al. Eligibility criteria for HIV clinical trials and generalizability of results: the gap between published reports and study protocols. *AIDS* 2005;19:1885-96.
135 Montori VM, Wang YG, Alonso-Coello P, Bhagra S. Systematic evaluation of the quality of randomized controlled trials in diabetes. *Diabetes Care* 2006;29:1833-8.
136 Mitchell SL, Sullivan EA, Lipsitz LA. Exclusion of elderly subjects from clinical trials for Parkinson disease. *Arch Neurol* 1997;54:1393-8.
137 Thorpe KE, Zwarenstein M, Oxman AD, Treweek S, Furberg CD, Altman DG, et al. A pragmatic-explanatory continuum indicator summary (PRECIS): a tool to help trial designers. *CMAJ* 2009;180:E47-57.
138 Blanco C, Olfson M, Goodwin RD, Ogburn E, Liebowitz MR, Nunes EV, et al. Generalizability of clinical trial results for major depression to community samples: results from the National Epidemiologic Survey on Alcohol and Related Conditions. *J Clin Psychiatry* 2008;69:1276-80.
139 Herland K, Akselsen JP, Skjønsberg OH, Bjermer L. How representative are clinical study patients with asthma or COPD for a larger "real life" population of patients with obstructive lung disease? *Respir Med* 2005;99:11-9.
140 Bartlett C, Doyal L, Ebrahim S, Davey P, Bachmann M, Egger M, et al. The causes and effects of socio-demographic exclusions from clinical trials. *Health Technol Assess* 2005;9:iii-iiv.
141 Zarin DA, Young JL, West JC. Challenges to evidence-based medicine: a comparison of patients and treatments in randomized controlled trials with patients and treatments in a practice research network. *Soc Psychiatry Psychiatr Epidemiol* 2005;40:27-35.
142 Hordijk-Trion M, Lenzen M, Wijns W, de Jaegere P, Simoons ML, Scholte op Reimer WJ, et al. Patients enrolled in coronary intervention trials are not representative of patients in clinical practice: results from the Euro Heart Survey on Coronary Revascularization. *Eur Heart J* 2006;27:671-8.
143 Kievit W, Fransen J, Oerlemans AJ, Kuper HH, van der Laar MA, de Rooij DJ, et al. The efficacy of anti-TNF in rheumatoid arthritis, a comparison between randomised controlled trials and clinical practice. *Ann Rheum Dis* 2007;66:1473-8.
144 Uijen AA, Bakx JC, Mokkink HG, van Weel C. Hypertension patients participating in trials differ in many aspects from patients treated in general practices. *J Clin Epidemiol* 2007;60:330-5.
145 Crossman DC, Morton AC, Gunn JP, Greenwood JP, Hall AS, Fox KA, et al. Investigation of the effect of Interleukin-1 receptor antagonist (IL-1ra) on markers of inflammation in non-ST elevation acute coronary syndromes (The MRC-ILA-HEART Study) [protocol]. *Trials* 2008;9:8.
146 Glasziou P, Meats E, Heneghan C, Shepperd S. What is missing from descriptions of treatment in trials and reviews? *BMJ* 2008;336:1472-4.
147 Duff JM, Leather H, Walden EO, LaPlant KD, George TJ, Jr. Adequacy of published oncology randomized controlled trials to provide therapeutic details needed for clinical application. *J Natl Cancer Inst* 2010;102:702-5.
148 Chalmers I, Glasziou P. Avoidable waste in the production and reporting of research evidence. *Lancet* 2009;374:86-9.
149 Glasziou P, Chalmers I, Altman DG, Bastian H, Boutron I, Brice A, et al. Taking healthcare interventions from trial to practice. *BMJ* 2010;341:c3852.
150 Golomb BA, Erickson LC, Koperski S, Sack D, Enkin M, Howick J. What's in placebos: who knows? Analysis of randomized, controlled trials. *Ann Intern Med* 2010;153:532-5.
151 Medical Research Council Working Party on Prostate Cancer. MRC PR05. A Medical Research Council randomised trial of adjuvant sodium clodronate in patients commencing or responding to hormone therapy for metastatic prostate adenocarcinoma [protocol]. Feb 1995 version. www.ctu.mrc.ac.uk/research_areas/study_details.aspx?s=60.
152 Panel on Handling Missing Data in Clinical Trials, National Research Council. The prevention and treatment of missing data in clinical trials. Washington DC, National Academies Press, 2010.
153 Buchbinder S, Liu A, Thompson M, Mayer K. Phase II extended safety study of tenofovir disoproxil fumarate (TDF) among HIV-1 negative men [protocol]. Version 1.6 (February 16, 2007). www.plosone.org/article/info%3Adoi%2F10.1371%2Fjournal.pone.0023688.
154 World Health Organization. Adherence to long-term therapies: evidence for action. 2012. www.who.int/chp/knowledge/publications/adherence_full_report.pdf.
155 Osterberg L, Blaschke T. Adherence to medication. *N Engl J Med* 2005;353:487-97.
156 Smith D. Patient nonadherence in clinical trials: could there be a link to postmarketing patient safety? *Drug Inf J* 2012;46:27-34.
157 Robiner WN. Enhancing adherence in clinical research. *Contemp Clin Trials* 2005;26:59-77.
158 Matsui D. Strategies to measure and improve patient adherence in clinical trials. *Pharmaceut Med* 2009;23:289-97.

chaa006386.indd 37 31/01/2013 10:33:39

159 Simpson SH, Eurich DT, Majumdar SR, Padwal RS, Tsuyuki RT, Varney J, et al. A meta-analysis of the association between adherence to drug therapy and mortality. *BMJ* 2006;333:15.
160 International Conference on Harmonisation. ICH Harmonised Tripartite Guideline: Good clinical practice, consolidated guideline. International Conference on Harmonisation of Technical Requirements for Registration of Pharmaceuticals for Human Use (June 1996, E6). 1996. http://www.ich.org/fileadmin/Public_Web_Site/ICH_Products/Guidelines/Efficacy/E6_R1/Step4/E6_R1__Guideline.pdf.
161 Jayaraman S, Rieder MJ, Matsui DM. Compliance assessment in drug trials: has there been improvement in two decades? *Can J Clin Pharmacol* 2005;12:e251-3.
162 Sackett DL. Clinician-trialist rounds: 5. Cointervention bias--how to diagnose it in their trial and prevent it in yours. *Clin Trials* 2011;8:440-2.
163 Zarin DA, Tse T, Williams RJ, Califf RM, Ide NC. The ClinicalTrials.gov results database--update and key issues. *N Engl J Med* 2011;364:852-60.
164 Bhandari M, Lochner H, Tornetta P, III. Effect of continuous versus dichotomous outcome variables on study power when sample sizes of orthopaedic randomized trials are small. *Arch Orthop Trauma Surg* 2002;122:96-8.
165 Verhagen AP, de Vet HCW, Willemsen S, Stijnen T. A meta-regression analysis shows no impact of design characteristics on outcome in trials on tension-type headaches. *J Clin Epi* 2008;61:813-8.
166 Hróbjartsson A, Thomsen AS, Emanuelsson F, Tendal B, Hilden J, Boutron I, et al. Observer bias in randomised clinical trials with binary outcomes: systematic review of trials with both blinded and non-blinded outcome assessors. *BMJ* 2012;344:e1119.
167 Savović J, Jones HE, Altman DG, Harris RJ, Jüni P, Pildal J, et al. Influence of reported study design characteristics on intervention effect estimates from randomized, controlled trials. *Ann Intern Med* 2012;157:429-8.
168 Ferreira-González I, Busse JW, Heels-Ansdell D, Montori VM, Akl EA, Bryant DM, et al. Problems with use of composite end points in cardiovascular trials: systematic review of randomised controlled trials. *BMJ* 2007;334:786.
169 Montori VM, Permanyer-Miralda G, Ferreira-González I, Busse JW, Pacheco-Huergo V, Bryant D, et al. Validity of composite end points in clinical trials. *BMJ* 2005;330:596.
170 Freemantle N, Calvert M, Wood J, Eastaugh J, Griffin C. Composite outcomes in randomized trials: greater precision but with greater uncertainty? *JAMA* 2003;289:2554-59.
171 Cordoba G, Schwartz L, Woloshin S, Bae H, Gøtzsche PC. Definition, reporting, and interpretation of composite outcomes in clinical trials: systematic review. *BMJ* 2010;341:c3920.
172 Dwan K, Altman DG, Arnaiz JA, Bloom J, Chan A-W, Cronin E, et al. Systematic review of the empirical evidence of study publication bias and outcome reporting bias. *PLoS One* 2008;3:e3081.
173 Rising K, Bacchetti P, Bero L. Reporting bias in drug trials submitted to the Food and Drug Administration: Review of publication and presentation. *PLoS Med* 2008;5:e217.
174 Turner EH, Matthews AM, Linardatos E, Tell RA, Rosenthal R. Selective publication of antidepressant trials and its influence on apparent efficacy. *N Engl J Med* 2008;358:252-60.
175 Vedula SS, Bero L, Scherer RW, Dickersin K. Outcome reporting in industry-sponsored trials of gabapentin for off-label use. *N Engl J Med* 2009;361:1963-71.
176 Dwan K, Altman DG, Cresswell L, Blundell M, Gamble CL, Williamson PR. Comparison of protocols and registry entries to published reports for randomised controlled trials. *Cochrane Database Syst Rev* 2011;1:MR000031.
177 Chan A-W. Access to clinical trial data. *BMJ* 2011;342:d80.
178 Tugwell P, Boers M, Brooks P, Simon L, Strand V, Idzerda L. OMERACT: an international initiative to improve outcome measurement in rheumatology. *Trials* 2007;8:38.
179 Williamson P, Altman D, Blazeby J, Clarke M, Gargon E. Driving up the quality and relevance of research through the use of agreed core outcomes. *J Health Serv Res Policy* 2012;17:1-2.
180 Clarke M. Standardising outcomes for clinical trials and systematic reviews. *Trials* 2007;8:39.
181 Booth R, Fuller B, Thompson L, McCarty D, Shoptaw S, et al. STUDY #: NIDA-CTN-0017. HIV and HCV risk reduction interventions in drug detoxification and treatment settings [protocol]. Version 4.0 (August 16, 2010). www.dtmi.duke.edu/crflibrary-demo/crf-library-1/crf-library/trials-a-e/ctn-0017.
182 Cockayne NL, Glozier N, Naismith SL, Christensen H, Neal B, Hickie IB. Internet-based treatment for older adults with depression and co-morbid cardiovascular disease: protocol for a randomised, double-blind, placebo controlled trial [protocol]. *BMC Psychiatry* 2011;11:10.
183 McMurran M, Crawford MJ, Reilly JG, McCrone P, Moran P, Williams H, et al. Psycho-education with problem solving (PEPS) therapy for adults with personality disorder: A pragmatic multi-site community-based randomised clinical trial [protocol]. *Trials* 2011;12:198.
184 van der Lee JH, Wesseling J, Tanck MW, Offringa M. Efficient ways exist to obtain the optimal sample size in clinical trials in rare diseases. *J Clin Epidemiol* 2008;61:324-30.
185 Yazici Y, Adler NM, Yazici H. Most tumour necrosis factor inhibitor trials in rheumatology are undeservedly called 'efficacy and safety' trials: a survey of power considerations. *Rheumatol* 2008;47:1054-7.
186 Hernández AV, Boersma E, Murray GD, Habbema JD, Steyerberg EW. Subgroup analyses in therapeutic cardiovascular clinical trials: are most of them misleading? *Am Heart J* 2006;151:257-64.
187 Copay AG, Subach BR, Glassman SD, Polly DW Jr, Schuler TC. Understanding the minimum clinically important difference: a review of concepts and methods. *Spine J* 2007;7:541-6.
188 Raju TN, Langenberg P, Sen A, Aldana O. How much 'better' is good enough? The magnitude of treatment effect in clinical trials. *Am J Dis Child* 1992;146:407-11.
189 Charles P, Giraudeau B, Dechartres A, Baron G, Ravaud P. Reporting of sample size calculation in randomised controlled trials: review. *BMJ* 2009;338:b1732.
190 Vickers AJ. Underpowering in randomized trials reporting a sample size calculation. *J Clin Epidemiol* 2003;56:717-20.
191 Proschan MA. Sample size re-estimation in clinical trials. *Biom J* 2009;51:348-57.
192 Julious SA, Campbell MJ, Altman DG. Estimating sample sizes for continuous, binary, and ordinal outcomes in paired comparisons: practical hints. *J Biopharm Stat* 1999;9:241-51.
193 Campbell MK, Elbourne DR, Altman DG, CONSORT group. CONSORT statement: extension to cluster randomised trials. *BMJ* 2004;328:702-8.
194 Piaggio G, Elbourne DR, Altman DG, Pocock SJ, Evans SJW. Reporting of noninferiority and equivalence randomized trials: An extension of the CONSORT statement. *JAMA* 2006;295:1152-60.
195 Pals SL, Murray DM, Alfano CM, Shadish WR, Hannan PJ, Baker WL. Individually randomized group treatment trials: a critical appraisal of frequently used design and analytic approaches. *Am J Pub Health* 2008;98:1418-24.
196 Eldridge S, Ashby D, Bennett C, Wakelin M, Feder G. Internal and external validity of cluster randomised trials: Systematic review of recent trials. *BMJ* 2008;336:876-80.
197 Eldridge SM, Ashby D, Feder GS, Rudnicka AR, Ukoumunne OC. Lessons for cluster randomized trials in the twenty-first century: a systematic review of trials in primary care. *Clin Trials* 2004;1:80-90.
198 Murray DM, Pals SL, Blitstein JL, Alfano CM, Lehman J. Design and analysis of group-randomized trials in cancer: A review of current practices. *J Natl Cancer Inst* 2008;100:483-91.
199 Freiman JA, Chalmers TC, Smith H, Jr., Kuebler RR. The importance of beta, the type II error and sample size in the design and interpretation of the randomized control trial. Survey of 71 "negative" trials. *N Engl J Med* 1978;299:690-4.
200 Bailey CS, Fisher CG, Dvorak MF. Type II error in the spine surgical literature. *Spine* 2004;29:1146-9.
201 Lochner HV, Bhandari M, Tornetta P, III. Type-II error rates (beta errors) of randomized trials in orthopaedic trauma. *J Bone Joint Surg Am* 2001;83-A:1650-5.
202 Enwere G. A review of the quality of randomized clinical trials of adjunctive therapy for the treatment of cerebral malaria. *Trop Med Int Health* 2005;10:1171-5.
203 Breau RH, Carnat TA, Gaboury I. Inadequate statistical power of negative clinical trials in urological literature. *J Urol* 2006;176:263-6.
204 Keen HI, Pile K, Hill CL. The prevalence of underpowered randomized clinical trials in rheumatology. *J Rheumatol* 2005;32:2083-8.
205 Maggard MA, O'Connell JB, Liu JH, Etzioni DA, Ko CY. Sample size calculations in surgery: are they done correctly? *Surgery* 2003;134:275-9.
206 Dimick JB, Diener-West M, Lipsett PA. Negative results of randomized clinical trials published in the surgical literature: equivalency or error? *Arch Surg* 2001;136:796-800.
207 Murray GD. Research governance must focus on research training. *BMJ* 2001;322:1461-2.
208 Asthma Clinical Research Network. Beta Adrenergic Response by Genotype (BARGE) study protocol: a study to compare the effects of regularly scheduled use of inhaled albuterol in patients with mild to moderate asthma who are members of two distinct haplotypes expressed at the β2 -adrenergic receptor [protocol]. Version 5.4 (September 23, 1999). https://biolincc.nhlbi.nih.gov/studies/barge/?q=barge.
209 Campbell MK, Snowdon C, Francis D, Elbourne D, McDonald AM, Knights R, et al. Recruitment to randomised trials: Strategies for trial enrolment and participation study. The STEPS study. *Health Technol Assess* 2007;11:iii-72.
210 Wise P, Drury M. Pharmaceutical trials in general practice: the first 100 protocols. An audit by the clinical research ethics committee of the Royal College of General Practitioners. *BMJ* 1996;313:1245-8.
211 Pich J, Carné X, Arnaiz JA, Gómez B, Trilla A, Rodés J. Role of a research ethics committee in follow-up and publication of results. *Lancet* 2003;361:1015-6.
212 Decullier E, Lhéritier V, Chapuis F. Fate of biomedical research protocols and publication bias in France: retrospective cohort study. *BMJ* 2005;331:19.
213 Dal-Ré R, Ortega R, Espada J. [Efficiency of investigators in recruitment of patients for clinical trials: apropos of a multinational study]. *Med Clin (Barc)* 1998;110:521-3.

214 McDonald AM, Knight RC, Campbell MK, Entwistle VA, Grant AM, Cook JA, et al. What influences recruitment to randomised controlled trials? A review of trials funded by two UK funding agencies. *Trials* 2006;7:9.
215 Charlson ME, Horwitz RI. Applying results of randomised trials to clinical practice: impact of losses before randomisation. *BMJ* 1984;289:1281-4.
216 Caldwell PH, Hamilton S, Tan A, Craig JC. Strategies for increasing recruitment to randomised controlled trials: systematic review. *PLoS Med* 2010;7:e1000368.
217 Treweek S, Pitkethly M, Cook J, Kjeldstrøm M, Taskila T, Johansen M, et al. Strategies to improve recruitment to randomised controlled trials. *Cochrane Database Syst Rev* 2010;4:MR000013.
218 Abraham NS, Young JM, Solomon MJ. A systematic review of reasons for nonentry of eligible patients into surgical randomized controlled trials. *Surgery* 2006;139:469-83.
219 Lai GY, Gary TL, Tilburt J, Bolen S, Baffi C, Wilson RF, et al. Effectiveness of strategies to recruit underrepresented populations into cancer clinical trials. *Clin Trials* 2006;3:133-41.
220 UyBico SJ, Pavel S, Gross CP. Recruiting vulnerable populations into research: a systematic review of recruitment interventions. *J Gen Intern Med* 2007;22:852-63.
221 Miller NL, Markowitz JC, Kocsis JH, Leon AC, Brisco ST, Garno JL. Cost effectiveness of screening for clinical trials by research assistants versus senior investigators. *J Psychiatr Res* 1999;33:81-5.
222 Tworoger SS, Yasui Y, Ulrich CM, Nakamura H, LaCroix K, Johnston R, et al. Mailing strategies and recruitment into an intervention trial of the exercise effect on breast cancer biomarkers. *Cancer Epidemiol Biomarkers Prev* 2002;11:73-7.
223 Schroy P.C. 3rd, Glick JT, Robinson P, Lydotes MA, Heeren TC, Prout M, et al. A cost-effectiveness analysis of subject recruitment strategies in the HIPAA era: results from a colorectal cancer screening adherence trial. *Clin Trials* 2009;6:597-609.
224 Harvey LA, Dunlop SA, Churilov L, Hsueh Y-SA, Galea MP. Early intensive hand rehabilitation after spinal cord injury ("hands on"): a protocol for a randomised controlled trial [protocol]. *Trials* 2011;12:14.
225 Schulz KF, Grimes DA. The Lancet handbook of essential concepts in clinical research. Elsevier, 2006.
226 Greenland S. Randomization, statistics, and causal inference. *Epidemiol* 1990;1:421-9.
227 Armitage P. The role of randomization in clinical trials. *Stat Med* 1982;1:345-52.
228 Odgaard-Jensen J, Vist GE, Timmer A, Kunz R, Akl EA, Schünemann H, et al. Randomisation to protect against selection bias in healthcare trials. *Cochrane Database Syst Rev* 2011;4:MR000012.
229 Jüni P, Altman DG, Egger M. Systematic reviews in health care: assessing the quality of controlled clinical trials. *BMJ* 2001;323:42-6.
230 McEntegart DJ. The pursuit of balance using stratified and dynamic randomization techniques: an overview. *Drug Inf J* 2003;37:293-308.
231 Schulz KF, Grimes DA. Generation of allocation sequences in randomised trials: chance, not choice. *Lancet* 2002;359:515-9.
232 Altman DG, Bland JM. How to randomise. *BMJ* 1999;319:703-4.
233 Schulz KF, Chalmers I, Hayes RJ, Altman DG. Empirical evidence of bias. Dimensions of methodological quality associated with estimates of treatment effects in controlled trials. *JAMA* 1995;273:408-12.
234 Kernan WN, Viscoli CM, Makuch RW, Brass LM, Horwitz RI. Stratified randomization for clinical trials. *J Clin Epidemiol* 1999;52:19-26.
235 Han B, Enas NH, McEntegart D. Randomization by minimization for unbalanced treatment allocation. *Stat Med* 2009;28:3329-46.
236 Altman DG. Practical statistics for medical research. Chapman and Hall/CRC, 1991.
237 Treasure T, MacRae KD. Minimisation: the platinum standard for trials? Randomisation doesn't guarantee similarity of groups; minimisation does. *BMJ* 1998;317:362-3.
238 Berger VW. Varying the block size does not conceal the allocation. *J Crit Care* 2006;21:229-30.
239 Berger VW. Minimization, by its nature, precludes allocation concealment, and invites selection bias. *Contemp Clin Trials* 2010;31:406.
240 Abbott JH, Robertson MC, McKenzie JE, Baxter GD, Theis J-C, Campbell AJ, et al. Exercise therapy, manual therapy, or both, for osteoarthritis of the hip or knee: a factorial randomised controlled trial protocol [protocol]. *Trials* 2009;10:11.
241 Schulz KF, Grimes DA. Allocation concealment in randomised trials: defending against deciphering. *Lancet* 2002;359:614-618.
242 Chalmers TC, Levin H, Sacks HS, Reitman D, Berrier J, Nagalingam R. Meta-analysis of clinical trials as a scientific discipline. I: Control of bias and comparison with large co-operative trials. *Stat Med* 1987;6:315-28.
243 Schulz KF, Chalmers I, Grimes DA, Altman DG. Assessing the quality of randomization from reports of controlled trials published in obstetrics and gynecology journals. *JAMA* 1994;272:125-8.
244 Herbison P, Hay-Smith J, Gillespie WJ. Different methods of allocation to groups in randomized trials are associated with different levels of bias. A meta-epidemiological study. *J Clin Epidemiol* 2011;64:1070-5.
245 Kunz R, Vist G, Oxman AD. Randomisation to protect against selection bias in healthcare trials. *Cochrane Database Syst Rev* 2007;2:MR000012.
246 Klingberg S, Wittorf A, Meisner C, Wölwer W, Wiedemann G, Herrlich J, et al. Cognitive behavioural therapy versus supportive therapy for persistent positive symptoms in psychotic disorders: The POSITIVE study, a multicenter, prospective, single-blind, randomised controlled clinical trial [protocol]. *Trials* 2010;11:123.
247 Dalum HS, Korsbek L, Mikkelsen JH, Thomsen K, Kistrup K, Olander M, et al. Illness management and recovery (IMR) in Danish community mental health centres [protocol]. *Trials* 2011;12:195.
248 Hróbjartsson A, Gøtzsche PC. Placebo interventions for all clinical conditions. *Cochrane Database Syst Rev* 2010;1:CD003974.
249 Tierney JF, Stewart LA. Investigating patient exclusion bias in meta-analysis. *Int J Epidemiol* 2005;34:79-87.
250 Nüesch E, Trelle S, Reichenbach S, Rutjes AW, Bürgi E, Scherer M, et al. The effects of excluding patients from the analysis in randomised controlled trials: meta-epidemiological study. *BMJ* 2009;339:b3244.
251 Schulz KF, Chalmers I, Altman DG. The landscape and lexicon of blinding in randomized trials. *Ann Intern Med* 2002;136:254-59.
252 Ballintine EJ. Randomized controlled clinical trial. National Eye Institute workshop for ophthalmologists. Objective measurements and the double-masked procedure. *Am J Ophthalmol* 1975;79:763-7.
253 Gøtzsche PC. Blinding during data analysis and writing of manuscripts. *Control Clin Trials* 1996;17:285-90.
254 Grant AM, Altman DG, Babiker AB, Campbell MK, Clemens FJ, Darbyshire JH, et al. Issues in data monitoring and interim analysis of trials. *Health Technol Assess* 2005;9:1-238.
255 Meinert CL. Masked monitoring in clinical trials—blind stupidity? *N Engl J Med* 1998;338:1381-2.
256 Boutron I, Estellat C, Guittet L, Dechartres A, Sackett DL, Hróbjartsson A, et al. Methods of blinding in reports of randomized controlled trials assessing pharmacological treatments: a systematic review. *PLoS Med* 2006;3:e425.
257 Boutron I, Guittet L, Estellat C, Moher D, Hróbjartsson A, Ravaud P. Reporting methods of blinding in randomized trials assessing nonpharmacological treatments. *PLoS Med* 2007;4:e61.
258 Lieverse R, Nielen MM, Veltman DJ, Uitdehaag BM, van Someren EJ, Smit JH, et al. Bright light in elderly subjects with nonseasonal major depressive disorder: a double blind randomised clinical trial using early morning bright blue light comparing dim red light treatment. *Trials* 2008;9:48.
259 Devereaux PJ, Manns BJ, Ghali WA, Quan H, Lacchetti C, Montori VM, et al. Physician interpretations and textbook definitions of blinding terminology in randomized controlled trials. *JAMA* 2001;285:2000-3.
260 Haahr MT, Hróbjartsson A. Who is blinded in randomized clinical trials? A study of 200 trials and a survey of authors. *Clin Trials* 2006;3:360-5.
261 Hróbjartsson A, Boutron I. Blinding in randomized clinical trials: imposed impartiality. *Clin Pharmacol Ther* 2011;90:732-6.
262 Fergusson D, Glass KC, Waring D, Shapiro S. Turning a blind eye: the success of blinding reported in a random sample of randomised, placebo controlled trials. *BMJ* 2004;328:432.
263 Sackett DL. Clinician-trialist rounds: 6. Testing for blindness at the end of your trial is a mug's game. *Clin Trials* 2011;8:674-6.
264 Schulz KF, Altman DG, Moher D, Fergusson D. CONSORT 2010 changes and testing blindness in RCTs. *Lancet* 2010;375:1144-6.
265 A randomized, double blind, placebo controlled, parallel group trial for assessing the clinical benefit of Dronedarone 400mg BID on top of standard therapy in patients with permanent atrial fibrillation and additional risk factors. Permanent Atrial fibriLLAtion outcome Study using Dronedarone on top of standard therapy (PALLAS) [protocol]. Version 1 (February 26, 2010). www.nejm.org/doi/full/10.1056/NEJMoa1109867.
266 Campbell NL, Khan BA, Farber M, Campbell T, Perkins AJ, Hui SL, et al. Improving delirium care in the intensive care unit: the design of a pragmatic study [protocol]. *Trials* 2011;12:139.
267 FSGS - Clinical trial [protocol]. Version 3c (June 20, 2005). https://clinicalresearch.ccf.org/fsgs/docs/index_docs.html.
268 Lane SJ, Heddle NM, Arnold E, Walker I. A review of randomized controlled trials comparing the effectiveness of hand held computers with paper methods for data collection. *BMC Med Inform Decis Mak* 2006;6:23.
269 Bent S, Padula A, Avins AL. Brief communication: Better ways to question patients about adverse medical events: a randomized, controlled trial. *Ann Intern Med* 2006;144:257-61.
270 Dale O, Hagen KB. Despite technical problems personal digital assistants outperform pen and paper when collecting patient diary data. *J Clin Epidemiol* 2007;60:8-17.
271 Litchfield J, Freeman J, Schou H, Elsley M, Fuller R, Chubb B. Is the future for clinical trials internet-based? A cluster randomised clinical trial. *Clin Trials* 2005;2:72-9.
272 Bedard M, Molloy DW, Standish T, Guyatt GH, D'Souza J, Mondadori C, et al. Clinical trials in cognitively impaired older adults: home versus clinic assessments. *J Am Geriatr Soc* 1995;43:1127-30.
273 Jasperse DM, Ahmed SW. The Mid-Atlantic Oncology Program's comparison of two data collection methods. *Control Clin Trials* 1989;10:282-9.
274 Basch E, Jia X, Heller G, Barz A, Sit L, Fruscione M, et al. Adverse symptom event reporting by patients vs clinicians: relationships with clinical outcomes. *J Natl Cancer Inst* 2009;101:1624-32.

chaa006386.indd 39 31/01/2013 10:33:39

275 Cohen SB, Strand V, Aguilar D, Ofman JJ. Patient- versus physician-reported outcomes in rheumatoid arthritis patients treated with recombinant interleukin-1 receptor antagonist (anakinra) therapy. *Rheumatology (Oxford)* 2004;43:704-11.
276 Fromme EK, Eilers KM, Mori M, Hsieh YC, Beer TM. How accurate is clinician reporting of chemotherapy adverse effects? A comparison with patient-reported symptoms from the Quality-of-Life Questionnaire C30. *J Clin Oncol* 2004;22:3485-90.
277 Walther B, Hossin S, Townend J, Abernethy N, Parker D, Jeffries D. Comparison of electronic data capture (EDC) with the standard data capture method for clinical trial data. *PLoS One* 2011;6:e25348.
278 Kryworuchko J, Stacey D, Bennett C, Graham ID. Appraisal of primary outcome measures used in trials of patient decision support. *Patient Educ Couns* 2008;73:497-503.
279 Roberts L, Counsell C. Assessment of clinical outcomes in acute stroke trials. *Stroke* 1998;29:986-91.
280 Marshall M, Lockwood A, Bradley C, Adams C, Joy C, Fenton M. Unpublished rating scales: a major source of bias in randomised controlled trials of treatments for schizophrenia. *Br J Psychiatry* 2000;176:249-52.
281 Williams GW. The other side of clinical trial monitoring; assuring data quality and procedural adherence. *Clin Trials* 2006;3:530-7.
282 Gassman JJ, Owen WW, Kuntz TE, Martin JP, Amoroso WP. Data quality assurance, monitoring, and reporting. *Control Clin Trials* 1995;16:104S-36S.
283 Meyerson LJ, Wiens BL, LaVange LM, Koutsoukos AD. Quality control of oncology clinical trials. *Hematol Oncol Clin North Am* 2000;14:953-71.
284 Fong DYT. Data management and quality assurance. *Drug Inf J* 2001;35:839-44.
285 Knatterud GL, Rockhold FW, George SL, Barton FB, Davis CE, Fairweather WR, et al. Guidelines for quality assurance in multicenter trials: a position paper. *Control Clin Trials* 1998;19:477-93.
286 Prevention Study Group. HEALTHY primary prevention trial protocol [protocol]. Version 1.4 (July 14, 2008). www.healthystudy.org/.
287 HIV Prevention Trials Network and the International Maternal Pediatric and Adolescent AIDS Clinical Trials Network. HPTN 046: A phase III trial to determine the efficacy and safety of an extended regimen of nevirapine in infants born to HIV-infected women to prevent vertical HIV transmission during breastfeeding [protocol]. Version 3.0 (September 26, 2007). www.hptn.org/research_studies/hptn046.asp.
288 Ioannidis JP, Bassett R, Hughes MD, Volberding PA, Sacks HS, Lau J. Predictors and impact of patients lost to follow-up in a long-term randomized trial of immediate versus deferred antiretroviral treatment. *J Acquir Immune Defic Syndr Hum Retrovirol* 1997;16:22-30.
289 Ford ME, Havstad S, Vernon SW, Davis SD, Kroll D, Lamerato L, et al. Enhancing adherence among older African American men enrolled in a longitudinal cancer screening trial. *Gerontologist* 2006;46:545-50.
290 Couper MP, Peytchev A, Strecher VJ, Rothert K, Anderson J. Following up nonrespondents to an online weight management intervention: Randomized trial comparing mail versus telephone. *J Med Internet Res* 2007;9:e16.
291 Renfroe EG, Heywood G, Foreman L, Schron E, Powell J, Baessler C, et al. The end-of-study patient survey: methods influencing response rate in the AVID Trial. *Control Clin Trials* 2002;23:521-33.
292 Robinson KA, Dennison CR, Wayman DM, Pronovost PJ, Needham DM. Systematic review identifies number of strategies important for retaining study participants. *J Clin Epi* 2007;60:757-65.
293 Fleming TR. Addressing missing data in clinical trials. *Ann Intern Med* 2011;154:113-7.
294 Liu M, Wei L, Zhang J. Review of guidelines and literature for handling missing data in longitudinal clinical trials with a case study. *Pharm Stat* 2006;5:7-18.
295 Wahlbeck K, Tuunainen A, Ahokas A, Leucht S. Dropout rates in randomised antipsychotic drug trials. *Psychopharmacology (Berl)* 2001;155:230-33.
296 Kawado M, Hinotsu S, Matsuyama Y, Yamaguchi T, Hashimoto S, Ohashi Y. A comparison of error detection rates between the reading aloud method and the double data entry method. *Control Clin Trials* 2003;24:560-9.
297 Day S, Fayers P, Harvey D. Double data entry: what value, what price? *Control Clin Trials* 1998;19:15-24.
298 Reynolds-Haertle RA, McBride R. Single vs. double data entry in CAST. *Control Clin Trials* 1992;13:487-94.
299 Gibson D, Harvey AJ, Everett V, Parmar MK. Is double data entry necessary? The CHART trials. CHART Steering Committee. Continuous, hyperfractionated, accelerated radiotherapy. *Control Clin Trials* 1994;15:482-8.
300 Ioannidis JPA, Evans SJW, Gøtzsche PC, O'Neill RT, Altman DG, Schulz KF, et al. Better reporting of harms in randomized trials: an extension of the CONSORT statement. *Ann Intern Med* 2004;141:781-8.
301 Schulz KF, Grimes DA. Multiplicity in randomised trials I: endpoints and treatments. *Lancet* 2005;365:1591-5.
302 Tendal B, Nüesch E, Higgins JP, Jüni P, Gøtzsche PC. Multiplicity of data in trial reports and the reliability of meta-analyses: empirical study. *BMJ* 2011;343:d4829.
303 Flow Investigators. Fluid lavage of open wounds (FLOW): design and rationale for a large, multicenter collaborative 2 x 3 factorial trial of irrigating pressures and solutions in patients with open fractures [protocol]. *BMC Musculoskelet Disord* 2010;11:85.
304 Resuscitation Outcomes Consortium Prehospital Resuscitation using an IMpedance valve and Early vs Delayed analysis (ROC PRIMED) Trial. A factorial design of an active impedence threshold valve versus sham valve and analyze later versus analyze early [protocol]. Dec 2006 version. www.nejm.org/doi/full/10.1056/NEJMoa1010821.
305 Boonacker CW, Hoes AW, van Liere-Visser K, Schilder AG, Rovers MM. A comparison of subgroup analyses in grant applications and publications. *Am J Epidemiol* 2011;174:219-25.
306 Schulz KF, Grimes DA. Multiplicity in randomised trials II: subgroup and interim analyses. *Lancet* 2005;365:1657-61.
307 Hirji KF, Fagerland MW. Outcome based subgroup analysis: a neglected concern. *Trials* 2009;10:33.
308 Sun X, Briel M, Walter SD, Guyatt GH. Is a subgroup effect believable? Updating criteria to evaluate the credibility of subgroup analyses. *BMJ* 2010;340:c117.
309 Rothwell PM. Treating individuals 2. Subgroup analysis in randomised controlled trials: importance, indications, and interpretation. *Lancet* 2005;365:176-86.
310 Yu L-M, Chan A-W, Hopewell S, Deeks JJ, Altman DG. Reporting on covariate adjustment in randomised controlled trials before and after revision of the 2001 CONSORT statement: a literature review. *Trials* 2010;11:59.
311 Chen X, Liu M, Zhang J. A note on postrandomization adjustment of covariates. *Drug Inf J* 2005;39:373-83.
312 Rochon J. Issues in adjusting for covariates arising postrandomization in clinical trials. *Drug Inf J* 1999;33:1219-28.
313 Mohr JP, Moskowitz A, Ascheim D, Gelijns A, Parides M, et al. A Randomized multicenter clinical trial of unruptured brain AVMs (ARUBA): clinical protocol [protocol]. Version 3.0 (October 16, 2008). http://research.ncl.ac.uk/nctu/ARUBA.html.
314 Abraha I, Montedori A. Modified intention to treat reporting in randomised controlled trials: systematic review. *BMJ* 2010;340:c2697.
315 Fergusson D, Aaron SD, Guyatt G, Hébert P. Post-randomisation exclusions: the intention to treat principle and excluding patients from analysis. *BMJ* 2002;325:652-4.
316 Hollis S, Campbell F. What is meant by intention to treat analysis? Survey of published randomised controlled trials. *BMJ* 1999;319:670-4.
317 Akl EA, Briel M, You JJ, Sun X, Johnston BC, Busse JW, et al. Potential impact on estimated treatment effects of information lost to follow-up in randomised controlled trials (LOST-IT): systematic review. *BMJ* 2012;344:e2809.
318 Wood AM, White IR, Thompson SG. Are missing outcome data adequately handled? A review of published randomized controlled trials in major medical journals. *Clin Trials* 2004;1:368-76.
319 Fielding S, Fayers P, Ramsay CR. Analysing randomised controlled trials with missing data: Choice of approach affects conclusions. *Contemp Clin Trials* 2012;33:461-9.
320 Streiner DL. Missing data and the trouble with LOCF. *Evid Based Ment Health* 2008;11:3-5.
321 Sterne JA, White IR, Carlin JB, Spratt M, Royston P, Kenward MG, et al. Multiple imputation for missing data in epidemiological and clinical research: potential and pitfalls. *BMJ* 2009;338:b2393.
322 Groenwold RH, Donders AR, Roes KC, Harrell FE, Jr., Moons KG. Dealing with missing outcome data in randomized trials and observational studies. *Am J Epidemiol* 2012;175:210-7.
323 Giraudeau B, Ravaud P. Preventing bias in cluster randomised trials. *PLoS Med* 2009;6:e1000065.
324 Berger VW. Conservative handling of missing data. *Contemp Clin Trials* 2012;33:460.
325 Azuara-Blanco A, Burr JM, Cochran C, Ramsay C, Vale L, Foster P, et al. The effectiveness of early lens extraction with intraocular lens implantation for the treatment of primary angle-closure glaucoma (EAGLE): study protocol for a randomized controlled trial [protocol]. *Trials* 2011;12:133.
326 Sydes MR, Altman DG, Babiker AB, Parmar MK, Spiegelhalter DJ, DAMOCLES Group. Reported use of data monitoring committees in the main published reports of randomized controlled trials: a cross-sectional study. *Clin Trials* 2004;1:48-59.
327 Floriani I, Rotmensz N, Albertazzi E, Torri V, De Rosa M, Tomino C, et al. Approaches to interim analysis of cancer randomised clinical trials with time to event endpoints: a survey from the Italian National Monitoring Centre for Clinical Trials. *Trials* 2008;9:46.
328 Califf RM, Zarin DA, Kramer JM, Sherman RE, Aberle LH, Tasneem A. Characteristics of clinical trials registered in ClinicalTrials.gov, 2007-2010. *JAMA* 2012;307:1838-47.
329 Ellenberg SS. Independent data monitoring committees: rationale, operations and controversies. *Stat Med* 2001;20:2573-2583.
330 Ellenberg SS, Fleming TR, DeMets DL. Data monitoring committees in clinical trials: a practical perspective. 6th ed. Wiley, 2002.
331 DAMOCLES study group, NHS Health Technology Assessment Programme. A proposed charter for clinical trial data monitoring committees: helping them to do their job well. *Lancet* 2005;365:711-22.
332 Bakker OJ, van Santvoort HC, van Brunschot S, Ali UA, Besselink MG, et al. Pancreatitis, very early compared with normal start of enteral feeding (PYTHON trial): design and rationale of a randomised controlled multicenter trial [protocol]. *Trials* 2011;12:73.
333 DeMets DL, Pocock SJ, Julian DG. The agonising negative trend in monitoring of clinical trials. *Lancet* 1999;354:1983-8.

334 Berry DA. Interim analyses in clinical trials: classical vs. Bayesian approaches. *Stat Med* 1985;4:521-6.
335 Pocock SJ. When to stop a clinical trial. *BMJ* 1992;305:235-40.
336 Aronson JK, Ferner RE. Clarification of terminology in drug safety. *Drug Saf* 2005;28:851-70.
337 Myers MG, Cairns JA, Singer J. The consent form as a possible cause of side effects. *Clin Pharmacol Ther* 1987;42:250-3.
338 Wallin J, Sjövall J. Detection of adverse drug reactions in a clinical trial using two types of questioning. *Clin Ther* 1981;3:450-2.
339 Gøtzsche PC. Non-steroidal anti-inflammatory drugs. *BMJ* 2000;320:1058-61.
340 Curfman GD, Morrissey S, Drazen JM. Expression of concern reaffirmed. *N Engl J Med* 2006;354:1193.
341 Wright JM, Perry TL, Bassett KL, Chambers GK. Reporting of 6-month vs 12-month data in a clinical trial of celecoxib. *JAMA* 2001;286:2398-400.
342 Crowe BJ, Xia HA, Berlin JA, Watson DJ, Shi H, Lin SL, et al. Recommendations for safety planning, data collection, evaluation and reporting during drug, biologic and vaccine development: a report of the safety planning, evaluation, and reporting team. *Clin Trials* 2009;6:430-40.
343 Sherman RB, Woodcock J, Norden J, Grandinetti C, Temple RJ. New FDA regulation to improve safety reporting in clinical trials. *N Engl J Med* 2011;365:3-5.
344 Ruiz-Canela M, Martinez-González MA, Gómez-Gracia E, Fernández-Crehuet J. Informed consent and approval by institutional review boards in published reports on clinical trials. *N Engl J Med* 1999;340:1114-5.
345 Breast Cancer International Research Group. BCIRG 006: Multicenter phase III randomized trial comparing doxorubicin and cyclophosphamide followed by docetaxel (AC-->T) with doxorubicin and cyclophosphamide followed by docetaxel and trastuzumab (Herceptin®) (AC-->TH) and with docetaxel, carboplatin and trastuzumab (TCH) in the adjuvant treatment of node positive and high risk node negative patients with operable breast cancer containing the HER2 alteration [protocol]. Version 5 www.nejm.org/doi/full/10.1056/NEJMoa0910383.
346 Getz KA, Zuckerman R, Cropp AB, Hindle AL, Krauss R, Kaitin KI. Measuring the incidence, causes, and repercussions of protocol amendments. *Drug Inf J* 2011;45:265-75.
347 Decullier E, Lhéritier V, Chapuis F. The activity of French research ethics committees and characteristics of biomedical research protocols involving humans: a retrospective cohort study. *BMC Med Ethics* 2005;6:e9.
348 Lösch C, Neuhäuser M. The statistical analysis of a clinical trial when a protocol amendment changed the inclusion criteria. *BMC Med Res Methodol* 2008;8:16.
349 US Food and Drug Administration. Code of federal regulations. Title 21, Vol 5. 21CFR312.30. 2011.
350 European Commission. Communication from the Commission—Detailed guidance on the request to the competent authorities for authorisation of a clinical trial on a medicinal product for human use, the notification of substantial amendments and the declaration of the end of the trial (CT-1) (2010/C 82/01). *Off J European Union* 2010;53.
351 Bond J, Wilson J, Eccles M, Vanoli A, Steen N, Clarke R, et al. Protocol for north of England and Scotland study of tonsillectomy and adeno-tonsillectomy in children (NESSTAC). A pragmatic randomised controlled trial comparing surgical intervention with conventional medical treatment in children with recurrent sore throats [protocol]. *BMC Ear, Nose Throat Disord* 2006;6:13.
352 Williams CJ, Zwitter M. Informed consent in European multicentre randomised clinical trials - Are patients really informed? *Eur J Cancer* 1994;30:907-10.
353 Ryan RE, Prictor MJ, McLaughlin KJ, Hill SJ. Audio-visual presentation of information for informed consent for participation in clinical trials. *Cochrane Database Syst Rev* 2008;1:CD003717.
354 Flory J, Emanuel E. Interventions to improve research participants' understanding in informed consent for research: a systematic review. *JAMA* 2004;292:1593-601.
355 Cohn E, Larson E. Improving participant comprehension in the informed consent process. *J Nurs Scholarsh* 2007;39:273-80.
356 Wendler DS. Assent in paediatric research: theoretical and practical considerations. *J Med Ethic* 2006;32:229.
357 McRae AD, Weijer C, Binik A, Grimshaw JM, Boruch R, Brehaut JC, et al. When is informed consent required in cluster randomized trials in health research? *Trials* 2011;12:202.
358 Beskow LM, Friedman JY, Hardy NC, Lin L, Weinfurt KP. Developing a simplified consent form for biobanking. *PLoS One* 2010;5:e13302.
359 HIV Prevention Trials Network. HPTN 037: A phase III randomized study to evaluate the efficacy of a network-oriented peer educator intervention for the prevention of HIV transmission among injection drug users and their network members [protocol]. Version 2.0 (October 23, 2003). www.hptn.org/research_studies/hptn037.asp.
360 World Association of Medical Editors Editorial Policy and Publication Ethics Committees. Conflict of interest in peer-reviewed medical journals. 2009. www.wame.org/conflict-of-interest-in-peer-reviewed-medical-journals.
361 Rochon PA, Hoey J, Chan A-W, Ferris LE, Lexchin J, Kalkar SR, et al. Financial conflicts of interest checklist 2010 for clinical research studies. *Open Med* 2010;4:e69-91.
362 Drazen JM, de Leeuw PW, Laine C, Mulrow C, DeAngelis CD, Frizelle FA, et al. Towards more uniform conflict disclosures: the updated ICMJE conflict of interest reporting form. *BMJ* 2010;340:c3239.
363 World Medical Association. WMA statement on conflict of interest. 2012. www.wma.net/en/30publications/10policies/i3/.
364 Lundh A, Krogsbøll LT, Gøtzsche PC. Access to data in industry-sponsored trials. *Lancet* 2011;378:1995-6.
365 Microbicide Trials Network. MTN-003: Phase 2B safety and effectiveness study of tenofovir 1% gel, tenofovir disproxil fumarate tablet and emtricitabine/tenofovir disoproxil fumarate tablet for the prevention of HIV infection in women [protocol]. Version 2.0 (December 31, 2010). www.mtnstopshiv.org/news/studies/mtn003.
366 Richardson HS, Belsky L. The ancillary-care responsibilities of medical researchers. *Hastings Center Report* 2004;34:25-33.
367 Belsky L, Richardson HS. Medical researchers' ancillary clinical care responsibilities. *BMJ* 2004;328:1494-6.
368 Sofaer N, Strech D. Reasons why post-trial access to trial drugs should, or need not be ensured to research participants: A systematic review. *Public Health Ethics* 2011;4:160-84.
369 Participants in the 2006 Georgetown University Workshop on the Ancillary-Care Obligations of Medical Researchers Working in Developing Countries. The ancillary-care obligations of medical researchers working in developing countries. *PLoS Med* 2008;5:e90.
370 Beta-Blocker Evaluation of Survival Trial (BEST) Protocol [protocol]. Version 1 (June 22, 1999). https://biolincc.nhlbi.nih.gov/studies/best/.
371 Mann H. Research ethics committees and public dissemination of clinical trial results. *Lancet* 2002;360:406-8.
372 Gøtzsche PC. Why we need easy access to all data from all clinical trials and how to accomplish it. *Trials* 2011;12:249.
373 Whittington CJ, Kendall T, Fonagy P, Cottrell D, Cotgrove A, Boddington E. Selective serotonin reuptake inhibitors in childhood depression: systematic review of published versus unpublished data. *Lancet* 2004;363:1341-5.
374 Cowley AJ, Skene A, Stainer K, Hampton JR. The effect of lorcainide on arrhythmias and survival in patients with acute myocardial infarction: an example of publication bias. *Int J Cardiol* 1993;40:161-6.
375 McGauran N, Wieseler B, Kreis J, Schüler YB, Kölsch H, Kaiser T. Reporting bias in medical research - a narrative review. *Trials* 2010;11:37.
376 Hart B, Lundh A, Bero L. Effect of reporting bias on meta-analyses of drug trials: reanalysis of meta-analyses. *BMJ* 2012;344:d7202.
377 Doshi P, Jones M, Jefferson T. Rethinking credible evidence synthesis. *BMJ* 2012;344:d7898.
378 Emerson GB, Warme WJ, Wolf FM, Heckman JD, Brand RA, Leopold SS. Testing for the presence of positive-outcome bias in peer review: a randomized controlled trial. *Arch Intern Med* 2010;170:1934-9.
379 Olson CM, Rennie D, Cook D, Dickersin K, Flanagin A, Hogan JW, et al. Publication bias in editorial decision making. *JAMA* 2002;287:2825-8.
380 Rochon PA, Sekeres M, Hoey J, Lexchin J, Ferris LE, Moher D, et al. Investigator experiences with financial conflicts of interest in clinical trials. *Trials* 2011;12:9.
381 Steinbrook R. Gag clauses in clinical-trial agreements. *N Engl J Med* 2005;352:2160-2.
382 McCarthy M. Company sought to block paper's publication. *Lancet* 2000;356:1659.
383 Nathan DG, Weatherall DJ. Academic freedom in clinical research. *N Engl J Med* 2002;347:1368-71.
384 Rennie D. Thyroid storm. *JAMA* 1997;277:1238-43.
385 Flanagin A, Fontanarosa PB, DeAngelis CD. Authorship for research groups. *JAMA* 2002;288:3166-8.
386 Ross JS, Hill KP, Egilman DS, Krumholz HM. Guest authorship and ghostwriting in publications related to rofecoxib: a case study of industry documents from rofecoxib litigation. *JAMA* 2008;299:1800-12.
387 Wislar JS, Flanagin A, Fontanarosa PB, DeAngelis CD. Honorary and ghost authorship in high impact biomedical journals: a cross sectional survey. *BMJ* 2011;343:d6128.
388 Gøtzsche PC, Kassirer JP, Woolley KL, Wager E, Jacobs A, Gertel A, et al. What should be done to tackle ghostwriting in the medical literature? *PLoS Med* 2009;6:e1000023.
389 International Committee of Medical Journal Editors. Uniform requirements for manuscripts submitted to biomedical journals: Writing and editing for biomedical publication. 2010. www.icmje.org/urm_full.pdf.
390 Matheson A. How industry uses the ICMJE guidelines to manipulate authorship--and how they should be revised. *PLoS Med* 2011;8:e1001072.
391 Graf C, Battisti WP, Bridges D, Bruce-Winkler V, Conaty JM, Ellison JM, et al. Good publication practice for communicating company sponsored medical research: the GPP2 guidelines. *BMJ* 2009;339:b4330.
392 Jacobs A, Wager E. European Medical Writers Association (EMWA) guidelines on the role of medical writers in developing peer-reviewed publications. *Curr Med Res Opin* 2005;21:317-21.
393 Wolinsky FD, Vander Weg MW, Howren MB, Jones MP, Martin R, Luger TM, et al. Protocol for a randomized controlled trial to improve cognitive functioning in older adults: the Iowa Healthy and Active Minds Study [protocol]. *BMJ Open* 2011;1:e000218.

chaa006386.indd 41 31/01/2013 10:33:40

394 Chan A-W. Bias, spin, and misreporting: Time for full access to trial protocols and results. *PLoS Med* 2008;5:e230.
395 Lassere M, Johnson K. The power of the protocol. *Lancet* 2002;360:1620-2.
396 Wieseler B, Kerekes MF, Vervoelgyi V, McGauran N, Kaiser T. Impact of document type on reporting quality of clinical drug trials: a comparison of registry reports, clinical study reports, and journal publications. *BMJ* 2012;344:d8141.
397 Gøtzsche PC, Jørgensen AW. Opening up data at the European Medicines Agency. *BMJ* 2011;342:d2686.
398 European Medicines Agency. European Medicines Agency policy on access to documents (related to medicinal products for human and veterinary use) (EMA/110196/2006). 2010. www.ema.europa.eu/docs/en_GB/document_library/Other/2010/11/WC500099473.pdf.
399 Doshi P, Jefferson T, Del Mar C. The imperative to share clinical study reports: recommendations from the tamiflu experience. *PLoS Med* 2012;9:e1001201.
400 Eichler H-G, Abadie E, Breckenridge A, Leufkens H, Rasi G. Open clinical trial data for all? A view from regulators. *PLoS Med* 2012;9:e1001202.
401 Committee on Responsibilities of Authorship in the Biological Sciences, National Research Council. Sharing publication-related data and materials: responsibilities of authorship in the life sciences. National Academies Press, 2003.
402 Hrynaszkiewicz I, Norton ML, Vickers AJ, Altman DG. Preparing raw clinical data for publication: guidance for journal editors, authors, and peer reviewers. *Trials* 2010;11:9.
403 Walport M, Brest P. Sharing research data to improve public health. *Lancet* 2011;377:537-9.
404 Ross JS, Lehman R, Gross CP. The importance of clinical trial data sharing: toward more open science. *Circ Cardiovasc Qual Outcomes* 2012;5:238-40.
405 Vickers AJ. Making raw data more widely available. *BMJ* 2011;342:d2323.
406 The Royal Society Science Policy Centre. Science as an open enterprise. 2012. http://royalsociety.org/uploadedFiles/Royal_Society_Content/policy/projects/sape/2012-06-20-SAOE.pdf
407 Summerskill W, Collingridge D, Frankish H. Protocols, probity, and publication. *Lancet* 2009;373:992.
408 Altman D, Furberg C, Grimshaw J, Rothwell P. Trials—using the opportunities of electronic publishing to improve the reporting of randomised trials. *Trials* 2006;7:6.
409 Sharing of materials, methods, and data. 2011. www.plosone.org/static/policies.action.
410 Trials. Instructions for authors. Editorial policies. 2012. www.trialsjournal.com/authors/instructions.
411 National Institutes of Health. Final NIH statement on sharing research data. Feb 26, 2003. http://grants.nih.gov/grants/guide/notice-files/NOT-OD-03-032.html.
412 Laine C, Goodman SN, Griswold ME, Sox HC. Reproducible research: moving toward research the public can really trust. *Ann Intern Med* 2007;146:450-3.
413 BMJ Publishing Group Ltd. Instructions for authors. 2012. http://bmjopen.bmj.com/site/about/guidelines.xhtml.
414 Sugarman J, McCrory DC, Hubal RC. Getting meaningful informed consent from older adults: a structured literature review of empirical research. *J Am Ger Soc* 1998;46:517-24.
415 Paris A, Cracowski JL, Ravanel N, Cornu C, Gueyffier F, Deygas B, et al. [Readability of informed consent forms for subjects participating in biomedical research: updating is required]. *Presse Med* 2005;34:13-8.
416 Southwest Oncology Group. Chemoprevention of prostate cancer with finasteride (Proscar®) Phase III [protocol]. Aug 2001 version. http://swog.org/visitors/pcpt/.
417 Schulz KF, Altman DG, Moher D, the CONSORT Group. CONSORT 2010 Statement: updated guidelines for reporting parallel group randomised trials. *BMJ* 2010;340:c332.

chaa006386.indd 42 31/01/2013 10:33:40

Reproduced with permission of copyright owner.
Further reproduction prohibited without permission.
